# Supplementary material for: Identifying dysregulated regions in amyotrophic lateral sclerosis through chromatin accessibility outliers
Source: HGG Adv. 2024 Jun 13;5(3):100318. doi: 10.1016/j.xhgg.2024.100318 (PMC11260578; doi:10.1016/j.xhgg.2024.100318)
Supplement: Document S1. Figures S1–S17 and Algorithm S1 [file mmc1.pdf]

HGGA, Volume *5*

Supplemental information

Identifying dysregulated regions in amyotrophic lateral sclerosis through chromatin accessibility outliers

Muhammed Hasan Çelik, Julien Gagneur, Ryan G. Lim, Jie Wu, Leslie M. Thompson, and Xiaohui Xie

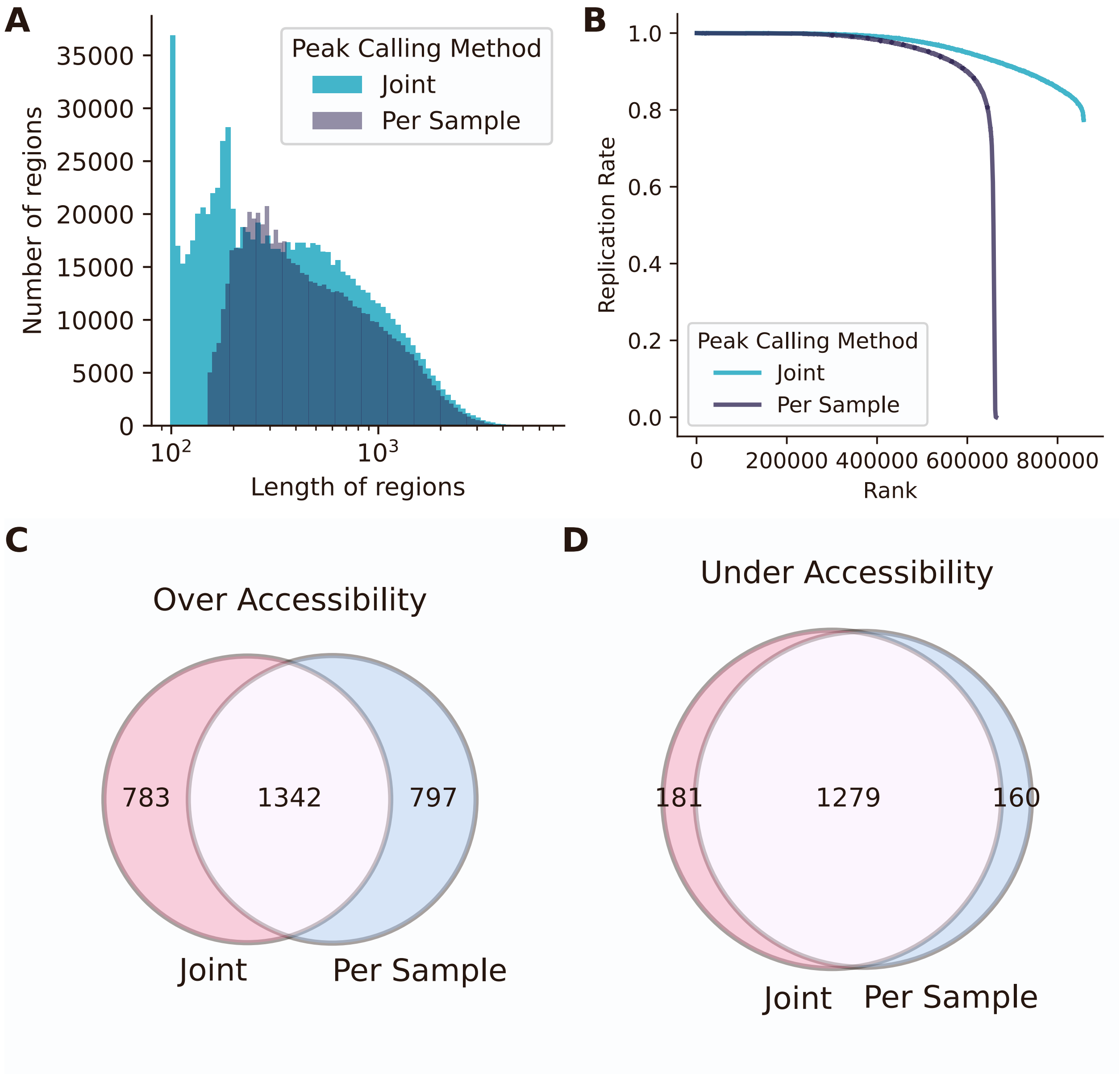

Figure S1. The comparison of joint and per-sample peak calling methods

Peaks are identified using MACS2 in both approaches. For the cohort-wide joint peak calling, all ATAC-seq reads are aggregated into a single BAM file, which is subsequently processed with MACS2 to produce a set of peaks consistent across samples. In the sample-wise peak calling approach, peaks are identified individually for each sample using MACS2, resulting in a separate set of peaks for each sample. Then, overlapping peaks from different samples are collapsed, ensuring a consistent comparison of peaks during the outlier calling. **(A)** The length distribution (in base pairs) of accessible chromatin regions detected with joint and per-sample peak calling. Joint peak calling produces a greater number of peaks that are narrower compared to per-sample peak calling. **(B)** The moving average of the replication rate by the rank of peaks, sorted by total read counts. Joint peak calling exhibits a higher replication rate in regions with lower read depth. **(C)** Overlap of over-accessibility outliers identified through joint and per-sample peak calling methods. Both approaches generate a comparable number of over-accessibility outliers. ~63% of the peaks identified by one method were detected by the other method. **(D)** Overlap

of under-accessibility outliers identified through joint and per-sample peak calling methods. ~88% of the outliers identified by one method were also detected by the other method.

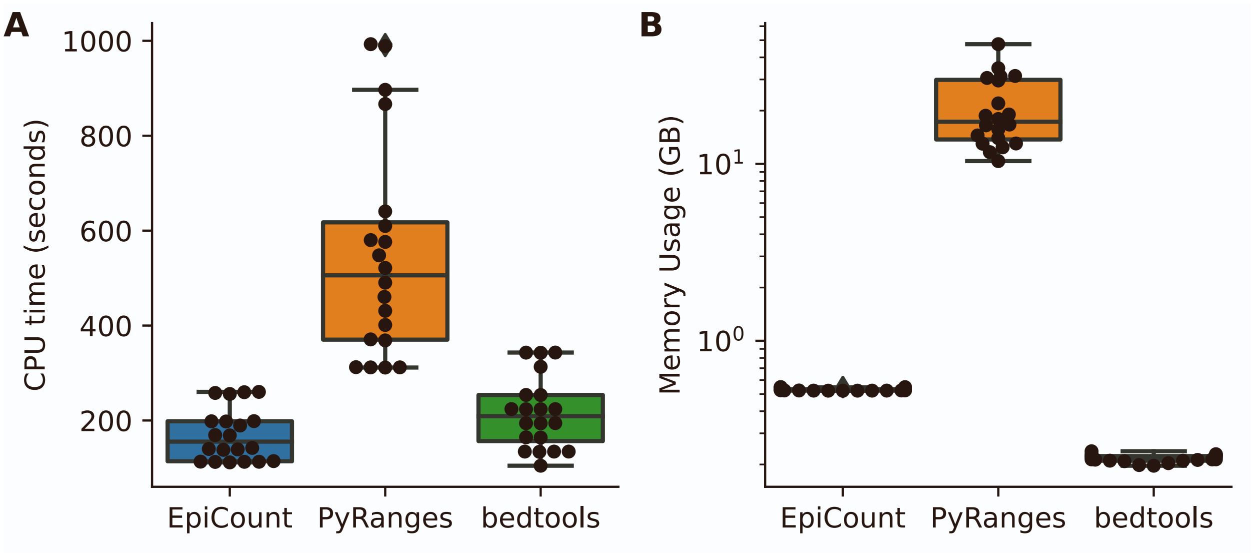

Figure S2. Runtime of counting step.

Bam and bed files for 20 ATAC-seq experiments were downloaded from ENCODE. Each bam file has 142 ± 48 million ATAC-seq reads. Overlapping accessible regions across bed files were collapsed, leading to $365{,}090$ accessible regions. The counting was performed with these accessible regions and bam files using EpiCount, bedtools, and PyRanges. During bedtools counting, bed files are first sorted, then counting is performed using the *-sorted* flag in bedtools to leverage the chrom-sweep algorithm. **(A)** CPU time of counting methods. EpiCount is twice as fast as PyRanges and comparable with bedtools. The mean runtime of EpiOut per bam file is 169 ± 54 seconds; meanwhile, the runtime is 549 ± 225 seconds for PyRanges and 214 ± 74 for bedtools. Epiout counts one million reads per 1.2 ± 0.1 seconds, while PyRanges counts one million reads in 3.8 ± 0.2 seconds and bedtools counts in 1.5 ± 0.1. **(B)** EpiOut and bedtools have a much smaller memory footprint than PyRanges. PyRanges loads the entire bam file to memory while EpiCount and bedtools perform stream counting by only loading a read per iteration; thus, EpiCount (mean memory consumption 0.53 ± 0.009 GB) and bedtools (mean memory consumption 0.21 ± 0.01 GB) consumes $\sim 2\%$ of PyRanges' memory footprint (mean memory consumption 21 ± 10 GB). Extensive memory usage of PyRanges limits the parallelization across samples.

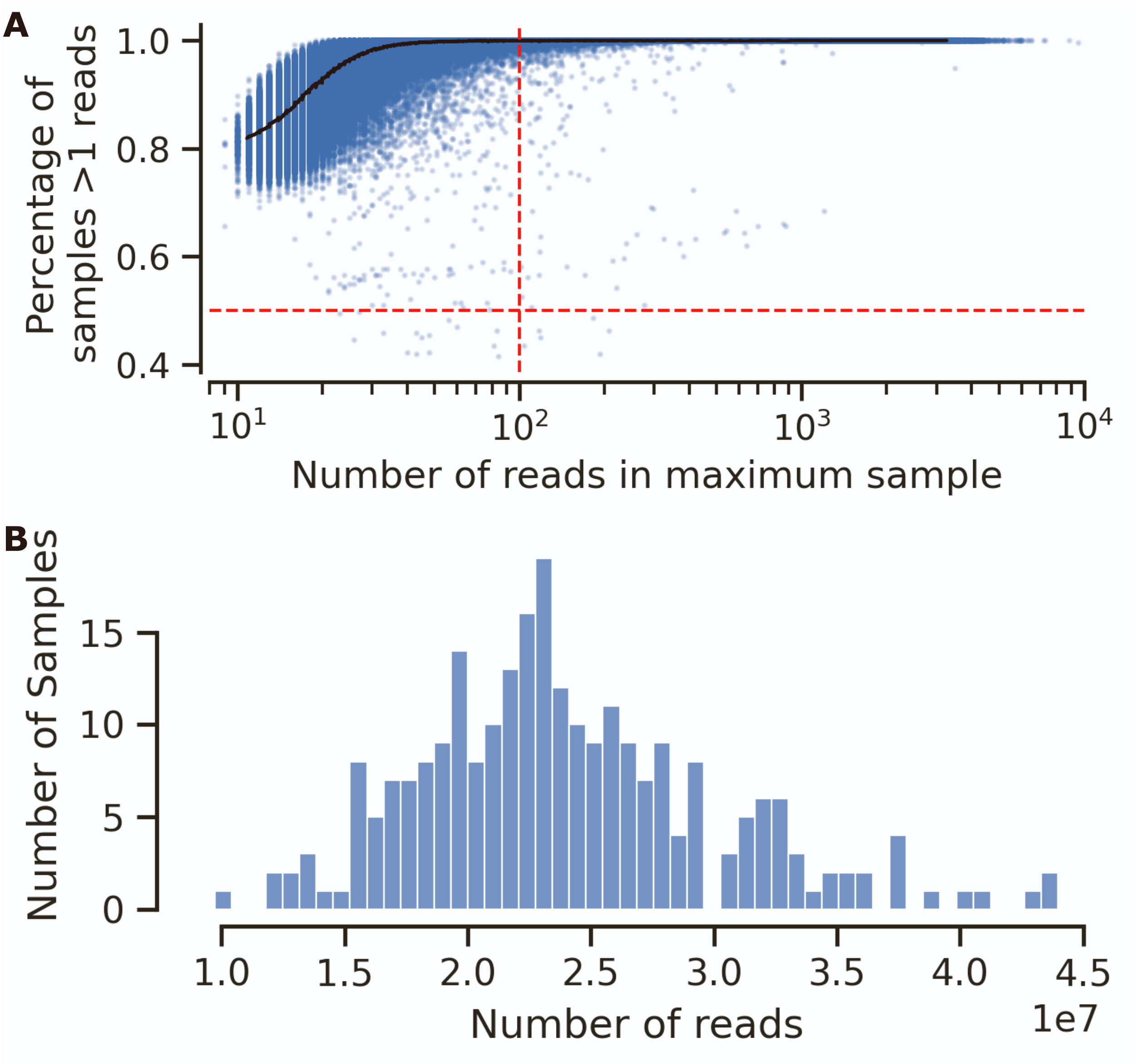

Figure S3. Read coverage and replication rate statistics.

(A) Read coverage distribution across samples. Notable differences in read coverage are observed among the samples, highlighting the importance of size factor normalization to adjust for coverage disparity. **(B)** Replication rate of accessible regions throughout samples. In the scatterplot, each dot signifies an accessible region for a sample. The x-axis denotes the read counts supporting the accessible region in the sample with the highest read count. Filters, represented by the red lines, were implemented to ensure an accessible region is supported by at least 100 reads in one sample and is replicated in at least 50% of the samples by at least 2 reads.

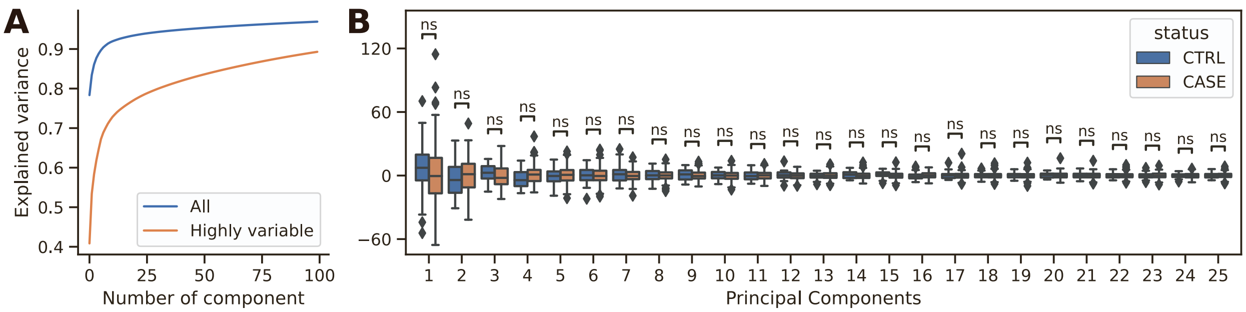

Figure S4. The major covariates in the DNA accessibility data.
(A) Cumulative explained variance by top principal components. The variance of accessibility is calculated based on the raw read counts, and the top 20,000 regions with the highest variance are chosen as highly variable regions. Then, the read counts are normalized for variance stabilizing transformation (VST), and principal component analysis is performed. The top 25 principal components (representing the major latent confounding factors) explain ~79% and ~94% of the accessibility covariation between samples for highly variable and all regions, respectively. **(B)** The distribution of top principal components by the disease status for the highly variable regions. None of the top principal components are associated with the disease status based on the Mann–Whitney U test. P-values are corrected for multiple testing with the Benjamini-Yekutieli false-discovery rate (FDR) method.

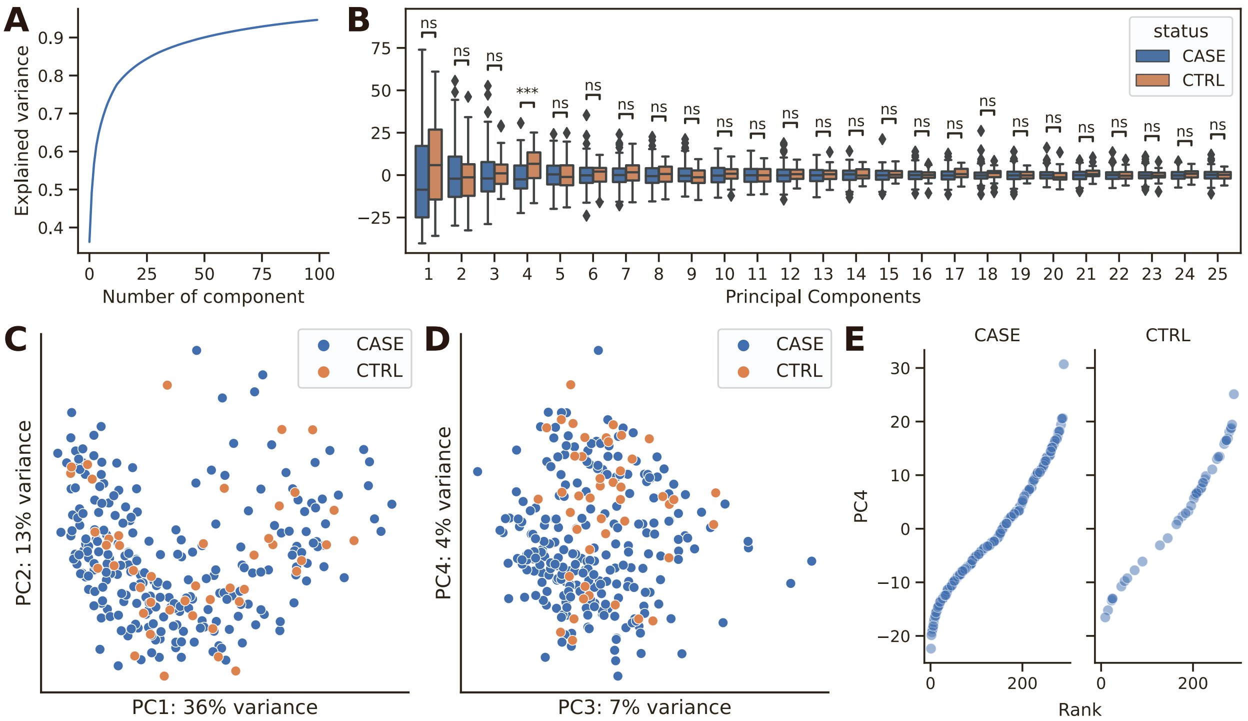

Figure S5. The major covariates in the transcriptomics data.

(A) Cumulative explained variance by top principal components. The variance of gene expression is calculated based on the raw read counts, and the top 10,000 genes with the highest variance are chosen. Then, the read counts are normalized for variance stabilizing transformation (VST), and principal component analysis is performed. The top 25 principal components explain ~79% of the gene expression covariation between samples. **(B)** The distribution of top principal components by the disease status. Only one of the top principal components is associated with the disease status based on the Mann–Whitney U test. P-values are corrected for multiple testing with the Benjamini-Yekutieli false-discovery rate (FDR) method. **(C)** The first, second **(D),** and the third and fourth principal components. **(E)** The distribution of samples by the fourth principal component has slightly higher values for control samples; however, a clear separation between cases and controls is not observed.

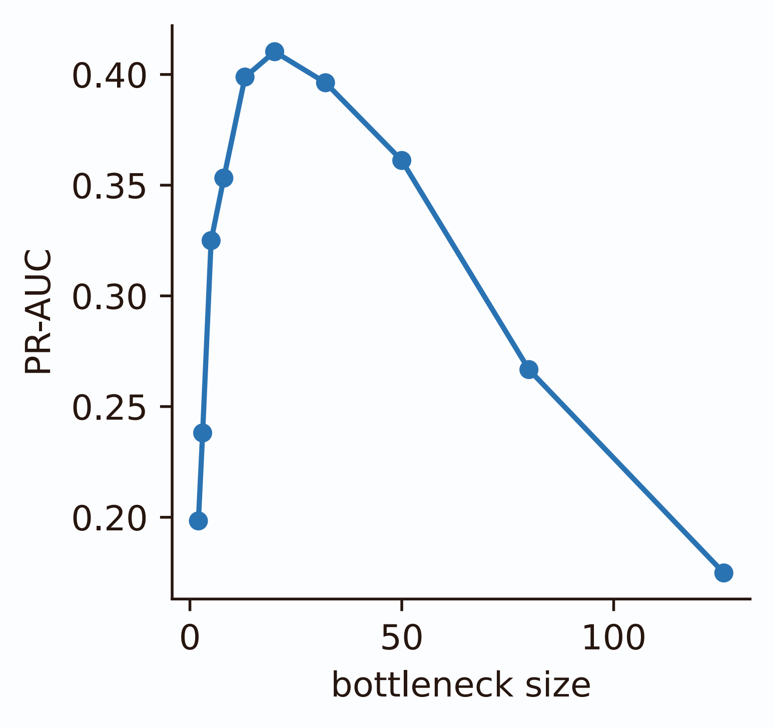

Figure S6. Optimal bottleneck size choice with hyperparameter tuning.
EpiOut detects optimal bottleneck size with artificial outlier injection and hyperparameter tuning. The x-axis indicates the bottleneck size of LR-AE in the EpiOut model, and the y-axis shows the auPRC performance for the artificial outlier prediction.

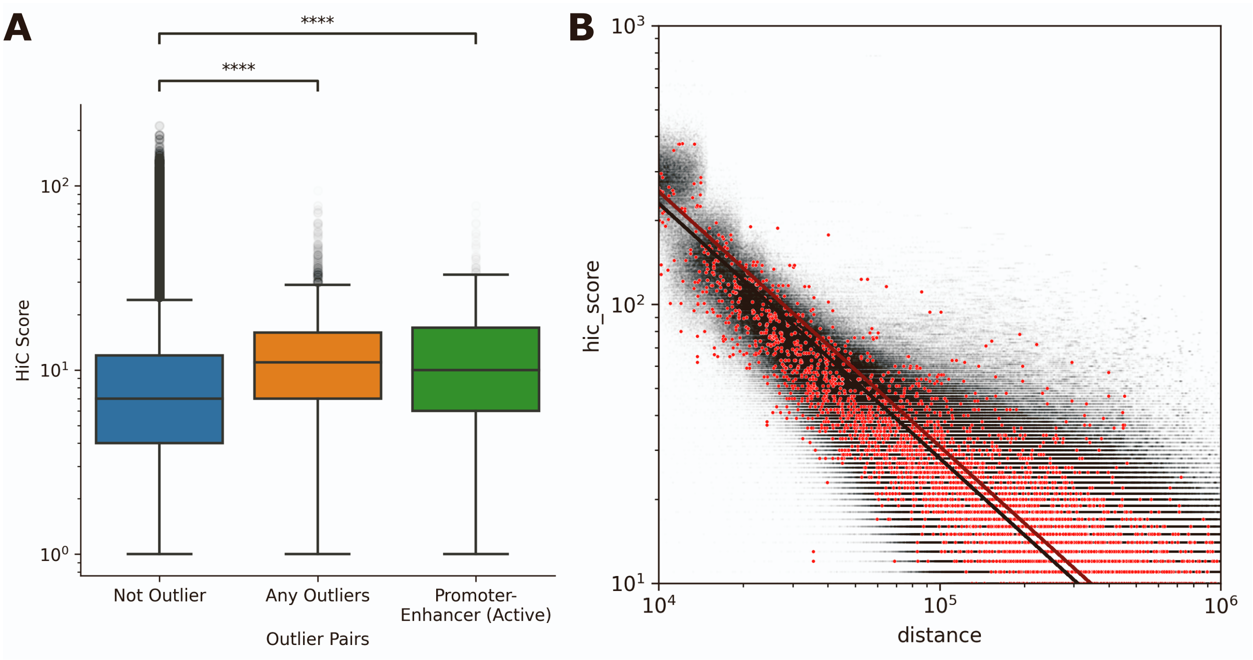

Figure S7. Interaction between outlier pairs.

(A) Hi-C score distribution of accessible regions, which are at least 100 kilo-bp apart, by outlier status and annotation. P-values were calculated with the U-Mann Wily test and corrected for multiple testing using the Bonferroni method. **(B)** Log-log shows the Hi-C scores of non-outlier pairs (in black) and outlier pairs (in red). Hi-C scores decay by power law with increasing distances. We fit a power regression (indicated by black and red lines) on the data to test the interaction between outlier pairs while using distance as a control variable.

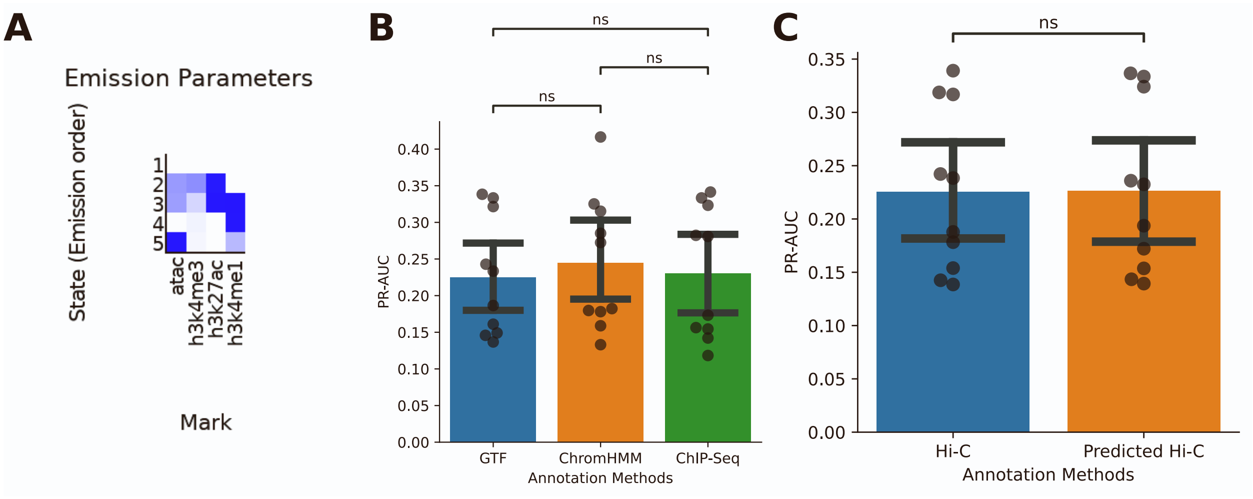

Figure S8. The benchmark of chromatin annotation methods to predict gene expression outliers. **(A)** Emission parameters of ChromHMM. The genome is divided into 200 bp segments and annotated with ChromHMM using 5 hidden states and features of H3K4me3, H3K27ac, H3K4me1, and ATAC-seq. The accessible regions are annotated for one or more states based on their overlap with the segments. **(B)** The performance of alternative annotation methods to predict gene expression outliers. The EBM model was trained and tested with 10-fold cross-validation using the relevant features in each annotation setup**.** The model based on GTF annotation (blue) employs features such as log fold change, p-value, TSS outlier status (as determined by GENCODE annotation), maximum absolute log fold change of proximal chromatin accessibility outliers, and ABC-score weighted absolute log fold change of distal outliers. In contrast, the model utilizing ChromHMM (orange) integrates the same set of features as the blue model, but it further disaggregates the TSS, proximal, and distal features into five distinct features, each corresponding to a specific hidden state identified by ChromHMM. Similarly, the model based on the ChIP-seq marks (green) uses the same features but subdivides the features by promoter, active enhancer, poised enhancer, and region with no chromatin mark. There is no statistically significant difference ($ns \geq 0.05$) between models to predict gene expression outliers based on the Mann–Whitney U test (the PR-AUC for blue $= 22.4 \pm 8.1\%$, orange $= 24.4 \pm 9.1\%$, and green $= 23.1 \pm 8.9\%$). **(C)** The evaluation of gene expression prediction performance, utilizing the ABC-score, was conducted using both observed Hi-C scores (blue) and predicted Hi-C scores (orange). A power regression model (Figure S5B) was employed to compute the Hi-C scores for outlier pairs within a distance of up to 1 million base pairs. There is no statistically significant difference (ns≥0.05) between models to predict gene expression outliers based on the Mann–Whitney U test (PR-AUC for blue $= 22.5 \pm 7.7\%$ and orange $= 22.6 \pm 7.9\%$). The findings suggest that the co-outlier status serves as adequate evidence to infer interactions between accessible regions for predicting gene expression outliers.

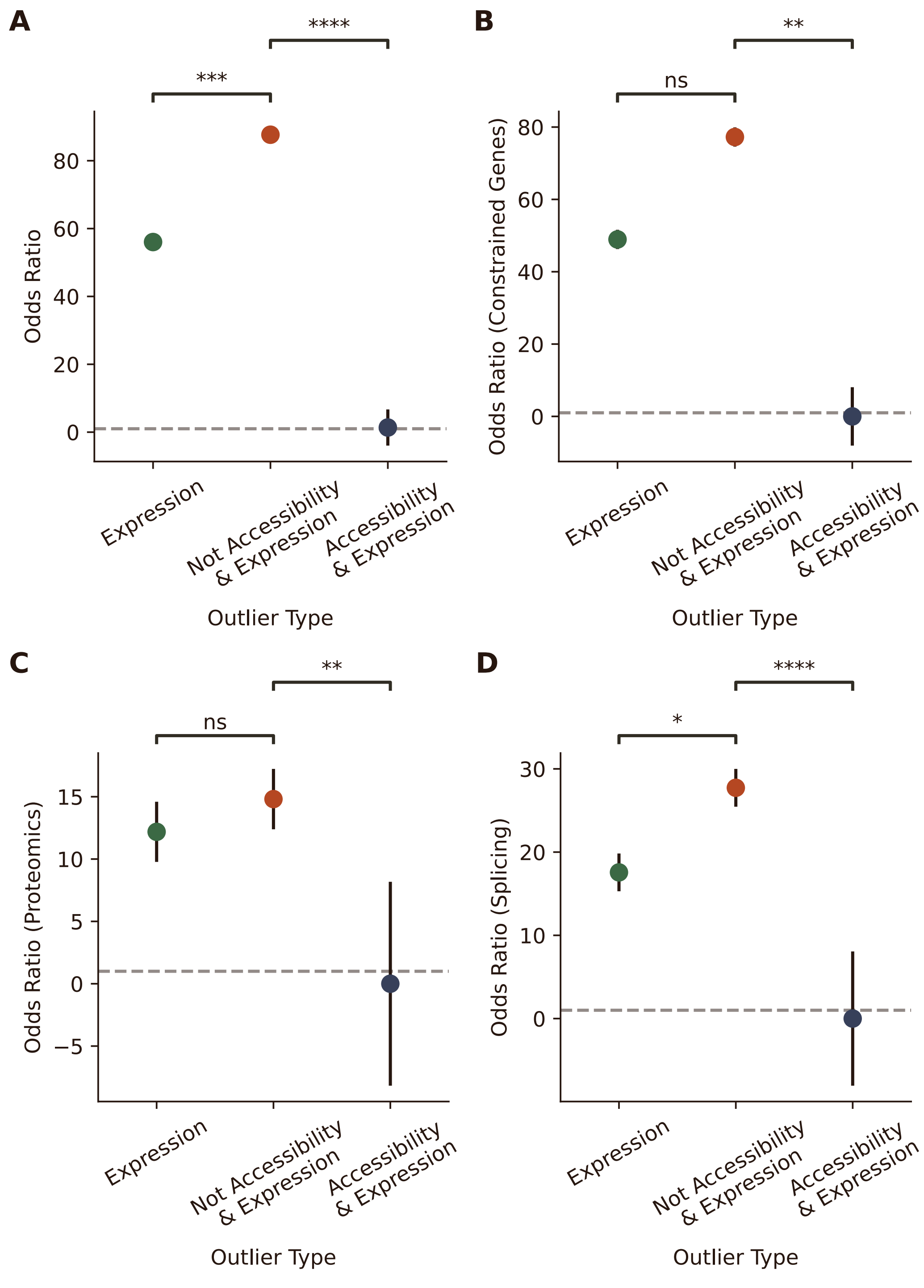

Figure S9: The enrichment and depletion of variants across outlier types by variant effect category. **(A)** Odds of observing potentially NMD-triggering variants in expression outliers (green), expression outliers with non-outlier promoters (red), and expression outliers with outlier promoters are presented. Expression outliers with outlier promoters are strongly depleted of potentially NMD-triggering variants compared to those with non-outlier promoters. Meanwhile, expression outliers with non-outlier promoters are significantly enriched for NMD-triggering variants when compared to the baseline comprising all expression outliers. **(B)** Analysis similar to panel-A but limited to genes under strong

constraint ($\mathrm{LOEUF} < 35\%$) replicates consistent depletion in expression outliers with outlier promoters compared to expression outliers with non-outlier promoters. **(C)** Among genes exhibiting proteomic aberrations (defined by $|Z\text{-score}| > 1$), missense and potentially NMD-triggering rare variants show depletion in genes with outlier promoters relative to gene expression outliers with non-outlier promoters. **(D)** Splicing-disrupting variants predicted by AbSplice are enriched in genes with non-outlier promoters against all gene expression outliers. In contrast, genes with outlier promoters are strongly depleted for the potentially splicing-disrupting variants. ($ns \geq 0.05$,$*< 0.05$,$**< 10^{-2}$,$***< 10^{-3}$,$****< 10^{-4}$ based on the hypergeometric test).

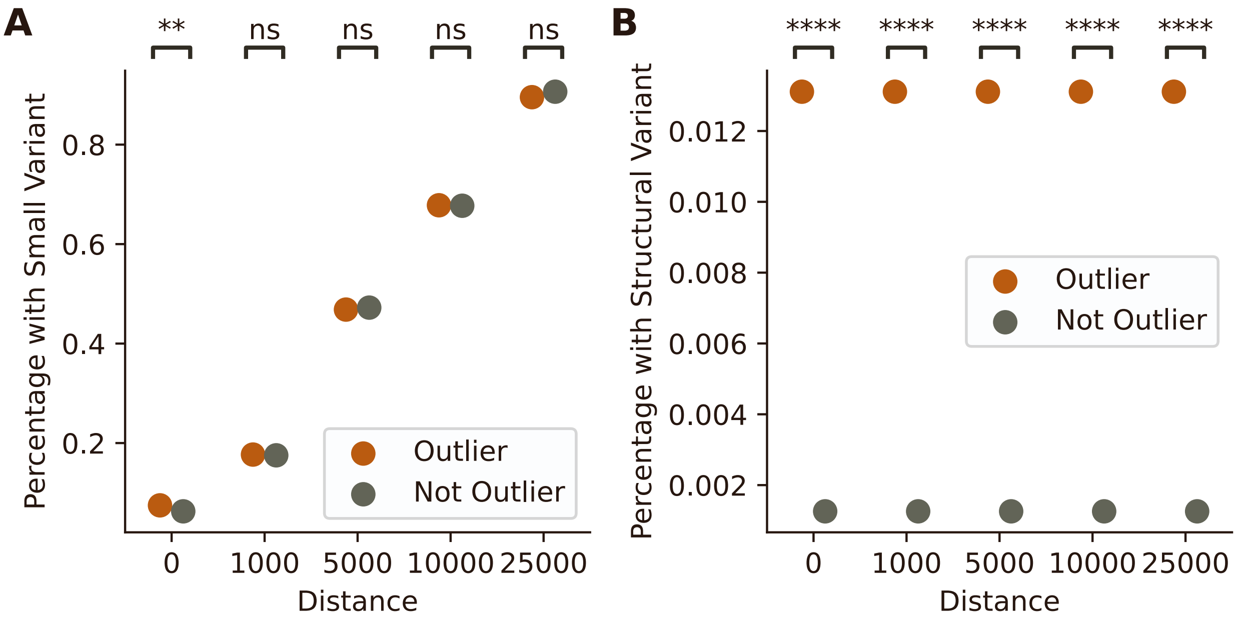

Figure S10: The enrichment of variants in the vicinity of chromatin accessibility outliers (A) Small variants such as SNVs or indels in the accessible chromatin regions are slightly enriched for low frequency and rare variants ($P = 0.002, n = 268, odds\ ratio = 1.2$). However, variants in the vicinity of the chromatin accessibility outliers are not enriched compared to non-outliers. The small effect size can be explained by the fact that the majority of these variants are non-coding and are likely to have weak or neutral regulatory effects. **(B)** Chromatin accessibility outliers are more likely to occur near structural variants ($P < 0.001, n = 47, odds\ ratio = 10.5$). However, only $\sim 1\%$ of chromatin accessibility outliers have at least one structural variant in 1 kbp distance, and $\sim 1.3\%$ have structural variants in proximity.

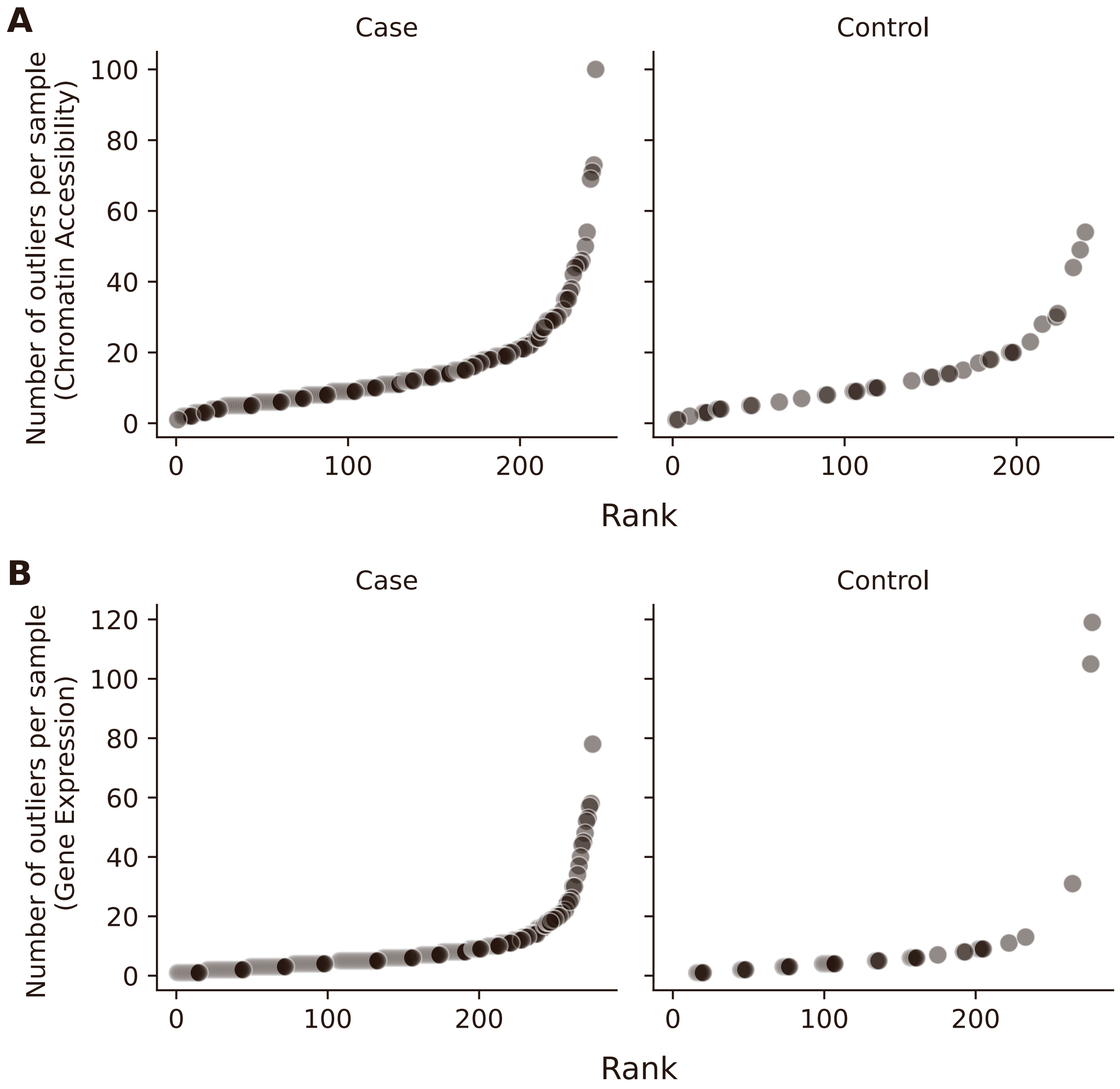

Figure S11: Number of chromatin accessibility and gene expression outliers detected per sample. **(A)** Number of chromatin accessibility outliers per ALS case (11 outliers in median case) **(B)** and clinically healthy controls (10 outliers in median control sample). There is no statistically significant difference in the number of outliers detected between cases and controls ($P = 0.69$, based on the Mann–Whitney U test). **(C)** Number of gene expression outliers per ALS case (6 outliers in median case) **(D)** and controls (4 outliers in median control sample). There is no statistically significant difference in the number of outliers detected between cases and controls ($P = 0.054$, based on the Mann–Whitney U test).

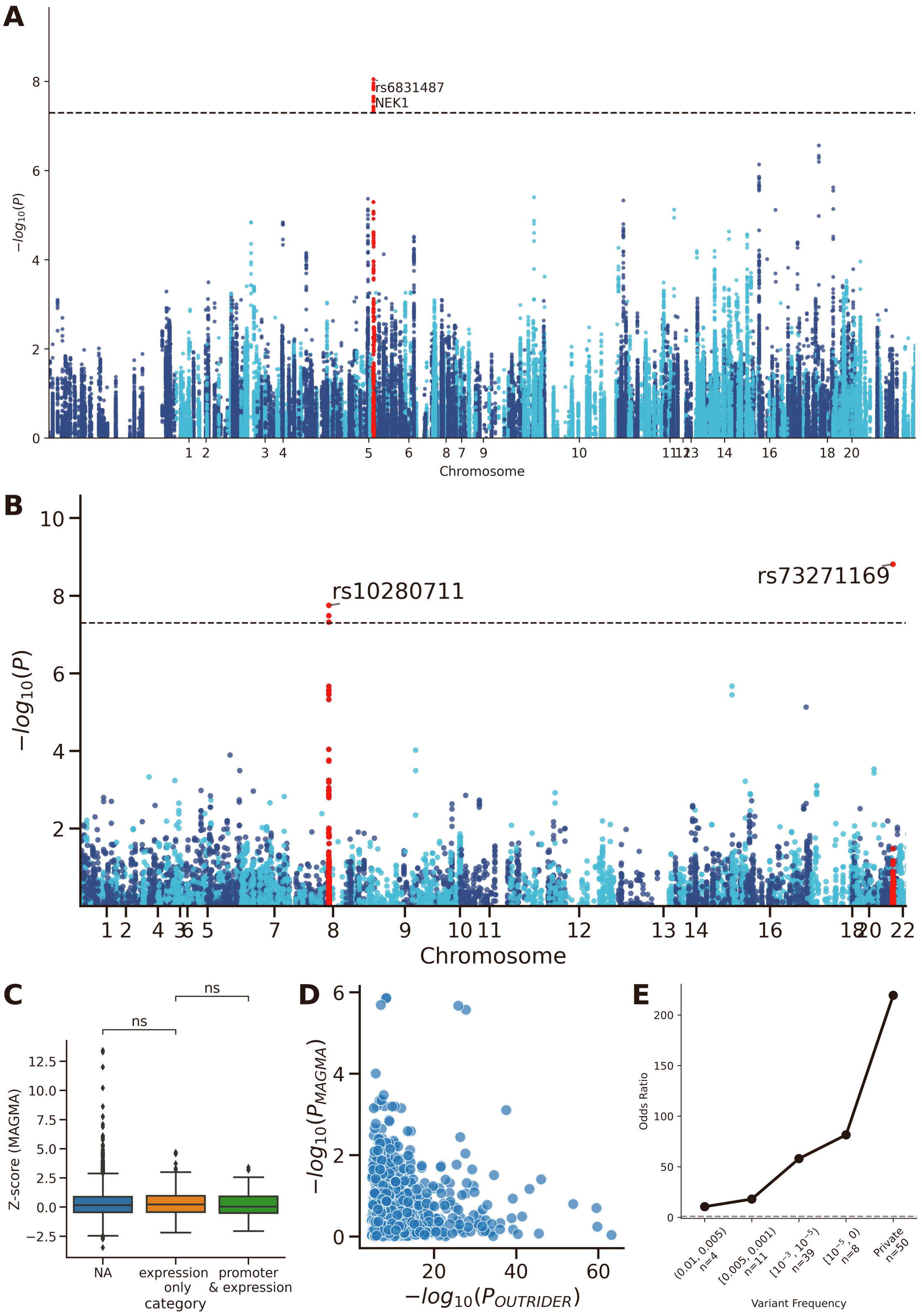

Figure S12: (A) Manhattan plot of variants located among expression outlier genes, indicating loci associated with ALS. The variants are included if observed in the sample with the gene expression outliers. Only one significant locus was found, marked by the most significant variant of rs6831487 near

the NEK1 gene. However, no chromatin accessibility outlier is observed in the vicinity. **(B)** Manhattan plot presenting variants near chromatin accessibility outliers, with two loci identified by variants rs10280711 and rs73271169 in the vicinity of a chromatin accessibility outlier in two ALS cases. However, no gene expression outlier is observed in the vicinity. **(C)** MAGMA Z-score distribution across genes, categorized by outlier status. There is no statistically significant difference between non-outlier genes and the expression of outlier genes with or without outlier promoters. **(D)** OUTRIDER and MAGMA p-values for genes. Genes prioritized based on GWAS and outlier analysis are mostly orthogonal. **(E)** Odds of observing a potentially NMD-triggering rare variant with a specific frequency in a gene expression outlier. The enrichment of rare and private potentially NMD-triggering variants is stronger than the enrichment of low-frequency variants. The results indicated that outlier analysis prioritized the molecular effect of ultra-rare variants, which GWAS may not prioritize.

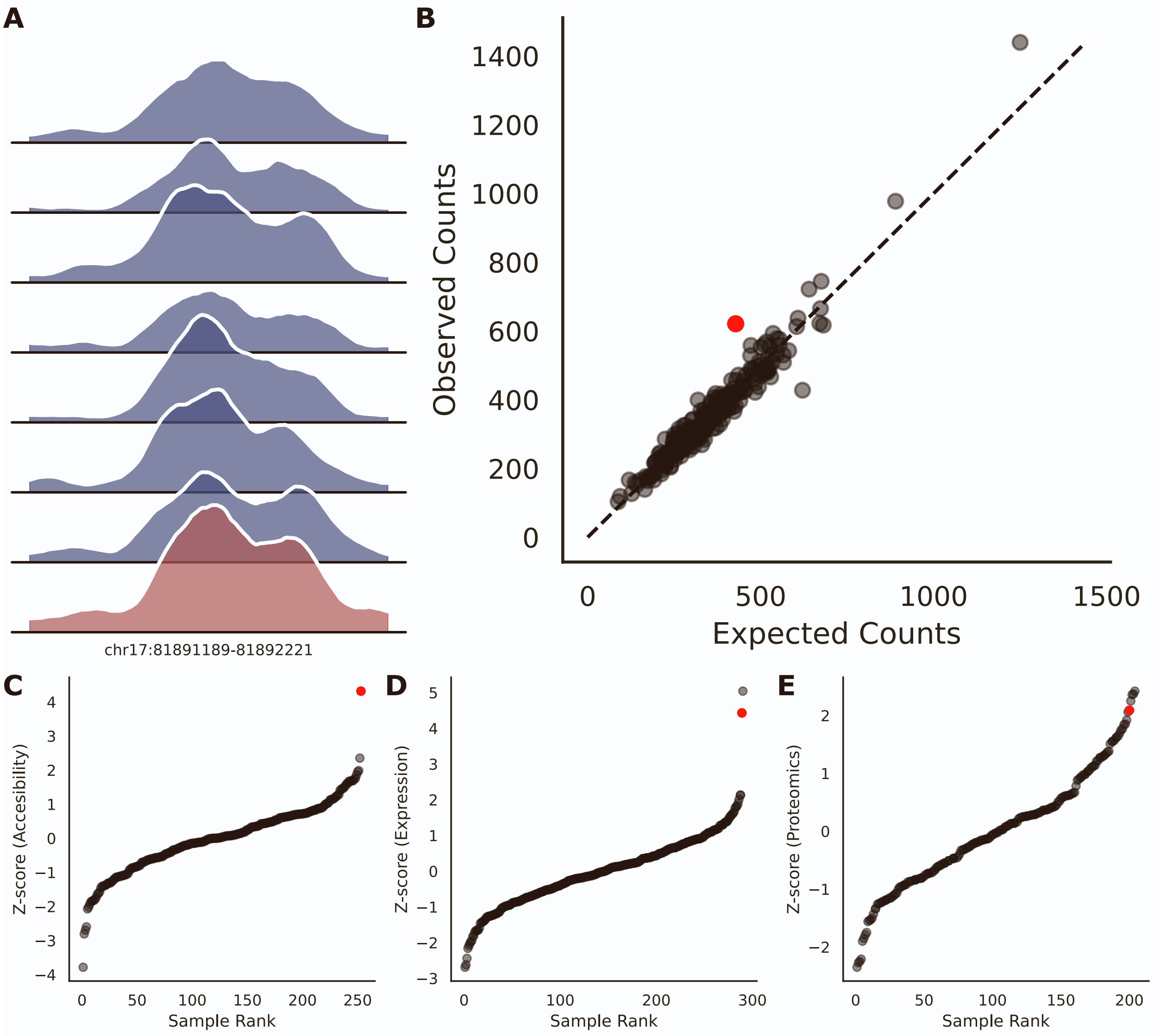

Figure S13. Aberrations observed at multiple omics levels of *ALYREF* gene.
(A) ATAC-seq read coverage at the promoter of the gene. The sample with an outlier promoter is indicated by red across panels. **(B)** Expected and observed accessibility in the promoter of the gene across samples **(C)** Z-score distribution of promoter accessibility **(D)** gene expression **(E)** protein levels across samples.

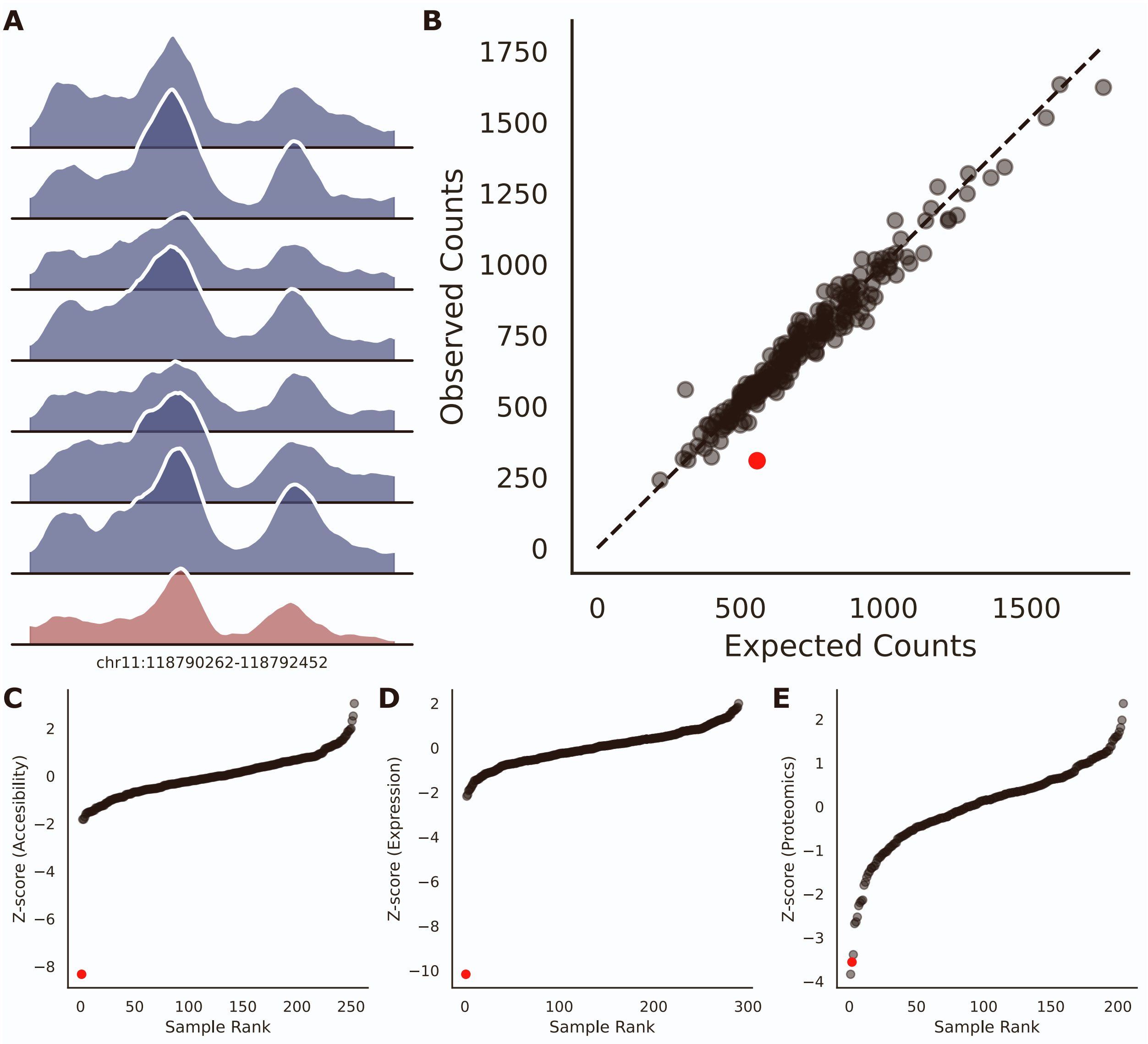

Figure S14. Aberrations observed at multiple omics levels of *DDX6* gene.
(A) ATAC-seq read coverage at the promoter of the gene. The sample with an outlier promoter is indicated by red across panels. **(B)** Expected and observed accessibility in the promoter of the gene across samples **(C)** Z-score distribution of promoter accessibility **(D)** gene expression **(E)** protein levels across samples.

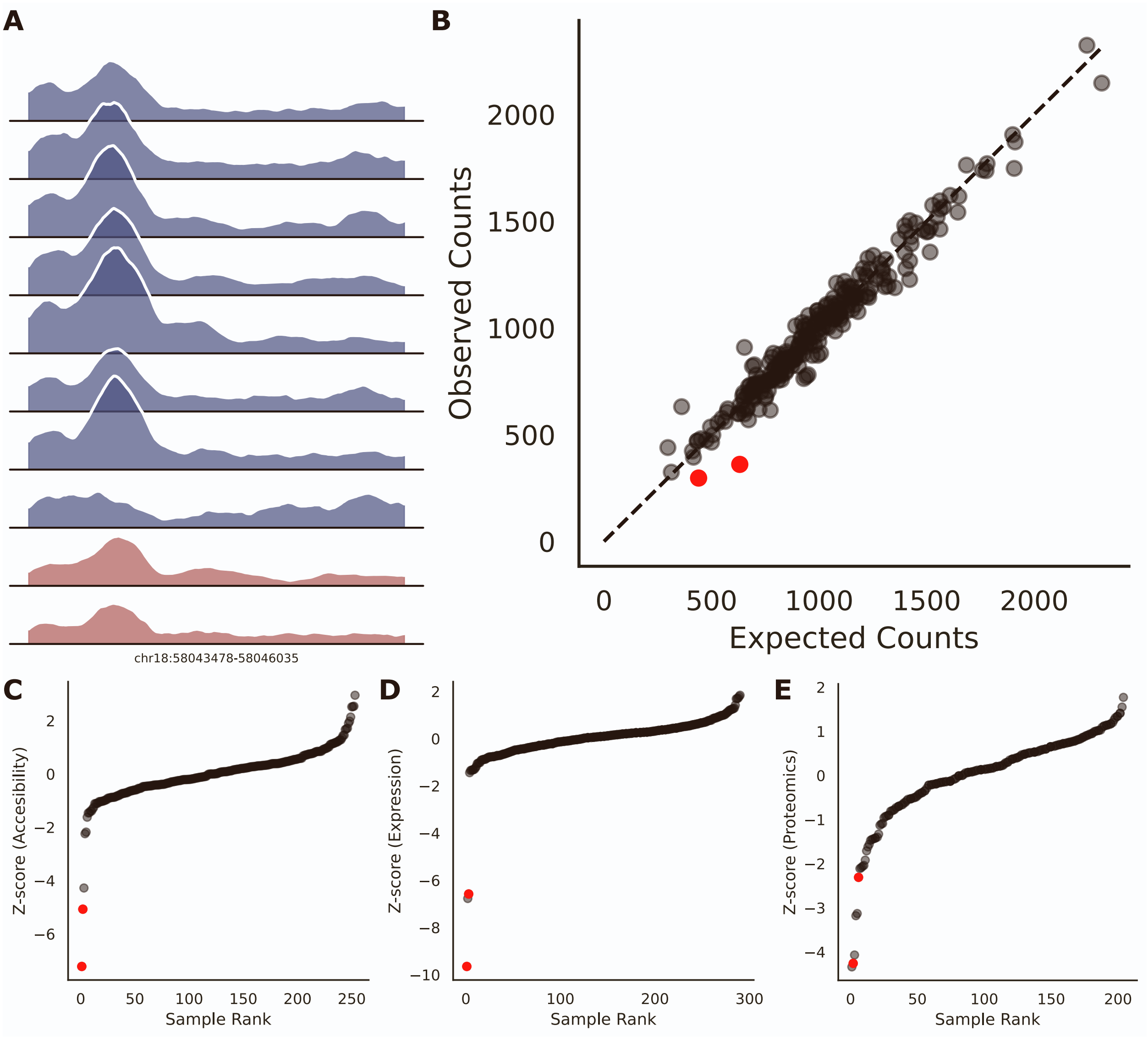

Figure S15. Aberrations observed at multiple omics levels of *NEDD4L* gene.
(A) ATAC-seq read coverage at the promoter of the gene. Samples with an outlier promoter are indicated by red across panels. **(B)** Expected and observed accessibility in the promoter of the gene across samples **(C)** Z-score distribution of promoter accessibility **(D)** gene expression **(E)** protein levels across samples.

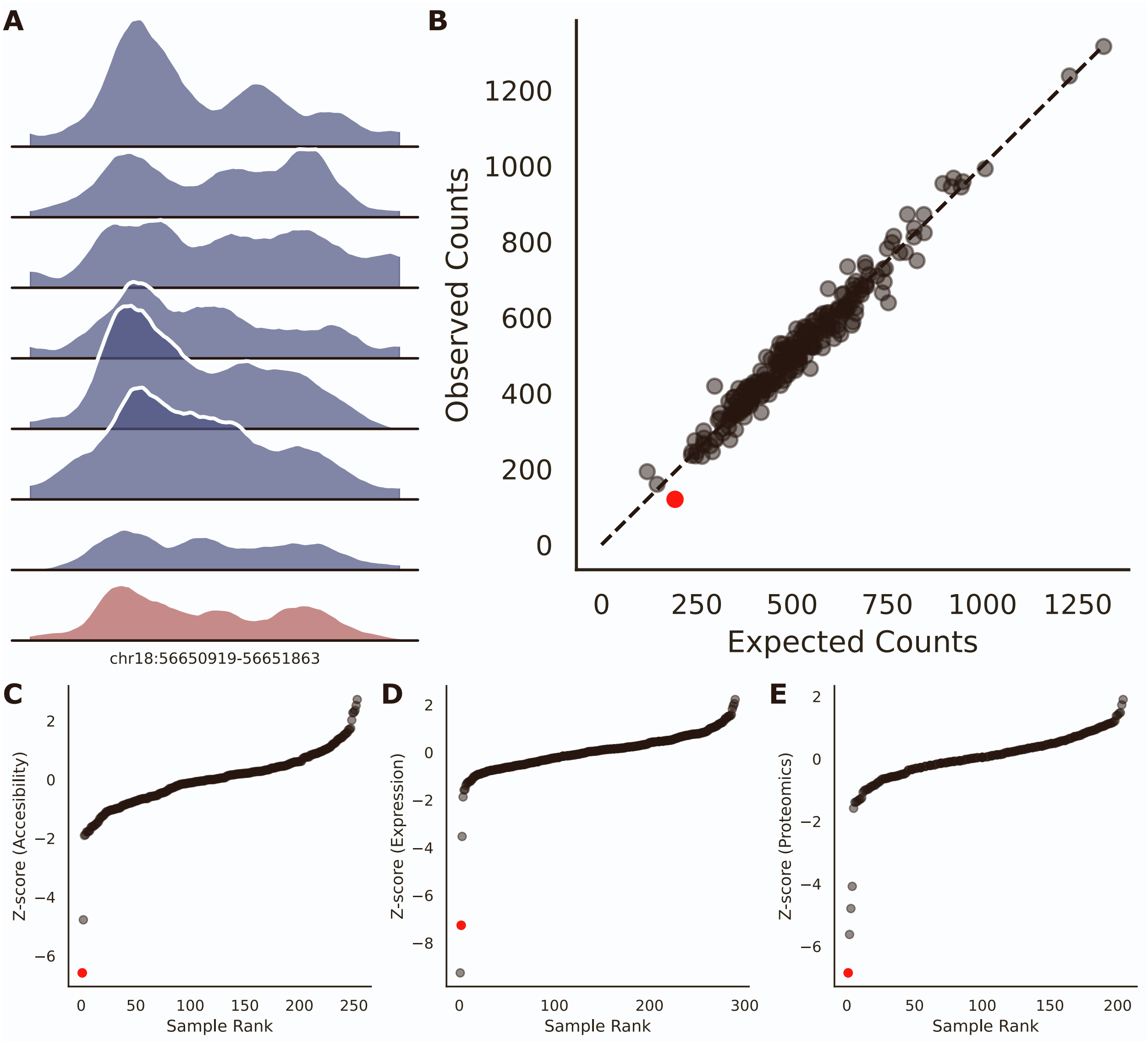

Figure S16. Aberrations observed at multiple omics levels of *TXNL1* gene.
(A) ATAC-seq read coverage at the promoter of the gene. The sample with an outlier promoter is indicated by red across panels. **(B)** Expected and observed accessibility in the promoter of the gene across samples **(C)** Z-score distribution of promoter accessibility **(D)** gene expression **(E)** protein levels across samples.

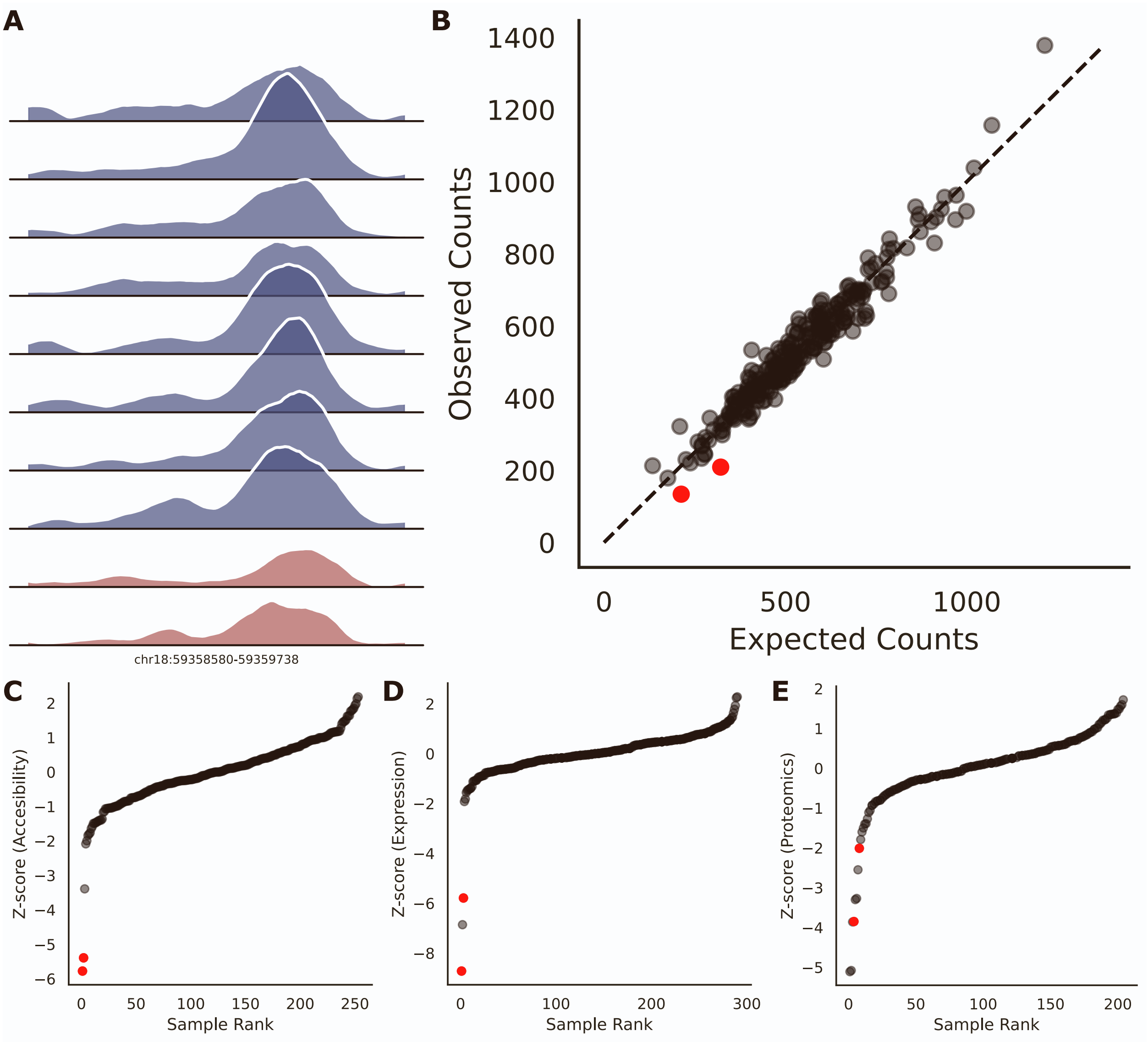

Figure S17. Aberrations observed at multiple omics levels of *LMAN1* gene.
(A) ATAC-seq read coverage at the promoter of the gene. Samples with an outlier promoter are indicated by red across panels. **(B)** Expected and observed accessibility in the promoter of the gene across samples **(C)** Z-score distribution of promoter accessibility **(D)** gene expression **(E)** protein levels across samples.

Table S1. (separate file)

List of chromatin accessibility outliers detected across the AnswerALS samples.

Algorithm S1

Algorithm 1 Read counting algorithm for accessible regions

Require: a bam file and non-overlapping *peaks* sorted by position
Ensure: Read *counts* as a dictionary

for each: $chrom \in chromosomes$
  $peaks \leftarrow$ create a stack by subsetting peaks in $chrom$
  $reads \leftarrow$ create a stack by fetching reads from bam file for $chrom$

  $read \leftarrow reads.dequeue()$
  $peak \leftarrow peaks.dequeue()$

  do
    if $read.end <= peak.start$ **then**
      $read \leftarrow reads.dequeue()$
    else if $read.start >= peak.end$ **then**
      $peak \leftarrow peaks.dequeue()$
      $counts[peak] \leftarrow 0$
    else
      $counts[peak] + +$
      $read \leftarrow reads.dequeue()$
    end if
  while peaks $\neq \emptyset$
