# Supplementary material for: Identifying dysregulated regions in amyotrophic lateral sclerosis through chromatin accessibility outliers
Source: HGG Adv. 2024 Jun 13;5(3):100318. doi: 10.1016/j.xhgg.2024.100318 (PMC11260578; doi:10.1016/j.xhgg.2024.100318)
Supplement: Document S2. Article plus supplemental information [file mmc3.pdf]

HGG Advances

ARTICLE

Identifying dysregulated regions in amyotrophic lateral sclerosis through chromatin accessibility outliers

Muhammed Hasan Çelik,[1,2] Julien Gagneur,[3,4,5,6] Ryan G. Lim,[7] Jie Wu,[8] Leslie M. Thompson,[7,8,9,10,11] and Xiaohui Xie[1,12,*]

Summary

The high heritability of amyotrophic lateral sclerosis (ALS) contrasts with its low molecular diagnosis rate post-genetic testing, pointing to potential undiscovered genetic factors. To aid the exploration of these factors, we introduced EpiOut, an algorithm to identify chromatin accessibility outliers that are regions exhibiting divergent accessibility from the population baseline in a single or few samples. Annotation of accessible regions with histone chromatin immunoprecipitation sequencing and Hi-C indicates that outliers are concentrated in functional loci, especially among promoters interacting with active enhancers. Across different omics levels, outliers are robustly replicated, and chromatin accessibility outliers are reliable predictors of gene expression outliers and aberrant protein levels. When promoter accessibility does not align with gene expression, our results indicate that molecular aberrations are more likely to be linked to post-transcriptional regulation rather than transcriptional regulation. Our findings demonstrate that the outlier detection paradigm can uncover dysregulated regions in rare diseases. EpiOut is available at github.com/uci-cbcl/EpiOut.

Introduction

Amyotrophic lateral sclerosis (ALS) is a rare neuromuscular degenerative disease affecting 0.6 to 3.8 per 100,000 people with a poor survival prognosis without a cure.[1,2] ALS is a complex disease where a single gene or pathway cannot explain the disease phenotype due to the heterogeneity of genetic causes and over 30 genes associated with ALS.[1,3] Meta-analysis and twin studies estimate the heritability of ALS disease at 61% (with 38%–78% confidence intervals) in sporadic cases (sALS), i.e., patients without a history of the disease in the family.[4] Despite the high heritability of ALS, only 11% to 25% of patients[5–7] receive a diagnosis after genetic testing. The gap between high heritability and low diagnostic rate implies the existence of many undiscovered ALS-related genes.

There are large-scale sequencing efforts to discover the genetic bases of ALS.[8,9] These studies utilized genome-wide association studies (GWASs), quantitative trait locus (QTL), and differential expression analysis from a large cohort of samples to detect aberrations in ALS patients compared with control samples.[8,10–12] These statistical approaches successfully detected the most common factors (variants, genes, and pathways) associated with disease phenotype, yet detecting rare genetic factors remains challenging due to low statistical power. Outlier detection is a complementary statistical approach that uncovers aberrations specific to one or a few patients. Applying the outlier detection paradigm to transcriptomics data has revealed the dysregulation of many novel splicing and gene expression outliers.[13–19] This approach offers a promising direction for enhancing molecular diagnostic rates of rare disorders, as it effectively captures their heterogeneous genetic architecture.

The outlier detection approach has recently been applied to proteomics[20,21] and methylation,[21] and robust replication of aberrations across multiple omics data demonstrates the reliability of the outliers for disease diagnostics. Expanding the outlier detection approach to chromatin accessibility could provide further insight into the dysregulation of functional regions and their impact on gene expression in disease. Because transcription factors typically bind to open chromatin regions, defining the activity of promoters and enhancers, which in turn regulate transcription.[22] Thus, aberrations in chromatin accessibility correlate with the dysregulation of gene expression potentially linked to diseases.[23–25] Despite the widespread use of assays such as assay for transposase-accessible chromatin with sequencing (ATAC-seq) and DNase-seq enabling genome-wide investigation of the chromatin accessibility landscape,[26] the outlier detection approach has not been applied to chromatin accessibility data to the best of our knowledge.

[1]Department of Computer Science, University of California Irvine, Irvine, CA, USA; [2]Center for Complex Biological Systems, University of California Irvine, Irvine, CA, USA; [3]Department of Informatics, Technical University of Munich, Garching, Germany; [4]Helmholtz Association - Munich School for Data Science (MUDS), Munich, Germany; [5]Institute of Human Genetics, School of Medicine, Technical University of Munich, Munich, Germany; [6]Institute of Computational Biology, Helmholtz Center Munich, Neuherberg, Germany; [7]Institute for Memory Impairments and Neurological Disorders, University of California Irvine, Irvine, CA 92697, USA; [8]Department of Biological Chemistry, University of California Irvine, Irvine, CA, USA; [9]UCI MIND, University of California Irvine, Irvine, CA, USA; [10]Department of Psychiatry and Human Behavior and Sue and Bill Gross Stem Cell Center, University of California Irvine, Irvine, CA, USA; [11]Department of Neurobiology and Behavior, University of California Irvine, Irvine, CA, USA
[12]Lead contact
*Correspondence: xhx@ics.uci.edu
https://doi.org/10.1016/j.xhgg.2024.100318.
© 2024 The Author(s). This is an open access article under the CC BY license (http://creativecommons.org/licenses/by/4.0/).

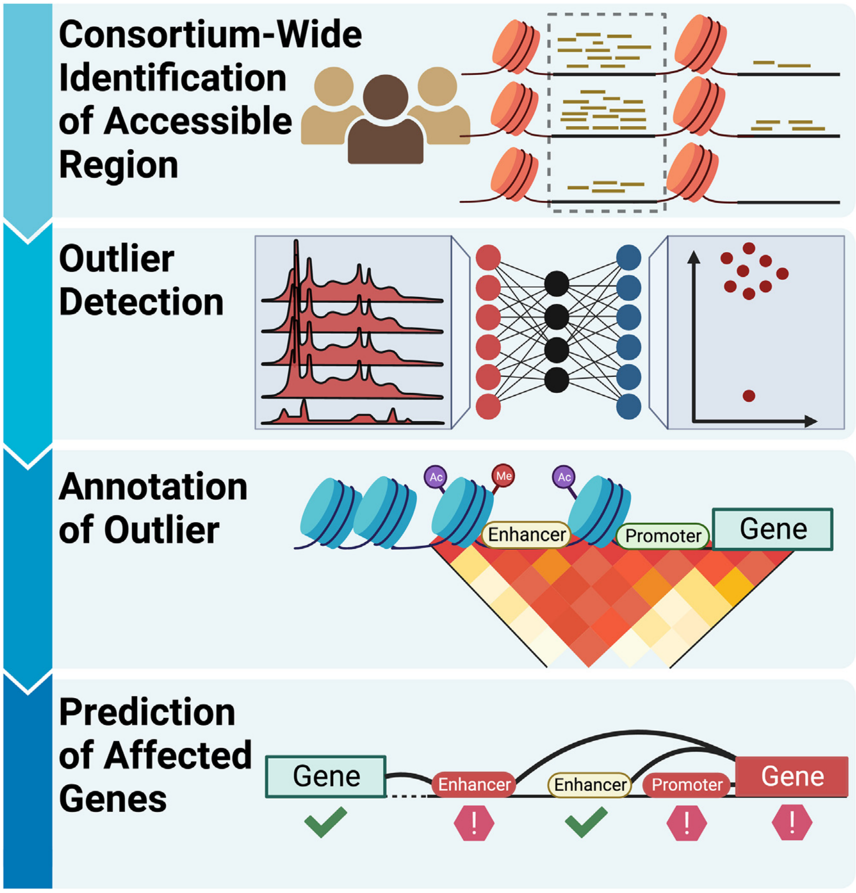

Figure 1. EpiOut offers a framework for the identification of dysregulated regions using chromatin accessibility data
First, a consistent set of accessible regions is identified across the cohort of samples, and sequencing reads from the assay are counted per region. Chromatin accessibility outliers are detected from the count matrix using a negative binomial test, with the expected counts calculated by an autoencoder. Then, the accessible regions are annotated to facilitate functional interpretation and to map interactions between regions. Finally, the potential impact of chromatin accessibility outliers on gene expression is predicted. These predictions help distinguish gene expression aberrations that arise from transcriptional or post-transcriptional regulation.

Here, we present EpiOut, a software developed for chromatin accessibility outlier detection (Figure 1). Our proposed method takes read alignment files and accessible regions as input, performs ultra-fast read counting per accessible region, detects outliers using a linear autoencoder (LR-AE) with a negative binomial objective function, and annotates outlier regions using chromatin immunoprecipitation sequencing (ChIP-seq) and Hi-C. Optimization of the decoder layer and dispersion parameters requires solving a large number of independent convex problems. We significantly accelerated the LR-AE using TensorFlow[27] by utilizing a vectorized variation of backtracking line search for the dispersion parameters and L-BFGS for the decoder layer. We utilized EpiOut to identify chromatin accessibility aberrations in motor neuron cells from 253 samples in the AnswerALS cohort,[9] which comprises multiple omics data from ALS patients and clinically healthy controls. EpiOut pinpoints a small number of sample-specific loci as outliers. Comparison of chromatin accessibility outliers with gene expression outliers and protein aberrations reveals consistent replication across multiple omics levels. This analysis can offer valuable insights into whether aberrations in molecular phenotype are influenced by transcriptional or post-transcriptional regulation. The outlier detection approach identifies known ALS genes and potentially novel disease gene candidates.

Results

In this study, we explored aberrant chromatin accessibility in ALS using ATAC-seq experiments from the AnswerALS dataset (methods). This dataset comprises paired genomics, transcriptomics, chromatin accessibility, and proteomics experiments from 253 individuals. With our novel method, EpiOut, we pinpointed regions with abnormal chromatin accessibility and investigated the molecular impact of these outliers by comparing paired experiments across different omics levels. Our results highlight the biological relevance and robustness of the identified accessibility outliers.

Detection of accessible regions

Detection of aberrant accessibility requires read counts for a set of accessible regions consistent across the individuals. First, we merged ATAC-seq reads of all samples into one meta-sample, then performed joint peak calling with MACS2[28] using the meta-sample to obtain a consistent set of accessible regions. Joint peak calling results in narrower regions compared with sample-specific peak calling, and there is a substantial overlap ($\sim$ 88% for under-accessibility and $\sim$ 63% for over-accessibility) in outliers identified by the two methods (Figures S1A and S1D). We detected a total of 858,268 peaks before any filtering. Next, we counted the number of ATAC-seq reads overlapping with each accessible region. Counting reads from a large sample cohort is computationally intensive.[15] Thus, we developed EpiCount, an efficient read counter designed for accessibility data. EpiCount is twice as fast as the state-of-the-art generic counting tool[29] (methods), using $\sim$ 50 times less memory (Figures S2A and S2B) and 15 times faster than the reported runtime of commonly used counting methods.[15] The runtime of EpiCount is

comparable with the bedtools[30]' implementation of the chrom-sweep algorithm.[31] The tidy memory footprint of EpiCount enables the independent parallelization of counting across a large number of samples. Last, we filtered accessible regions based on the read counts because many accessible regions often do not replicate across samples. We imposed a replication filter to these peaks, ensuring that the accessible regions were observed in at least 50% of the samples with a minimum of two reads and exhibited high accessibility (100 reads) in at least one sample (Figure S3A). Applying these filters yielded 114, 428 accessible regions replicated across samples.

Outlier detection and benchmark

Using an outlier detection approach, we aim to spotlight the rare aberrations unique to a few or single samples. This was achieved by eliminating major covariation in chromatin accessibility data and examining the remaining variance between samples. We investigated the relationship between the principal components of accessibility data and the disease status of the samples. Figure 2A illustrates the lack of clustering between samples by disease phenotype along the site of the top two principal components of chromatin regions with highly variable accessibility (counts are normalized with variance stabilizing transformation [VST][32]). The top 25 principal components account for approximately 79% of the chromatin accessibility variation between samples (Figure S4A). However, none of these top principal components significantly separate ALS samples from controls in this cohort (Figure S4B). Similarly, top principal components of transcriptomics data do not clearly separate cases from the control samples (Figures S5A and S5E). The observations align with the biology of ALS, given that the most prevalent cause, a hexanucleotide (GGGGCC) repeat expansion in the *C9orf72*, is present in only about 7% of patients, and other known factors account for merely 1%–2% of cases.[7] Thus, focusing on the rare aberrations might reveal dysregulation associated with ALS.

To evaluate the performance of the outlier detection methods, we employed an artificial outlier injection procedure previously proposed for detecting aberrant gene expression.[33] To create ground truth, we injected large aberrations, called artificial outliers, to read counts of ALS samples and then benchmarked the performance of tools to classify those artificial outliers on the area under the precision-recall curve (methods) (Figure 2C). In naive negative binomial, we estimated the mean of the negative binomial test as a sample mean of read count per ATAC-seq peaks (methods) and estimated dispersion with maximum likelihood estimation (MLE), then ranked predictions by p value based on the negative binomial test. The naive negative binomial model performs poorly with area under the precision-recall curve (auPRC) of $1.8\% \pm 1\%$ because the expected read counts of the naive negative binomial model are not sample specific. As an alternative outlier detection method, expected read counts per peak and sample can be estimated by principal-components analysis (PCA) (methods), and ranking predicted outliers by Z score based on the expected and observed read counts of the PCA model have the performance of auPRC $9.7\% \pm 0.9\%$. Our proposed method estimates expected read counts using an LR-AE (Figure 2B). Expected read counts are incorporated into a negative binomial test as the mean parameter, and the dispersion is estimated based on the expected and observed counts (methods). In our implementation of the LR-AE, the weights of the encoder and decoder layers are initialized with the rotation matrix of PCA. Then, the dispersion parameter is initially estimated using expected counts based on initial weights, and weights of the decoder layer are updated to maximize negative binomial likelihood using initial dispersion estimation. After the decoder layer optimization, we recalculate the dispersion estimation and apply the negative binomial test to estimate outliers. Also, it is critical to account for read coverage differences between samples (Figure S3B); thus, read counts are normalized for size factors[32] (methods). We chose the optimal bottleneck size of LR-AE with hyperparameter tuning on the validation set (Figure S6). This approach outperforms the previous two methods by achieving an auPRC of $50.3\% \pm 0.2\%$. An alternative LR-AE-based method, OUTRIDER, outperforms PCA and performs similarly with EpiOut (auPRC of $48\% \pm 0.2\%$). Both methods employ an LR-AE with a negative binomial, a more proper distribution to fit counts data than PCA (methods). Those results show that the estimation of dispersion and gene/sample-specific accessibility expectation followed by the negative binomial test is critical for outlier detection.

Chromatin accessibility is higher dimensional than gene expression because accessibility data may contain hundreds of thousands of accessible genomic regions. In contrast, gene expression data only contain around 10,000 to 15,000 expressed protein-coding genes. Thus, the scalability of the outlier detection approach is essential to apply the method to high-dimensional chromatin accessibility data. Although OUTRIDER and EpiOut have similar auPRC scores, the autoencoder implementation of OUTRIDER is significantly slower than our proposed method (Figure 2D). For example, outlier detection with EpiOut (745 ± 49 seconds) is 60 times faster than OUTRIDER ($44, 874 \pm 200$ seconds). Our implementation is faster due to a couple of reasons. First, we do not optimize the encoder layer of the autoencoder because we observed that the initial estimation of encoder weights with PCA is close to optimal, so further training of the encoder is unnecessary. Also, EpiOut only performs one alternating optimization step to fit the decoder layer and estimate dispersion. In contrast, OUTRIDER performs multiple alternative optimization steps to estimate dispersion and train the encoder and decoder layers. Last, the optimization of the decoder layer and the estimation of dispersion parameters necessitate solving a multitude of independent convex optimization problems. We efficiently approach these using a vectorized L-BFGS for the decoder

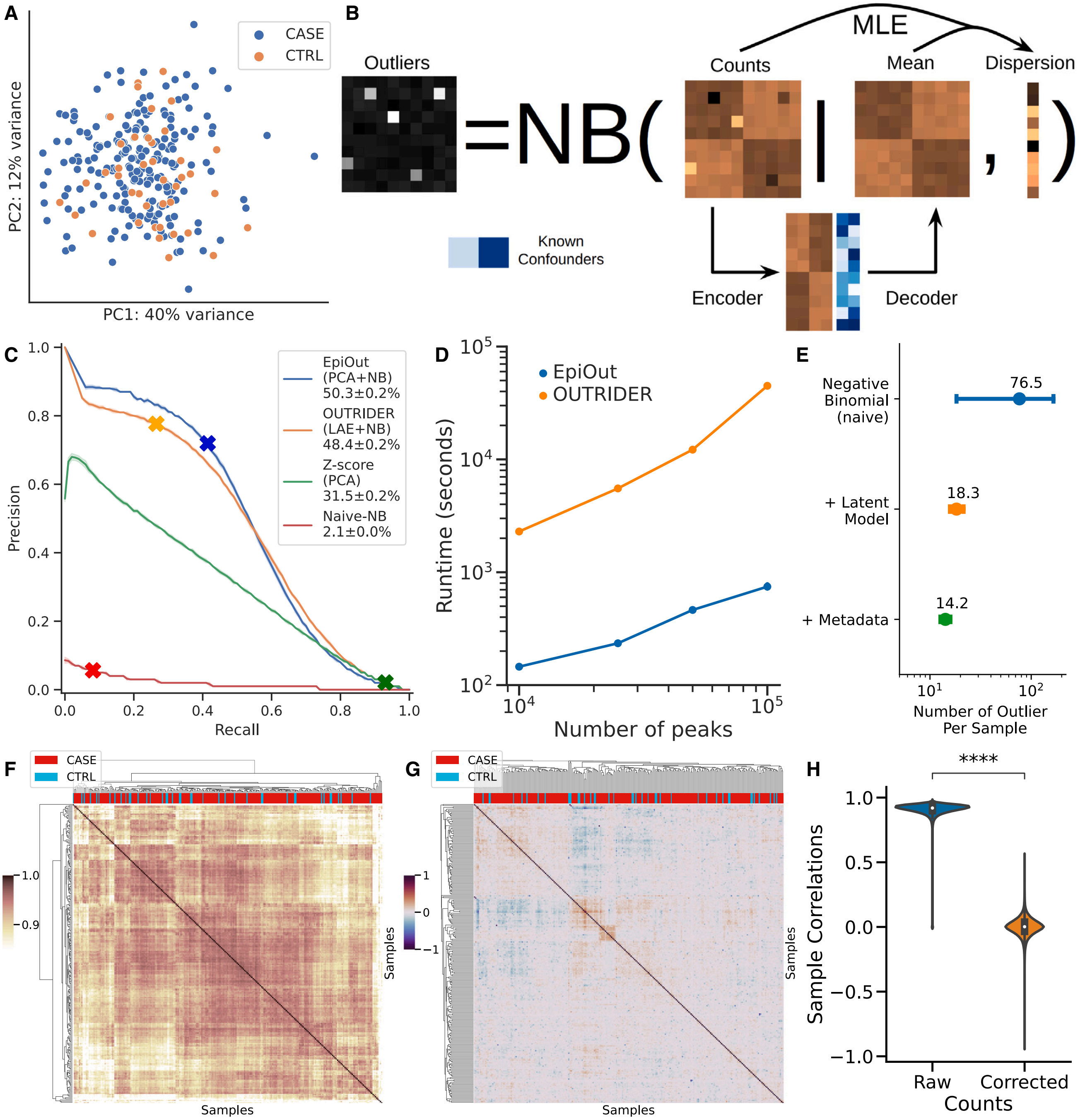

Figure 2. Proposed outlier detection methodology and its benchmark

(A) Samples do not form distinct clusters based on their disease phenotype according to the top two principal components of chromatin regions with highly variable accessibility (read counts are normalized for VST).

(B) The architecture of the proposed methodology for outlier detection (EpiOut). The approach employs the negative binomial test. The mean parameter of the negative binomial distribution is predicted with an LR-AE, which uses latent confounders obtained from data in addition to reported known confounding as features to predict sample-specific expected accessibility. The dispersion parameter is fitted with MLE using the observed and expected counts.

(C) A precision-recall curve shows the performance of alternative outlier detection methods. Methods were benchmarked based on the classification accuracy of the injected artificial outlier. Specific cutoffs of models are indicated with cross marks (an absolute Z score of 2 for PCA, a p value of 0.05 for naive negative binomial, OUTRIDER, and EpiOut).

(D) Runtime benchmark of outlier detection methods.

(E) Contribution of each component (such as latent and known confounding factors) of the model to reduce the number of outliers per sample (errors indicate standard deviation).

(F) A cluster heatmap displaying the cross-correlation of samples based on the read counts of regions with highly variable accessibility (normalized for VST) before controlling for cofounders and (G) cross-correlation of samples after correction of counts.

(H) Distribution of cross-correlation between sample pairs before and after correction of accessibility reads ($****p < 10^{-4}$).

layer and a vectorized and bounded backtracking line search for dispersion estimation, all implemented in TensorFlow (methods).

We further integrated known confounding factors such as batch ID, sex, reported race, and ethnicity into outlier detection (methods). The contribution of each feature for outlier detection is summarized in Figure 2E. The naive negative binomial test estimates 76.5 ± 614 outlier regions per sample, and the inclusion of latent confounding factors for expected read count estimation significantly reduces the number of outliers to 18.3 ± 24. Known confounding factors such as batch ID, sex, race, and ethnicity further reduce the number of an outlier to 14.2 ± 13.6 out of $114,428$ accessible regions per sample. Thus, our method pinpoints only a handful of aberrant regions per sample.

As another benchmark, we compared cross-correlation between samples. Samples are highly cross-correlated based on the read counts of regions with highly variable accessibility (normalized for VST) before controlling for cofounders (Figure 2F). In our detection approach, we aim to eliminate the correlation between samples to detect rare aberrations by controlling for latent cofounders. Correcting for latent and known confounding factors and normalization of read counts decorrelate samples and eliminate clusters due to potential batch effects (Figure 2G). Samples have an average cross-correlation of 90.9% based on the read counts before correction. In comparison, there is a 0% average cross-correlation after the proposed correction method ($p < 0.001$ based on the paired Wilcoxon test, Figure 2H).

Functional annotation of accessible regions and outliers

To aid the functional interpretation of chromatin accessibility outliers, we developed EpiAnnot, which annotates accessible regions for histone marks and 3D chromatin interactions based on ChIP-seq and Hi-C experiments. EpiAnnot integrates publicly available ChIP-seq and Hi-C for cell lines/tissues available in Roadmap Epigenomics[34] and ENCODE[35] or from custom data sources. We annotated the previously identified accessible regions and outliers using the H3K4me3, H3K27ac, and H3K4me1 histone marks observed in motor neurons. These neurons were derived from the iPSCs of clinically healthy individuals and ALS samples.[36] Based on histone marks, accessible regions were further classified as a promoter if the region has a histone mark of H3K4me3 and is within $1,000$ base pair (bp) vicinity of the annotated transcript start site or overlaps with $5'$ UTR, an active enhancer if the region has both H3K27ac and H3K4me1 mark, and a poised enhancer if only H3K4me1 signal is present while H3K27ac mark is lacking (Figure 3A).[37] The two largest categories of accessible regions were active enhancers ($n = 39,559$), which have both H3K27ac and H3K4me1, followed by poised enhancer ($n = 18,817$) regions, which have only the H3K4me1 mark (Figure 3B); 14.3% accessible regions ($n = 16,446$) have all three H3K4me3, H3K27ac, and H3K4me1 histone marks. Chromatin accessibility outliers are enriched for histone marks, for example 28% of outlier overlap with all three histone marks (Figure 3C). Consequently, both over-accessibility and under-accessibility outliers are more likely to occur in promoter regions (Figure 3D, $p < 0.001$ for both based on Fisher's exact test). Active enhancers are the largest category of outliers, contain 39% of over-accessibility and 38% of under-accessibility outliers, and not significantly enriched or depleted for outliers ($p = 0.26$ for under-accessibility and $p = 0.22$ for over-accessibility based on Fisher's exact test). Furthermore, both poised enhancer and unannotated regions strongly depleted for under-accessibility outliers as expected ($p < 0.001$ for both based on Fisher's exact test). Significant enrichment of outliers in functional regions indicates the potential utility of accessibility outliers in delineating molecular basis of ALS.

Another interesting observation is that outliers tended to occur in the vicinity of each other (Figure 3E). Specifically, 8% of outliers have a second outlier in the 10 kilo-bp vicinity with an odds ratio of 688 ($p < 0.001$ based on the Fisher's exact test), and 34% of outliers have a second outlier in 1 million bp with the odds ratio of 33 ($p < 0.001$ based on the Fisher's exact test). We repeated enrichment analysis between promoter outliers and active or poised enhancer outliers and again observed significant enrichment. Thirteen percent of promoter outliers have at least one active enhancer outlier in 100 kilo-bp vicinity (odds ratio $= 375, p < 0.001$ based on the Fisher's exact test), and 24% of promoter outliers have an active enhancer outlier in 1 million bp vicinity (odds ratio $= 70, p < 0.001$ based on the Fisher's exact test). The co-occurrence of the outliers indicates the potential interaction between them. Hi-C experiments from motor neurons provide further evidence for the potential interaction between outliers. For example, the relatively high Hi-C contact score between the outlier promoter of the *ZFP41* gene and a distal enhancer outlier located ~ 110 kilo-bp upstream of the gene suggests a potential interaction between outliers (Figure 3F). We calculated the Hi-C contact score between pairs of accessible regions and categorized regions by outlier status. We observed that outlier pairs ($p < 0.001$ based on the Mann-Whitney U test), including promoter-active enhancer pairs ($p < 0.001$ based on the Mann-Whitney U test), have higher interaction scores compared with a baseline where at least one of the regions in the pair is not an outlier (Figure 3G). Hi-C contact scores are distance-dependent and decay according to power law with increasing genomic distance. Thus, a higher interaction score could possibly be confounded by the co-occurrence of outliers in the vicinity of each other. To avoid this potential bias, we fit power regression on Hi-C contact scores using the outlier status as a feature and distance as a control variable (methods). Outlier pairs have higher interaction scores ($p < 0.001$ based on the t test) even after controlling for distance with power regression (Figure S7A). Moreover, even when we restricted our analysis to region pairs at

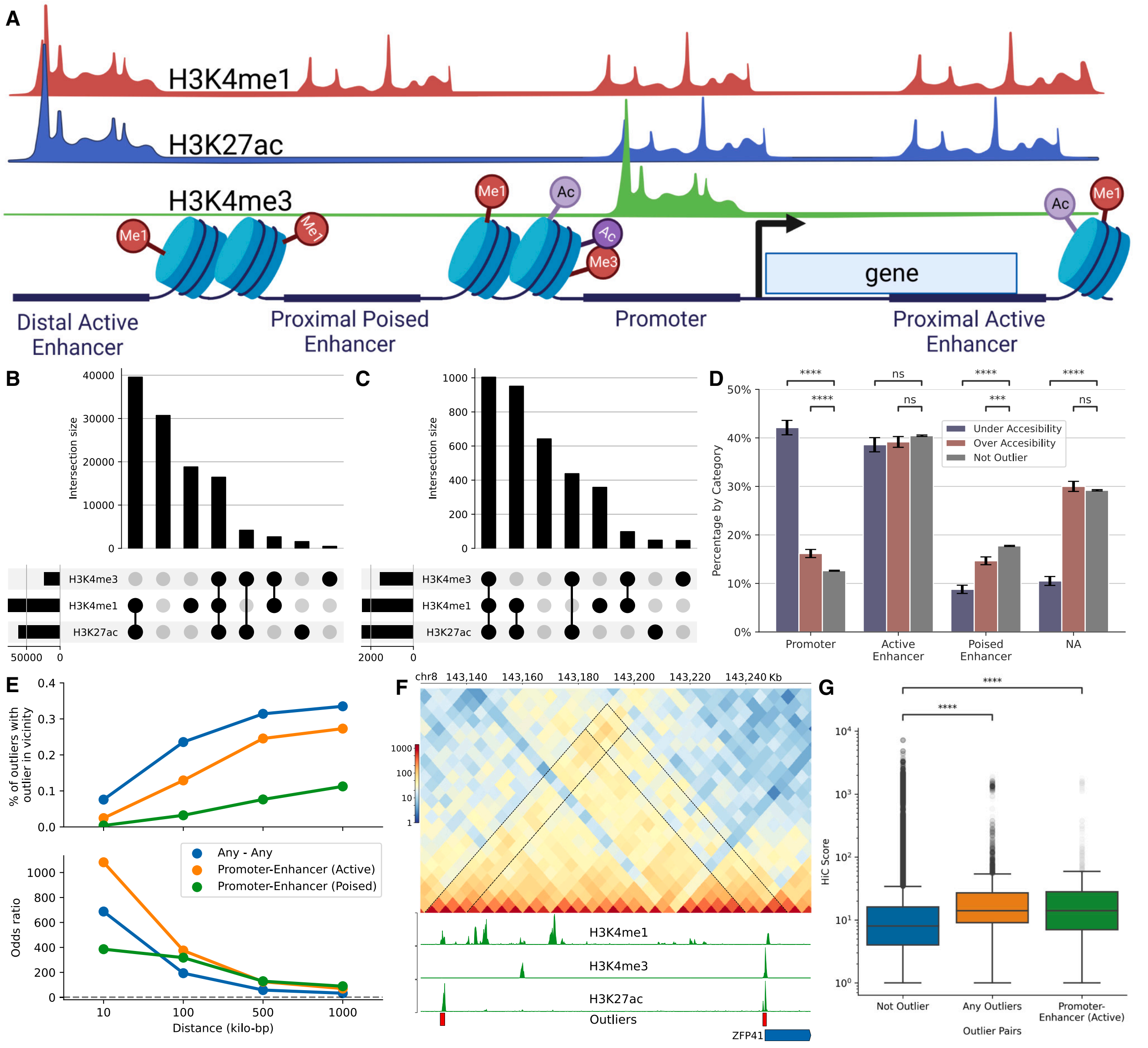

Figure 3. Functional annotation of chromatin accessibility outliers

(A) Accessible regions were annotated using ChIP-seq marks and gene annotation as promoters, active or poised, and proximal or distal enhancers.

(B) Overlap between H3K4me3, H3K27ac, and H3K4me1 histone marks and accessible regions (C) and outliers (D) Breakdown of promoters, active enhancers, poised enhancers, and unannotated regions within the categories of under-accessibility, over-accessibility outliers, and non-outliers. Errors bars indicate standard error, p values calculated with Fisher's exact test and corrected for multiple testing with the Benjamini/Hochberg method (ns ≥ 0.05, $***p < 10^{-3}$, $****p < 10^{-4}$).

(E) Cumulative odds of observing the second outlier in a region given that there is an outlier in the region (top) and the cumulative percentage of outliers with a second outlier in the vicinity (bottom) based on the distance between regions and the annotation.

(F) The interaction between the outlier promoter of *ZFP41* and ~ 100 kilo-bp apart outlier distal enhancer is highlighted by the Hi-C track containing contact score between 5 kilo-bp long genomic bins. Coverage tracks for H3K4me3, H3K27ac, and H3K4me1 histone marks are colored green. Red boxes indicate the outlier status of the accessible regions.

(G) The Hi-C contact scores distribution of non-outlier, outlier, and promoter-enhancer pairs. p values were calculated with the Mann-Whitney U test and corrected with the Bonferroni correction for multiple testing.

least 100,000 bp apart, the Hi-C contact scores of outlier pairs still surpassed the baseline of non-outlier pairs (Figure S7B). Overall, the co-occurrence of outliers in the vicinity of each other and higher Hi-C contact scores between outlier pairs indicate a potential interaction between outliers.

Chromatin accessibility outliers influence gene expression and protein levels

Aberration in accessibility can impact downstream molecular phenotypes such as gene expression and protein levels. As an example, we found that the ALS case with an under-accessibility outlier in the promoter of *LCMT1*

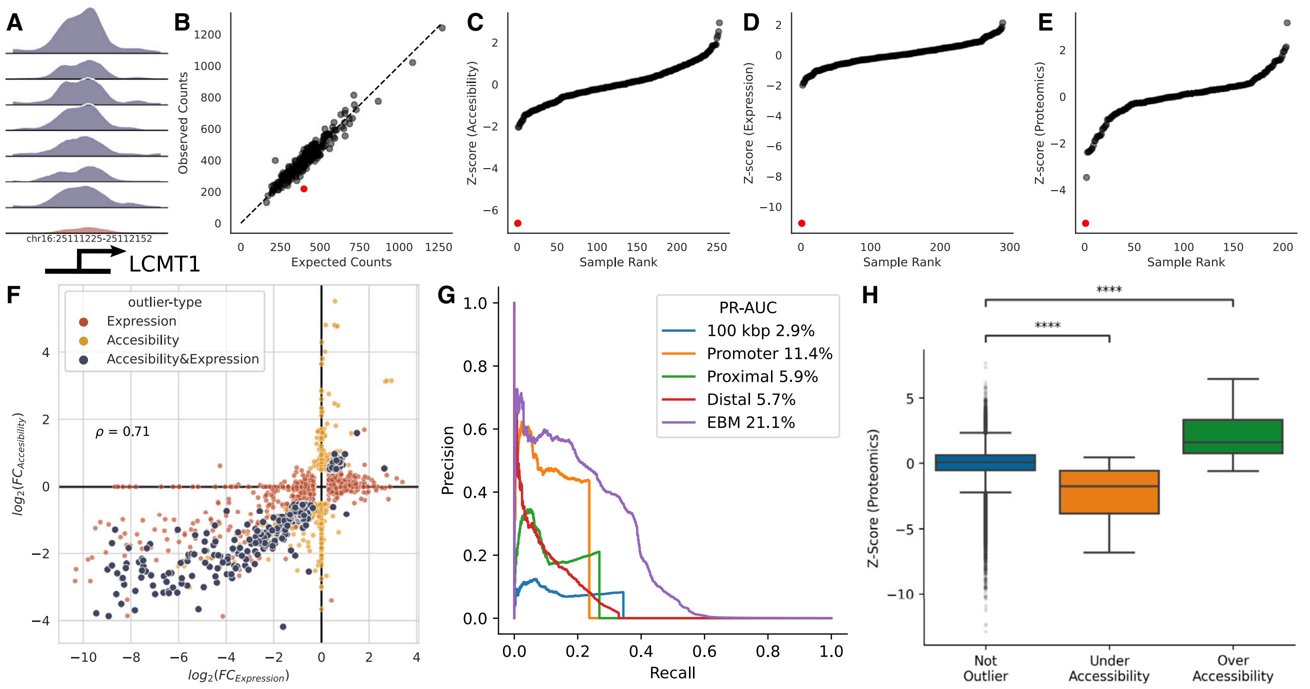

Figure 4. Prediction of gene expression outliers and aberrant protein levels from chromatin accessibility

(A) The outlier promoter of the *LCMT1* gene (in red) has a much lower ATAC-seq read coverage than promoters of non-outlier samples.
(B) Expected and observed accessibility in the promoter of LCMT1 gene across samples. The outlier sample is indicated with a red dot in the figure panels.
(C) *Z* score distribution of promoter accessibility, (D) gene expression, (E) and protein levels of *LCMT1* across samples.
(F) Correlation between the absolute log fold change of accessibility and expression outliers.
(G) The precision-recall curve compares the performance of a range of predictors to estimate gene expression outliers. Those predictors are the absolute log fold change of promoter outliers (orange), the absolute log fold change of proximal enhancers (green), the maximum absolute fold-change of any outlier in 100 kilo-bp vicinity (blue), the absolute log fold change of distal enhancers weighted by ABC score (red), explainable boosting machine (EBM) trained with promoter, proximal-enhancer, and distal-enhancer features (purple).
(H) *Z* score distribution of proteins categorized by the outlier status of the promoter that transcripts them ($****p < 10^{-4}$).

(Figures 4A–4C) also showed decreased mRNA (Figure 4D) and protein levels (Figure 4E).

To investigate the global relationship between accessibility outliers and gene expression, we compared the variations in promoter accessibility with variations in gene expression across all samples (Figure 4F). We observed a significant correlation between the fold changes ($log_2(FC)$) in accessibility at genes' promoters and the respective expression levels of these genes (Spearman's correlation coefficient = 71%, $p < 0.001$). The high correlation indicates that aberrations in promoter accessibility potentially influence the aberrations in gene expression.

Moreover, we demonstrated that gene expression outliers can be systematically predicted from the promoters, proximal, and distal enhancer accessibility (Figure 4G). Ranking promoter outliers by their absolute log fold change ($|\log_2(FC)|$) to predict gene expression outliers achieve an auPRC of 11.4%. If the promoter is an outlier, there is a 43.6% chance (precision) that its gene is an expression outlier, and 23.6% of gene expression outliers have an outlier promoter (recall). Similarly, outliers in proximity are highly predictive of gene expression outliers. Specifically, 26.8% (the recall at 21.1% precision) of gene expression outliers have at least one proximal outlier. Ranking genes based on the absolute log fold change of their proximal outlier achieves the performance of auPRC of 5.9%. We also ranked genes based on the maximum absolute fold-change of accessibility outliers in 100 kilo-bp vicinity regardless of annotation of outliers and achieved 2.9% auPRC. We weighted the absolute log fold change of distal outliers by ABC score[38] and obtained a score for each gene (methods). The score calculated from distal outliers is also a reliable predictor of gene expression outliers and achieves 5.7% auPRC. We trained an explainable boosting machine[39] to predict gene expression outliers by combining features from transcript start site, proximal, and distal outliers (methods). The machine learning model achieved 21.1% auPRC. Further benchmarking of alternative models[40] incorporating ChIP-seq annotations shows performance comparable to the model solely using GENCODE annotation (Figures S8A and S8B). Additionally, Hi-C measurements necessary for the ABC score can be approximated through power-law regression when unavailable (Figure S8C). Altogether, these results show that chromatin accessibility outliers are predictive for gene expression outliers, and chromatin accessibility aberrations in transcript start site, proximal, or distal regions often translate into gene expression aberrations.

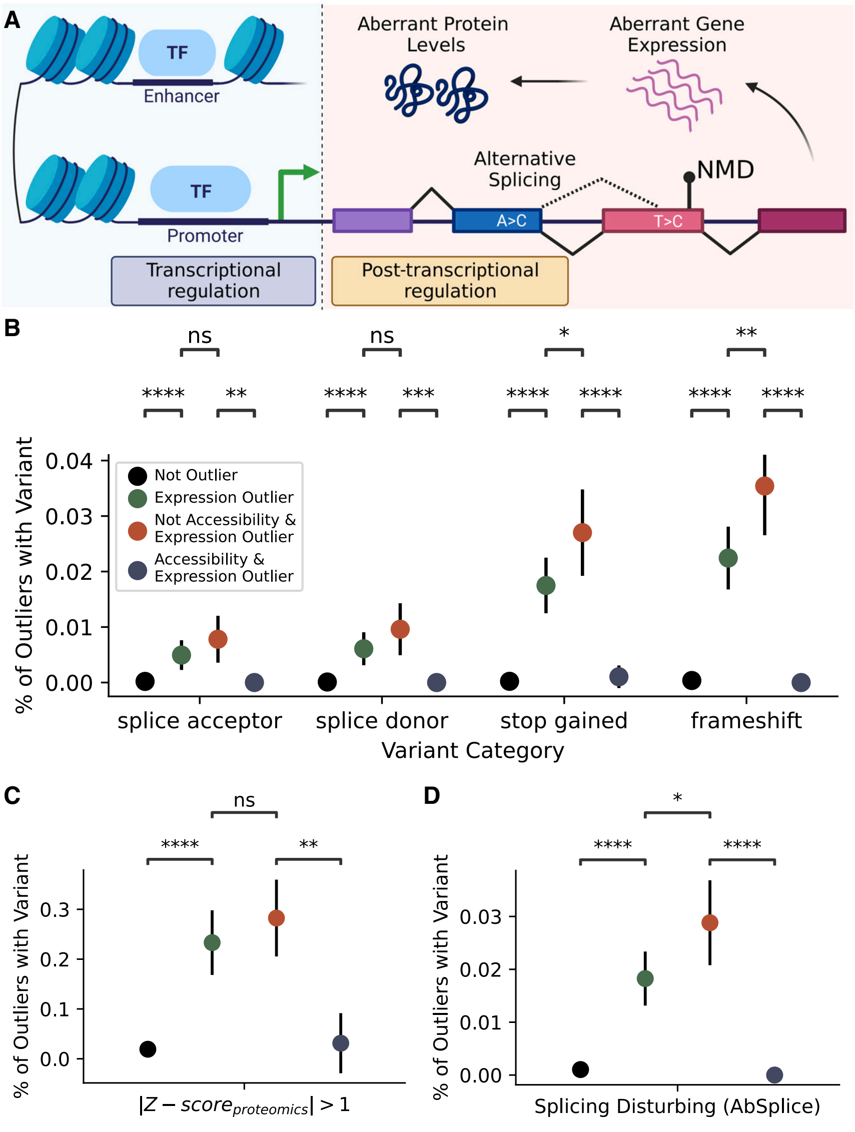

Figure 5. Overlap between accessibility outliers, gene expression outliers, and rare genetic variants by consequence
(A) Aberration in gene expression can result from dysregulation in transcriptional regulation, such as aberrant promoter or enhancer activity, or dysregulation in post-transcriptional regulation, such as splicing or nonsense-mediated decay. Error bars represent the 95% binomial proportion confidence intervals (Cis) in all panels.
(B) Percentage of genes with potentially NMD-triggering rare genetic variants by outlier categories. Gene expression outliers with promoter outliers are significantly depleted for variants in each category, while gene expression outliers without outlier promoters are enriched for nonsense and frameshift variants.
(C) Percentage of protein with a missense or potentially NMD-triggering rare variants where protein levels are at least deviant by $|Z \text{ score}| > 1$ by outlier categories.
(D) Percentage of genes containing splicing-disrupting variants predicted by AbSplice by outlier categories ($\text{ns} \geq 0.05, *p < 0.05, **p < 10^{-2}, ***p < 10^{-3}, ****p < 10^{-4}$ based on the hypergeometric test).

We focused on gene expression outliers with outlier promoters and investigated aberrations in their protein levels (Figure 4H). We observed that the gene expression outliers with overly accessible promoters have higher protein levels (average Z score $= 2.2, p < 0.001$ based on the Mann-Whitney U test, $n = 29$), and genes with under-accessible promoters have lower protein levels (average Z score $= 2.2, p < 0.001$ based on the Mann-Whitney U test, $n = 79$). Overall, we present the biological significance of accessibility outliers on molecular phenotype by replicating outliers from multiple omics levels.

A comparison of chromatin accessibility and gene expression reveals whether aberrations in molecular phenotype are linked to transcriptional or post-transcriptional regulation

The interplay between transcription and degradation rates determines RNA levels (Figure 5A). Aberrant promoter or enhancer activity can lead to up- or down-regulation of gene expression by altering transcription. Alternatively, alteration of post-transcriptional processes may affect RNA degradation, notably but not exclusively via nonsense-mediated decay (NMD).[16] Hence, the potential regulatory process associated with aberrant gene expression can be revealed by comparing accessibility outliers against gene expression outliers.

To demonstrate this point, we investigated the enrichment of rare and potentially NMD-triggering variants in different outlier categories. Figure 5B presents the frequency of potentially NMD-triggering rare variants, such as splice acceptor, donor, nonsense, and frameshift variants, based on their SnpEff[41] consequences by the outlier type of the affected gene. These variants were observed rarely and appeared in fewer than 0.1% of non-outlier genes. Compared with expression outliers with outlier promoter, these variants are significantly more prevalent in gene expression outliers if their promoters are not accessibility outliers. The enrichment holds for splicing acceptor ($n = 13, p = 0.0026$ based on the hypergeometric test) and splicing donor ($n = 16$, $p = 0.0007$), nonsense ($n = 46$, $p < 0.001$), and frameshift variants ($n = 59$, $p < 0.001$). Remarkably, only one of the gene expression outliers with aberrant promoter activity contains potentially NMD-triggering rare variants, indicating a significant depletion pattern (Figure S9A). Based on a subsequent analysis focusing on genes intolerant to loss-of-function (LoF) mutations[42] (with an LoF observed/expected upper bound fraction [LOEUF] of less than 35%), we observed a

similar pattern of depletion of potentially NMD-triggering variants among expression outliers with aberrant promoter accessibility ($n = 13, p = 0.0026$, Figure S9B).

Further investigation of both missense and potentially NMD-triggering rare variants in genes with aberrant promoter accessibility and protein levels presents a similar trend of depletion (Figure 5C); 19.8% of expression outlier genes ($n = 163, p < 0.001$ based on the hypergeometric test) with aberrant protein levels ($|\text{Z score}| > 1$) contain at least one of such variants. When genes with promoter outliers were excluded, the enrichment of variants rose to 24.1% ($n = 131, p = 0.27$). The remaining expression outlier genes ($n = 32$) have promoters with aberrant accessibility, and only one of these genes contains genetic variants with mentioned consequences. The substantial depletion of these variants in these genes ($p = 0.0052$) indicates that their aberrant protein levels are potentially linked to aberrant promoter accessibility rather than coding variants (Figure S9C).

Aberrant splicing is another mechanism that can affect gene expression by resulting in aberrant RNA isoforms subject to NMD.[43] Thus, we further explored the impact of exonic or intronic splicing-disrupting variants prioritized by AbSplice.[44] We detected 48 gene expression outliers containing at least one splicing-disrupting variant prioritized by AbSplice and none of these genes has an outlier promoter (Figure 5D). In contrast, subsetting gene expression outliers without aberrant promoters increases the prevalence of splicing disturbing variants for the subset (Figure S9D).

Furthermore, our investigation of genetic variants in chromatin accessibility outliers, which could disrupt transcription by altering *cis*-regulatory elements, showed slight enrichment of single-nucleotide variants (SNVs) or insertion-deletion mutations (indels) (Figure S10A, $n = 163, p < 0.001$ based on hypergeometric test). Structural variants are more frequently observed in the vicinity of chromatin accessibility outliers than in non-outliers; however, nearby structural variants are only identified in $\sim 1.3\%$ of these outliers (Figure S10B).

The results underscore that comparing accessibility outliers against gene expression outliers and aberrant protein levels can identify whether aberration in the molecular phenotype is tied to transcriptional or post-transcriptional regulation. The observed enrichment of small and structural variants in chromatin accessibility outliers might indicate that disrupting regulatory sequences within these accessible regions could lead to transcriptional aberrations.

Identifying known and suspected ALS genes using multi-omics outliers

We reviewed the literature to understand the biological significance of outliers observed across multiple omics levels in ALS samples. The proposed method operates in an unsupervised manner; consequently, not all detected aberrations are necessarily associated with ALS. For example, we observed a similar number of outliers in ALS cases and clinically healthy controls (Figures S11A and S11B). Furthermore, loci prioritized through outlier analysis are orthogonal to those identified by GWASs, as the proposed methodology captures rarer effects that may not be detected by GWASs (Figures S12A and S12E). Therefore, the integration of additional evidence from the literature is crucial to prioritize genes potentially linked to ALS pathogenesis. Among the outlier genes that exhibited aberrations in both promoter accessibility and gene expression (Table S1), 12 have previously been associated with ALS: *CDKL5*, *HIF1A*, *ABCA2*, *VPS4B*, *NOVA1*, *NRG1*, *NIPA1*, *BCL2*, *ALYREF*, *UBQLN2*, *IRAK4*, and *DDX3X*.[1,5,7,11,45–51] In some cases, variants have been associated with ALS or pathways involving these genes are dysregulated. While several proteomics measurements were missing due to the limitations of mass spectrometry,[52] three genes from these outliers (*VPS4B*, *ALYREF*, *DDX3X*) also displayed aberrant protein levels. For instance, we observed elevated expression and protein levels of *ALYREF*, in accordance with prior research (Figure S13) and knocking down an orthologue of *ALYREF* in an animal model reduces *TDP-43* induced toxicity.[53] Similarly, the *CDKL5* gene exhibits over-expression with an over-accessible promoter region (protein levels are unavailable). Suppressing *CDKL5* expression using a small molecule probe enhances the survival of human motor neurons under endoplasmic reticulum stress conditions.[54] Another outlier gene we identified, *HIF1A*, contributes to motor neuron degeneration through hypoxic stress, and prolonged survival observed in ALS mice suggests up-regulation of *HIF1A* as a potential therapeutic target.[55] Finally, *VPS4B* is pathologically increased in familial and sporadic ALS neuronal nuclei.[56] A closer examination of these identified outlier genes could reflect potential mechanisms involved in ALS and/or illuminate pathogenesis in subsets of ALS patients.

While some of the outlier genes are previously unreported as being associated with ALS, they play an important role in pathways involved in ALS; thus, they might be linked to ALS pathogenesis. For example, the promoter of *LCMT1* is less accessible, and both its gene expression and protein level are down-regulated in our dataset. Increased tau phosphorylation has been reported in ALS[57] and down-regulation of *LCMT1*, in conjunction with the up-regulation of *HIF1A*, has been linked to tau hyperphosphorylation.[58] *DDX6* is another gene that is down-regulated across three omics levels (Figure S14) and is an LoF intolerant gene[42] (with an LoF observed/expected upper bound fraction of LOEUF = 17%). Although the role of *DDX6* in ALS has not been documented, *DDX6* plays a critical role in RNA metabolism, particularly in the assembly of stress granules, a pathway dysregulated in ALS.[59] Furthermore, *DDX6* interacts with the ALS gene *ATXN2*, and a knockout of *DDX6* severely disrupts p-body formation.[60] In two ALS samples, we observed reduced promoter accessibility, gene expression, and protein levels of *NEDD4L* (Figure S15), another LoF intolerant gene (LOEUF = 20%). *NEDD4L* is a direct substrate of *USP7*

that regulates proteotoxicity in ALS.[61] Oxidative stress is implicated in neurodegeneration,[62] and up-regulation of *TXNL1* has been shown to reduce oxidative stress in neurological conditions.[63,64] Therefore, the pronounced down-regulation of promoter accessibility, gene expression, and protein levels of *TXNL1* observed in our study may be associated with increased oxidative stress (Figure S16). Vesicle transport is dysregulated by LoF of ALS-associated genes, such as *VAPB*[65] or *NEK1*.[66] *LMAN1*, a cargo receptor for the endoplasmic reticulum-Golgi transport,[67] is also involved in the trafficking of neuroreceptors.[68] The observed reduction in promoter accessibility, gene expression, and protein levels of *LMAN1* in two of our samples could be of relevance to ALS (Figure S17). While the systematic identification of aberrations across multi-omics data serves as a foundation for formulating new hypotheses, establishing a definitive link between these prioritized genes and ALS pathogenesis requires further experimental validation.

By cross-referencing genes with those associated with neurodegenerative disorders in OMIM,[69] we obtained six additional gene expression outliers (*GAN*, *EIF4A2*, *NARS1*, *HSD17B10*, *ERCC8*, *SLC25A46*) with aberrant promoter accessibility that are involved in a range of neurodegenerative and neurodevelopmental disorders. A notable example is *ERCC8*, involved in DNA damage repair and when mutated causes Cockayne Syndrome,[70] an early-onset degenerative condition.[71] *ERCC8* has also been identified as a comorbid factor in shared genetics between Parkinson disease and ALS.[72]

Overall, our multi-omics level analysis both detects known ALS genes and introduces potential novel candidates that might be playing a role in the ALS disease pathways. The results demonstrate the utility of the proposed statistical approach for prioritizing suspected regions and generating hypotheses about ALS pathogenesis.

Discussion

In this study, we introduced EpiOut, a computational toolbox for detecting and annotating chromatin accessibility outliers, which are characterized as large aberrations in a few regions specific to a single or few samples. We applied our proposed method to ATAC-seq data from ALS patients and clinically healthy individuals. Our methodology employs a negative binomial test for detecting outliers with statistical significance. The mean parameter of the negative binomial is fitted using a linear autoencoder (LR-AE), and the dispersion parameter is inferred based on observed and expected counts using MLE. Controlling for both known and latent confounding factors is crucial to exclude outliers resulting from technical artifacts or confounding factors during outlier detection.[14,33] The proposed LR-AE is an effective statistical method for obtaining latent confounders from high-dimensional data. PCA, a specific case of an LR-AE, can also detect and correct for latent confounders better than alternative statistical methods.[73] However, PCA minimizes the Euclidean distance between measured and reconstructed counts; thus, it is suboptimal for discrete read counts of omics data with high dispersion and uncertainty due to low coverage.[74] Thus, the negative binomial objective in our methodology is more apt for discrete read counts. Moreover, our software is more scalable than OUTRIDER and optimized for chromatin accessibility with a dimensionality of hundreds of thousands of genomic regions. While primarily designed for ATAC-seq, our toolbox is readily compatible with other accessibility assays, such as DNase-seq. The scalable outlier detection backend also enables the further expansion of outlier detection into other high-dimensional omics modalities, such as DNA methylation.

Our toolbox includes EpiAnnot, which annotates accessible regions as promoters and enhancers based on ChIP-seq marks. It also establishes a link between enhancer and promoter pairs through the ABC score, which is derived from either predicted or observed Hi-C scores. Using EpiAnnot, we found that outliers are enriched in functional regions, particularly promoters and active enhancers. Interestingly, outlier pairs tend to occur nearby, with many promoter outliers tied to active enhancer outliers within 1 million bp vicinity. This observation is supported by the relatively high Hi-C contact scores for outlier pairs in the vicinity, indicating potential interactions between these outliers.

By examining multiple omics levels, we found consistent replication of outliers. Accessibility outliers are associated with downstream biological processes such as gene expression and protein levels. In particular, a significant proportion of the gene expression outliers can be predicted from the aberrant accessibility of the promoter, proximal, and distal enhancer regions. Similarly, aberrant promoter activity is correlated with up- and down-regulation of protein levels.

Analyzing the interplay between accessibility and gene expression outliers yields insight into whether aberration in gene expression originates from transcriptional regulation, such as increased synthesis rate via higher promoter activity, or post-transcriptional regulation, such as splicing or nonsense-mediated decay. We observed substantial depletion of NMD-triggering rare variants in gene expression outliers if promoters of these genes are an accessibility outlier and conversely observed enrichment of these variants if their promoter is not an outlier.

The outlier detection method presented here is subject to certain limitations. The methodology is effective at detecting rare aberrations in the molecular phenotype; however, it does not establish a definitive link between these aberrations and specific phenotypic traits. Integrating the outliers with additional evidence, such as known disease genes, is necessary for the effective prioritization of aberrations that might be linked to a disease. QTL analysis detects variants affecting molecular traits, yet prioritization of causal gene-disease links and estimation of disease risk requires the integration of GWASs with QTL in Mendelian randomization studies.[75] Similar integration of GWAS variants with outliers is challenging, given variants associated with outliers have low allele frequency; thus, they may not

be prioritized by GWASs. Additional methodological innovations are needed to establish an unbiased link between outliers and disease traits.

Another future research direction is the investigation of genetic factors leading to chromatin accessibility outliers. The enrichment of genetic variants in the chromatin accessibility outliers indicates that *cis*-regulatory element disrupting genetic variants might be one source of the aberrations. However, the majority of these variants are non-coding variants; thus, effective prioritization of specific variants leading to chromatin outliers requires the development of statistical tools such as sequence-based deep learning models.[76]

Our outlier detection methodology offers a novel avenue for studying rare diseases. We have successfully adapted the outlier detection approach to ATAC-seq data and underscored that chromatin accessibility is a beneficial complementary assay for rare disease diagnostics. Detected outliers in ALS samples are highly robust and consistently replicated across multiple omics levels. Many of these outliers are either known ALS genes or are involved in pathways implicated in ALS. Thus, the continued development and integration of the outlier detection approach with disease gene discovery methodologies may ultimately lead to a more comprehensive understanding of the genetic factors contributing to ALS. Such advancements could finally bridge the gap between the disease heritability and the known catalog of ALS disease genes.

Methods

AnswerALS dataset

The multi-omics dataset, which includes ATAC-seq, RNA-seq, proteomics, and whole-genome sequencing (WGS), for ALS was downloaded from the Answer ALS portal (dataportal.answerals.org). The data contain 245 individuals diagnosed with ALS and 45 samples from clinically healthy controls. All samples had corresponding ATAC-seq and RNA-seq experiments. A total of 253 samples also have WGS data. For the scope of our study, we restricted our analysis to the 253 samples that had paired data across three omics levels. Additionally, 204 of these samples have proteomics data. In the proteomics-related analysis, we subset and only used these samples with the proteomics data.

Peak calling

We performed joint peak calling on ATAC-seq data to detect accessible regions across all samples using MACS2.[28] First, we merged bam files from every sample into a unified bam file utilizing SAMtools,[77] and subsequently filtered out reads with a mapping quality (MAPQ) below 10. Duplicate reads were retained after merging reads across samples. The default arguments of MACS2 were used, except for the duplicate read filter. ATAC-seq peaks contained in the narrow peak bed file generated by MACS2 were used in the downstream analysis.

Read counting

We implemented an ultra-fast read counting algorithm for ATAC-seq described in Algorithm S1. The algorithm is a simplified version of the chrom-sweep algorithm.[31] The counting algorithm is optimized based on two primary assumptions: ATAC-seq peaks are not overlapping and are separated by at least a gap longer than a read length; moreover, both peaks and reads are sorted by the genomic coordinates. We ensure the first assumption by jointly calling peaks as described above and collapsing any peaks closer to each other than the minimum gap distance (default 200 bp); thus, an ATAC-seq read never intersects with two peaks simultaneously. Since the bam file format is pre-sorted by genomic coordinates, ATAC-seq peaks are sorted to guarantee the second condition before the counting step. The counting algorithm creates two stacks of sorted peaks and reads, iterates over reads and peaks, and tracks the number of overlaps (Algorithm S1). The resulting runtime complexity of the counting step is $O(r+p)$ where r is the number of reads, and p is the number of peaks. In the preprocessing step, peaks are sorted, so the overall complexity is $O(p\,log(p)+r)$. However, since the number of reads is much larger than the number of peaks (r > p), the algorithm practically behaves in linear runtime in terms of the number of reads. The memory complexity of counting is linear in terms of the number of peaks ($O(p)$) because reads are fetched iteratively per chromosome by leveraging the indexable file format of BAM.

Filtering peaks by replication rate

We applied two filters to the ATAC-seq peaks detected by MACS2 to ensure consistent peak replication across samples. First, we eliminated peaks with low coverage, specifically those with fewer than 100 reads in any samples. This cutoff could be adjusted based on the coverage of the dataset; however, outliers may not be identified for these peaks if their read coverage is too shallow. As a subsequent criterion, any peak must be supported by a minimum of two reads in at least 50% of all samples.

Size factor normalization

To account for coverage differences across the ATAC-seq experiments, we performed size factor normalization on the read counts, a method initially proposed by DESeq2.[32] The size factor s_i for a sample i is defined as:

$$k_i^g = \left(\prod_{j=1}^{n} k_{ij}\right)^{1/n} \quad \text{(Equation 1)}$$

$$s_j = \text{median}\frac{k_{ij}}{k_i^g} \quad \text{(Equation 2)}$$

where k_{ij} represents the number of reads mapped to region j in sample i and k_i^g is the geometric mean of the reads across regions for sample i. The median of k_{ij} to k_i^g ratios is defined as the size factor s_i of sample i.

Outlier detection

After normalizing the read counts of peaks with size factor normalization, we log-transformed the normalized counts and centered them around zero by subtracting the mean normalized counts:

$$x_{ij} = n_{ij} - \overline{n}_{\mathrm{j}} \quad \text{(Equation 3)}$$

$$n_{ij} = \log\left(\frac{k_{ij}+1}{s_j}\right) \quad \text{(Equation 4)}$$

Autoencoder is applied on x_{ij} to calculate the expectation of normalized read counts ($\widehat{x}_{ij}$). f_e is the encoder function of the autoencoder, which takes observed normalized counts (x_{ij}) as input

and calculates major covariates. Major covariates are concatenated with known confounders to obtain latent representation (h), which is decoded back to expected normalized counts ($\bar{x}_{ij}$) with the decoder function f_d:

$$\mathrm{h} = \left[f_e(x_{ij})\middle|c_{known}\right] \quad \text{(Equation 5)}$$

$$\widehat{x}_{ij} = f_d(h) \quad \text{(Equation 6)}$$

We used linear encoder and decoder functions. The encoder model uses a principal-component analysis (PCA), where the encoder weights (W_e) are the rotation matrix of PCA. The decoder weights (W_d) are initialized with linear regression and further trained with the negative binomial objective:

$$f_e(x) = W_e\mathrm{x} \quad \text{(Equation 7)}$$

$$f_d(h) = W_d\mathrm{h} \quad \text{(Equation 8)}$$

The log-normalized counts (x_{ij}) are transformed back to the original natural scale:

$$\widehat{k}_{ij} = e^{\hat{x}_{ij}+n_j}s_j - 1 \quad \text{(Equation 9)}$$

The dispersion parameter is optimized with MLE where the likelihood function is:

$$\mathrm{L}(r_j) = \prod_i NB\left(k_{ij}\middle|\widehat{k}_{ij}, r_j\right) \quad \text{(Equation 10)}$$

Fitting the dispersion parameter requires solving an independent convex problem for each peak. To solve a large number of independent convex problems quickly, we implemented a vectorized backtracking line search algorithm using TensorFlow. The dispersion parameter range is set between a lower bound of 0.01 and an upper bound of 1,000 to avoid numerical stability issues and overfitting.

The p value (P_{ij}) of each peak and sample is calculated with a two-sided negative binomial test:

$$P_{\mathrm{ij}} = \min\left\{\frac{1}{2}, \sum_{\mathrm{k}=0}^{\hat{\mathrm{k}}_{\mathrm{ij}}} \mathrm{NB}\left(\mathrm{k}_{\mathrm{ij}}\middle|\widehat{\mathrm{k}}_{\mathrm{ij}}, \mathrm{r}_{\mathrm{j}}\right), 1 - \sum_{\mathrm{k}=0}^{\hat{\mathrm{k}}_{\mathrm{ij}}-1} \mathrm{NB}\left(\mathrm{k}_{\mathrm{ij}}\middle|\widehat{\mathrm{k}}_{\mathrm{ij}}, \mathrm{r}_{\mathrm{j}}\right)\right\} \quad \text{(Equation 11)}$$

Finally, p values are corrected for multiple testing corrections to control the false discovery rate with the Benjamini-Yekutieli method.

In addition to p values, we report the log of fold-change or the log ratio of observed read counts to expected read counts calculated by:

$$l_{ij} = \log_2(FC) = \log_2\left(\frac{k_{ij}+1}{\widehat{k}_{ij}+1}\right) \quad \text{(Equation 12)}$$

and Z score based on fold changes defined as:

$$Z\text{-score}_{ij} = \frac{l_{ij} - \mu_j^l}{\sigma_j^l} \quad \text{(Equation 13)}$$

We consider peaks as outliers if their adjusted p values are smaller than 5% ($P_{\mathrm{adj}} < 0.05$), their absolute log fold changes are greater than 50% ($|\log_2(\mathrm{FC})| > 0.5$) and either their read counts (k_{ij}) or expected read counts ($\widehat{k}_{ij}$) are at least 50.

All the statistics calculated during the outlier detection are stored in anndata[78] file format.

Injection of artificial outliers

In order to benchmark the performance of outlier detection methods, we conducted a simulation experiment using artificial outliers. We created an outlier mask M_{ij} for sample-peak pairs. A sample-region pair is categorized as either an over-accessibility or under-accessibility outlier with a probability of 0.01%:

$$M_{ij} = \begin{cases} 1, \textit{with probability} = 0.0005 \\ -1, \textit{with probability} = 0.0005 \\ 0, \textit{with probability} = 0.999 \end{cases} \quad \text{(Equation 14)}$$

Log-normalized counts are updated using an artificial outlier mask:

$$x_{ij}^{inj} = x_{ij} + \sigma_j e^{\mathcal{N}(3,1)} M_{ij} \quad \text{(Equation 15)}$$

where the deviation in accessibility outlier is simulated by scaling the standard deviation of the peak (σ_j) using a value sampled from a log-normal distribution parameterized by a mean of 3 and a standard deviation of 1. Equation 9 transforms log-transformed normalized injected counts (x_{ij}^{inj}) back to counts in the natural count scale (k_{ij}^{inj}).

Outlier detection methods are evaluated by benchmarking their capability to predict the outlier mask (M_{ij}) from the injected counts (x_{ij}^{inj}). The primary benchmark metric for this evaluation is the area under the precision-recall curve (auPRC). We generated 10 outlier masks for testing and one additional mask for the validation set. Hyperparameter tuning was performed on the validation set to identify the optimal bottleneck size of autoencoder-based models. The evaluation procedure was executed 10 times on the test folds, and the average auPRC performance and its standard deviation are reported.

We evaluated four methods: naive negative binomial test, PCA, OUTRIDER, and EpiOut. During the precision-recall calculation, predictions for each method were primarily ranked using p values, except for PCA, which was ranked by its Z score. The performance of each method at a specific cutoff (either at the adjusted p values of 0.05 or the absolute Z score of 2) is indicated on the precision-recall curve.

For the naive negative binomial test, we averaged counts across samples per peak to obtain the mean parameter of the negative binomial distribution, $NB(k_{ij}^{inj}|\bar{k}_{ij}^{inj}, r_j)$. The dispersion is estimated with MLE (Equation 10).

To evaluate the PCA model, we used PCA to estimate the expected read counts, $\widehat{k}_j^{inj}$. These expected read counts are the reconstructed read counts of PCA. The number of principal components retained was determined through hyperparameter tuning on the validation set. Predictions were subsequently ranked based on the Z score, as formulated in Equation 13, using the expected counts from PCA. We did not compute dispersion or perform a negative binomial test.

We ran OUTRIDER and EpiOut with their default parameters. The bottleneck size of all autoencoder-based methods was selected based on the hyperparameter tuning on the validation set.

Cross-correlation between sample

We used Pearson's correlation to calculate the cross-correlation between samples. The raw cross-correlations were obtained using observed counts (k_{ij}) without any transformation. The cross-correlation after correction was calculated using corrected counts:

$$k_{ij}^{corrected} = k_{ij} - \widehat{k}_{ij} \quad \text{(Equation 16)}$$

Runtime benchmark

To measure runtime to call outliers with OUTRIDER and EpiOut, we subset peaks into groups of 10,000, 25,000, 50,000, and 100,000 and calculated runtime for each input size. Eight CPU cores are used during the benchmark. The analysis is repeated 10 times. The average runtime and standard deviation of runtime were reported. We ran each tool with bottleneck sizes of 5, 10, 50, and 100 in each iteration to avoid runtime differences resulting from hyperparameter tuning. We reported the total runtime across different bottleneck sizes.

Functional annotation of accessible regions with ChIP-seq

We introduced EpiAnnot, a software tool for functional annotation of accessible genomic regions using ChIP-seq marks. If a specific ChIP-seq for a cell line or tissue is present in the Roadmap Epigenomics[34] or ENCODE[35] databases, EpiAnnot can retrieve ChIP-seq data from these public data sources. Additionally, users can annotate their accessible regions using their own custom ChIP-seq data.

EpiAnnot can also attribute accessible regions to genomic features using a gene transfer format (GTF) file. For instance, it annotates accessible regions as 5′ UTR, TSS, etc. In scenarios where an accessible region overlaps with the H3K4me3 histone ChIP-seq mark and TSS or 5′ UTR of a gene, EpiAnnot designates these regions as promoters. Conversely, regions intersecting with the H3K4me1 histone ChIP-seq mark are labeled as enhancers. When the histone mark is available, enhancers overlapping with H3K27ac are categorized as active; otherwise, they are poised. EpiAnnot also annotates enhancers as proximal or distal based on their distance from genes. Specifically, enhancers within a gene body or located up to 10,000 bp upstream or 2,000 bp downstream of a gene are tagged as proximal. All others are marked as distal enhancers.

By using EpiAnnot with H3K4me3, H3K27ac, and H3K4me1 histone ChIP-seq marks from *in vitro* differentiated motor neurons from ALS and clinically healthy samples,[36] we annotated accessible regions from ATAC-seq experiments and used the GTF file of GENCODE v38. The histone ChIP-seq data were downloaded from ENCODE.

Enrichment of outlier pairs in the proximity

To investigate the potential interaction between nearby outlier pairs, we quantified the pairs of accessible regions within predefined distances, irrespective of the outlier status of regions. Then, we applied Fisher's exact test to calculate the enrichment of outlier pairs, using a contingency table structured with the outlier statuses of accessible regions in the pair. We repeated this analysis for 10,000, 100,000, 500,000, and 1,000,000 bp distances. The odds ratio was also calculated to highlight the likelihood of the region being an outlier, given the outlier status of the nearby region.

Annotation of chromatin interactions with Hi-C

To delve deeper into the interactions between outliers, we annotated their interactions based on Hi-C contacts using EpiAnnot. We downloaded public Hi-C data from motor neurons differentiated from iPSCs[36] from ENCODE and computed contact scores based on the number of Hi-C reads between accessible regions using EpiAnnot. For the interaction analysis, the genome is partitioned into bins of 5,000 bp. The interaction between a first source region (s) and a target second region (t) is determined by the highest Hi-C contact score between these bins or their immediate neighbors:

$$h_{s \to t} = \max\left\{HiC_{score}\left(bin_i, bin_j\right), i \in \{t-1, t, t+1\}, j \in \{s-1, s, s+1\}\right\} \quad \text{(Equation 17)}$$

We fitted a power regression to estimate Hi-C contact scores between pairs of accessible regions, using the outlier status of the pairs while controlling for distance. p values for the regression coefficients were calculated using a t test.

We utilized EpiAnnot to compute the ABC score, which signifies potential interactions between regions informed by Hi-C contact scores. The ABC score is defined as:

$$\text{ABC}_{s \to t} = \frac{h_{s \to t} * k_t}{\sum_{i \in vicinity(s)} h_{s \to i} * k_i} \quad \text{(Equation 18)}$$

where the numerator represents the Hi-C contact score between regions multiplied by the accessibility of the target region ($k_t = \sum_i k_{ij}$) and the denominator normalizes this value for all region pairs associated with the source region.

Aberrant gene expression prediction from chromatin accessibility

Gene expression outliers were called with OUTRIDER using the DROP pipeline. If genes were deviant by at least an absolute log fold-change of 30% ($|\log_2(\text{FC})| > 0.3$), and their adjusted p values based on the negative binomial test were smaller than 5% ($P_{adj} < 0.05$), they were considered expression outliers. These gene expression outliers constituted the ground truth labels in the benchmark. In order to predict gene expression outliers from accessibility outliers, we trained an explainable boosting machine (EBM). The EBM model was trained and tested with 10-fold cross-validation. The model aggregates features from promoters and enhancers as input to predict the probability of a gene being categorized as an expression outlier. For predicting a gene's outlier status, the model considers the following features: log fold change, p value, and outlier status of the transcript start site, maximum absolute log fold change of the outliers in proximity, and the ABC score weighted absolute log fold change of the distal outliers. The score for the weighted distal enhancer is computed as follows:

$$\text{enhancer}_{distal} = \sum_{enhancer \in g} \left|ABC_{promoter \to enhancer} * log_2\left(FC_{enhancer}\right)\right| \quad \text{(Equation 19)}$$

Depletion of rare variants with certain consequences in genes with promoter outlier

We assessed the depletion of potentially NMD-triggering rare variants in the gene expression outliers where promoters of these genes are also outliers. Variants are annotated with SnpEff v4.3[41] according to their consequences for genes. Variants with splice acceptor, donor, nonsense, and frameshift consequences were considered as potentially NMD-triggering. Only low-frequency variants with a minor allele frequency ranging from 1% to 0.5% and rare variants with a frequency below 0.5% were included in the analysis. Variant frequencies are downloaded from the gnomAD database.[42] Consequence categories used for depletion analysis include stop codon, frameshift, acceptor, donor site dinucleotides, and missense variants. AbSplice was used to classify

splicing-disrupting exonic or intronic variants using thresholds of 0.05, as advised by the authors. We determined the proportion of genes affected by each variant category based on the outlier status of the genes and their associated promoters. The statistical significance was calculated with the hypergeometric test. Moreover, we analyzed proteomics data to quantify the proportion of proteins impacted by NMD-triggering or missense variants. Z scores for the proteomics data were obtained with PROTRIDER.

Enrichment of SNVs, indels, and structural variants in chromatin accessibility outliers

To evaluate the enrichment of genetic variants in chromatin accessibility outliers, we measured the frequency of SNVs, indels, and structural variants present near chromatin accessibility outliers within a 25 kbp distance. Structural variants from the WGS data were identified using GRIDSS.[79] The variants longer than 50 bp are considered structural variants. The statistical significance was calculated using a hypergeometric test. Only variants with an allele frequency $< 1\%$ were included in the analysis.

Curation of genes associated with neurodegenerative diseases

We manually curated a list of ALS genes from the literature[1,11,46–51,53–55,80–89] and ALSOD.[45] The curated list is available at 10.5281/zenodo.8331545. Moreover, we queried the OMIM[69] database using "neurodegenerative" and "neurodegeneration" keywords, filtered the retrieved entries based on neurologic clinical synopsis, and generated a list of genes associated with neurodegenerative diseases via the REST API. The genes were further annotated for their LoF intolerance obtained from gnomAD.[42] Genes are considered LoF intolerant if their LoF observed/expected upper bound fraction (LOEUF) is below 35%.

Data and code availability

All the analyses in the paper are implemented in the reproducible snakemake[90] format available at github.com/uci-cbcl/ALS-accessibility-outliers-paper. EpiOut, the Python package for outlier detection and annotation, is available at github.com/uci-cbcl/EpiOut. Data used in the preparation of this article were obtained from the ANSWER ALS Data Portal (AALS-01184). For up-to-date information on the study, visit https://dataportal.answerals.org/.

Supplemental information

Supplemental information can be found online at https://doi.org/10.1016/j.xhgg.2024.100318.

Acknowledgments

This project is funded by a grant from the Robert Packard Center for ALS Research at Johns Hopkins and grants from the National Science Foundation (IIS-1715017, DMS-1763272) and received UCI IPH Pilot Award.

Declaration of interests

The authors declare no competing interests.

Received: November 17, 2023
Accepted: June 11, 2024

References

1. Hardiman, O., Al-Chalabi, A., Chio, A., Corr, E.M., Logroscino, G., Robberecht, W., Shaw, P.J., Simmons, Z., and van den Berg, L.H. (2017). Amyotrophic lateral sclerosis. Nat. Rev. Dis. Prim. *3*, 17071–17119.
2. Longinetti, E., and Fang, F. (2019). Epidemiology of amyotrophic lateral sclerosis: an update of recent literature. Curr. Opin. Neurol. *32*, 771–776.
3. Masrori, P., and Van Damme, P. (2020). Amyotrophic lateral sclerosis: a clinical review. Eur. J. Neurol. *27*, 1918–1929.
4. Al-Chalabi, A., Fang, F., Hanby, M.F., Leigh, P.N., Shaw, C.E., Ye, W., and Rijsdijk, F. (2010). An estimate of amyotrophic lateral sclerosis heritability using twin data. J. Neurol. Neurosurg. Psychiatry *81*, 1324–1326.
5. Grassano, M., Calvo, A., Moglia, C., Sbaiz, L., Brunetti, M., Barberis, M., Casale, F., Manera, U., Vasta, R., Canosa, A., et al. (2022). Systematic evaluation of genetic mutations in ALS: a population-based study. J. Neurol. Neurosurg. Psychiatry *93*, 1190–1193.
6. Renton, A.E., Chiò, A., and Traynor, B.J. (2014). State of play in amyotrophic lateral sclerosis genetics. Nat. Neurosci. *17*, 17–23.
7. Akçimen, F., Lopez, E.R., Landers, J.E., Nath, A., Chiò, A., Chia, R., and Traynor, B.J. (2023). Amyotrophic lateral sclerosis: translating genetic discoveries into therapies. Nat. Rev. Genet. *24*, 642–658.
8. Project MinE ALS Sequencing Consortium (2018). Project MinE: study design and pilot analyses of a large-scale whole-genome sequencing study in amyotrophic lateral sclerosis. Eur. J. Hum. Genet. *26*, 1537–1546.
9. Baxi, E.G., Thompson, T., Li, J., Kaye, J.A., Lim, R.G., Wu, J., Ramamoorthy, D., Lima, L., Vaibhav, V., Matlock, A., et al. (2022). Answer ALS, a large-scale resource for sporadic and familial ALS combining clinical and multi-omics data from induced pluripotent cell lines. Nat. Neurosci. *25*, 226–237.
10. van Rheenen, W., van der Spek, R.A.A., Bakker, M.K., van Vugt, J.J.F.A., Hop, P.J., Zwamborn, R.A.J., de Klein, N., Westra, H.J., Bakker, O.B., Deelen, P., et al. (2021). Common and rare variant association analyses in amyotrophic lateral sclerosis identify 15 risk loci with distinct genetic architectures and neuron-specific biology. Nat. Genet. *53*, 1636–1648.
11. Gregory, J.M., Fagegaltier, D., Phatnani, H., and Harms, M.B. (2020). Genetics of Amyotrophic Lateral Sclerosis. Curr. Genet. Med. Rep. *8*, 121–131.
12. NeuroLINCS Consortium, Li, J., Lim, R.G., Kaye, J.A., Dardov, V., Coyne, A.N., Wu, J., Milani, P., Cheng, A., Thompson, T.G., et al. (2021). An integrated multi-omic analysis of iPSC-derived motor neurons from C9ORF72 ALS patients. iScience *24*, 103221.
13. Frésard, L., Smail, C., Ferraro, N.M., Teran, N.A., Li, X., Smith, K.S., Bonner, D., Kernohan, K.D., Marwaha, S., Zappala, Z., et al. (2019). Identification of rare-disease genes using blood transcriptome sequencing and large control cohorts. Nat. Med. *25*, 911–919.
14. Mertes, C., Scheller, I.F., Yépez, V.A., Çelik, M.H., Liang, Y., Kremer, L.S., Gusic, M., Prokisch, H., and Gagneur, J. (2021).

Detection of aberrant splicing events in RNA-seq data using FRASER. Nat. Commun. *12*, 529.

15. Yépez, V.A., Mertes, C., Müller, M.F., Klaproth-Andrade, D., Wachutka, L., Frésard, L., Gusic, M., Scheller, I.F., Goldberg, P.F., Prokisch, H., and Gagneur, J. (2021). Detection of aberrant gene expression events in RNA sequencing data. Nat. Protoc. *16*, 1276–1296.
16. Li, X., Kim, Y., Tsang, E.K., Davis, J.R., Damani, F.N., Chiang, C., Hess, G.T., Zappala, Z., Strober, B.J., Scott, A.J., et al. (2017). The impact of rare variation on gene expression across tissues. Nature *550*, 239–243.
17. Marwaha, S., Knowles, J.W., and Ashley, E.A. (2022). A guide for the diagnosis of rare and undiagnosed disease: beyond the exome. Genome Med. *14*, 23.
18. Jenkinson, G., Li, Y.I., Basu, S., Cousin, M.A., Oliver, G.R., and Klee, E.W. (2020). LeafCutterMD: an algorithm for outlier splicing detection in rare diseases. Bioinformatics *36*, 4609–4615.
19. Kremer, L.S., Bader, D.M., Mertes, C., Kopajtich, R., Pichler, G., Iuso, A., Haack, T.B., Graf, E., Schwarzmayr, T., Terrile, C., et al. (2017). Genetic diagnosis of Mendelian disorders via RNA sequencing. Nat. Commun. *8*, 15824.
20. Kopajtich, R., Smirnov, D., Stenton, S.L., Loipfinger, S., Meng, C., Scheller, I.F., Freisinger, P., Baski, R., Berutti, R., Behr, J., et al. (2021). Integration of proteomics with genomics and transcriptomics increases the diagnostic rate of Mendelian disorders. Preprint at medRxiv. https://doi.org/10.1101/2021.03.09.21253187.
21. Li, T., Ferraro, N., Strober, B.J., Aguet, F., Kasela, S., Arvanitis, M., Ni, B., Wiel, L., Hershberg, E., Ardlie, K., et al. (2023). The functional impact of rare variation across the regulatory cascade. Cell Genom. *3*, 100401. https://doi.org/10.1016/j.xgen.2023.100401.
22. Klemm, S.L., Shipony, Z., and Greenleaf, W.J. (2019). Chromatin accessibility and the regulatory epigenome. Nat. Rev. Genet. *20*, 207–220.
23. Liu, Q., Zaba, L.C., Satpathy, A.T., Longmire, M., Zhang, W., Li, K., Granja, J., Guo, C., Lin, J., Li, R., et al. (2020). Chromatin accessibility landscapes of skin cells in systemic sclerosis nominate dendritic cells in disease pathogenesis. Nat. Commun. *11*, 5843.
24. Turner, A.W., Hu, S.S., Mosquera, J.V., Ma, W.F., Hodonsky, C.J., Wong, D., Auguste, G., Song, Y., Sol-Church, K., Farber, E., et al. (2022). Single-nucleus chromatin accessibility profiling highlights regulatory mechanisms of coronary artery disease risk. Nat. Genet. *54*, 804–816.
25. Morabito, S., Miyoshi, E., Michael, N., Shahin, S., Martini, A.C., Head, E., Silva, J., Leavy, K., Perez-Rosendahl, M., and Swarup, V. (2021). Single-nucleus chromatin accessibility and transcriptomic characterization of Alzheimer's disease. Nat. Genet. *53*, 1143–1155.
26. Yan, F., Powell, D.R., Curtis, D.J., and Wong, N.C. (2020). From reads to insight: a hitchhiker's guide to ATAC-seq data analysis. Genome Biol. *21*, 22.
27. Abadi, M., Agarwal, A., Barham, P., Brevdo, E., Chen, Z., Citro, C., Corrado, G.S., Davis, A., Dean, J., Devin, M., et al. (2016). Tensorflow: Large-scale machine learning on heterogeneous distributed systems. Preprint at arXiv. *arXiv:1603.04467*.
28. Zhang, Y., Liu, T., Meyer, C.A., Eeckhoute, J., Johnson, D.S., Bernstein, B.E., Nusbaum, C., Myers, R.M., Brown, M., Li, W., and Liu, X.S. (2008). Model-based analysis of ChIP-Seq (MACS). Genome Biol. *9*, R137.
29. Stovner, E.B., and Sætrom, P. (2020). PyRanges: efficient comparison of genomic intervals in Python. Bioinforma. Oxf. Engl. *36*, 918–919.
30. Quinlan, A.R., and Hall, I.M. (2010). BEDTools: a flexible suite of utilities for comparing genomic features. Bioinformatics *26*, 841–842.
31. Pedersen, B.S., Layer, R.M., and Quinlan, A.R. (2016). Vcfanno: fast, flexible annotation of genetic variants. Genome Biol. *17*, 118.
32. Love, M.I., Huber, W., and Anders, S. (2014). Moderated estimation of fold change and dispersion for RNA-seq data with DESeq2. Genome Biol. *15*, 550.
33. Brechtmann, F., Mertes, C., Matusevičiūtė, A., Yépez, V.A., Avsec, Ž., Herzog, M., Bader, D.M., Prokisch, H., and Gagneur, J. (2018). OUTRIDER: A Statistical Method for Detecting Aberrantly Expressed Genes in RNA Sequencing Data. Am. J. Hum. Genet. *103*, 907–917.
34. Roadmap Epigenomics Consortium, Kundaje, A., Meuleman, W., Ernst, J., Bilenky, M., Yen, A., Heravi-Moussavi, A., Kheradpour, P., Zhang, Z., Wang, J., et al. (2015). Integrative analysis of 111 reference human epigenomes. Nature *518*, 317–330.
35. ENCODE Project Consortium (2012). An integrated encyclopedia of DNA elements in the human genome. Nature *489*, 57–74.
36. Zhang, S., Cooper-Knock, J., Weimer, A.K., Shi, M., Moll, T., Marshall, J.N.G., Harvey, C., Nezhad, H.G., Franklin, J., Souza, C.D.S., et al. (2022). Genome-wide identification of the genetic basis of amyotrophic lateral sclerosis. Neuron *110*, 992–1008.e11.
37. Heintzman, N.D., Stuart, R.K., Hon, G., Fu, Y., Ching, C.W., Hawkins, R.D., Barrera, L.O., Van Calcar, S., Qu, C., Ching, K.A., et al. (2007). Distinct and predictive chromatin signatures of transcriptional promoters and enhancers in the human genome. Nat. Genet. *39*, 311–318.
38. Fulco, C.P., Nasser, J., Jones, T.R., Munson, G., Bergman, D.T., Subramanian, V., Grossman, S.R., Anyoha, R., Doughty, B.R., Patwardhan, T.A., et al. (2019). Activity-by-contact model of enhancer–promoter regulation from thousands of CRISPR perturbations. Nat. Genet. *51*, 1664–1669.
39. Nori, H., Jenkins, S., Koch, P., and Caruana, R. (2019). InterpretML: A Unified Framework for Machine Learning Interpretability. Preprint at arXiv. https://doi.org/10.48550/arXiv.1909.09223.
40. Ernst, J., and Kellis, M. (2017). Chromatin-state discovery and genome annotation with ChromHMM. Nat. Protoc. *12*, 2478–2492.
41. Cingolani, P., Platts, A., Wang, L.L., Coon, M., Nguyen, T., Wang, L., Land, S.J., Lu, X., and Ruden, D.M. (2012). A program for annotating and predicting the effects of single nucleotide polymorphisms, SnpEff: SNPs in the genome of Drosophila melanogaster strain w1118; iso-2; iso-3. Fly *6*, 80–92.
42. Karczewski, K.J., Francioli, L.C., Tiao, G., Cummings, B.B., Alföldi, J., Wang, Q., Collins, R.L., Laricchia, K.M., Ganna, A., Birnbaum, D.P., et al. (2020). The mutational constraint spectrum quantified from variation in 141,456 humans. Nature *581*, 434–443.
43. Daguenet, E., Dujardin, G., and Valcárcel, J. (2015). The pathogenicity of splicing defects: mechanistic insights into pre-mRNA processing inform novel therapeutic approaches. EMBO Rep. *16*, 1640–1655.

44. Çelik, M.H., et al. (2022). Aberrant splicing prediction across human tissues. Preprint at bioRxiv. https://doi.org/10.1101/2022.06.13.495326.
45. Abel, O., Powell, J.F., Andersen, P.M., and Al-Chalabi, A. (2012). ALSoD: A user-friendly online bioinformatics tool for amyotrophic lateral sclerosis genetics. Hum. Mutat. *33*, 1345–1351.
46. Pecoraro, V., Mandrioli, J., Carone, C., Chiò, A., Traynor, B.J., and Trenti, T. (2020). The NGS technology for the identification of genes associated with the ALS. A systematic review. Eur. J. Clin. Invest. *50*, e13228.
47. Cheng, W., Wang, S., Zhang, Z., Morgens, D.W., Hayes, L.R., Lee, S., Portz, B., Xie, Y., Nguyen, B.V., Haney, M.S., et al. (2019). CRISPR-Cas9 Screens Identify the RNA Helicase DDX3X as a Repressor of C9ORF72 (GGGGCC)n Repeat-Associated Non-AUG Translation. Neuron *104*, 885–898.e8.
48. Krach, F., Wheeler, E.C., Regensburger, M., Boerstler, T., Wend, H., Vu, A.Q., Wang, R., Reischl, S., Boldt, K., Batra, R., et al. (2022). Aberrant NOVA1 function disrupts alternative splicing in early stages of amyotrophic lateral sclerosis. Acta Neuropathol. *144*, 413–435.
49. Mòdol-Caballero, G., García-Lareu, B., Verdés, S., Ariza, L., Sánchez-Brualla, I., Brocard, F., Bosch, A., Navarro, X., and Herrando-Grabulosa, M. (2020). Therapeutic Role of Neuregulin 1 Type III in SOD1-Linked Amyotrophic Lateral Sclerosis. Neurotherapeutics *17*, 1048–1060.
50. Blauw, H.M., van Rheenen, W., Koppers, M., Van Damme, P., Waibel, S., Lemmens, R., van Vught, P.W.J., Meyer, T., Schulte, C., Gasser, T., et al. (2012). NIPA1 polyalanine repeat expansions are associated with amyotrophic lateral sclerosis. Hum. Mol. Genet. *21*, 2497–2502.
51. Tazelaar, G.H., Dekker, A.M., van Vugt, J.J., van der Spek, R.A., Westeneng, H.J., Kool, L.J., Kenna, K.P., van Rheenen, W., Pulit, S.L., McLaughlin, R.L., et al. (2019). Association of NIPA1 repeat expansions with amyotrophic lateral sclerosis in a large international cohort. Neurobiol. Aging *74*, 234.e9–234.e15.
52. Poulos, R.C., Hains, P.G., Shah, R., Lucas, N., Xavier, D., Manda, S.S., Anees, A., Koh, J.M.S., Mahboob, S., Wittman, M., et al. (2020). Strategies to enable large-scale proteomics for reproducible research. Nat. Commun. *11*, 3793.
53. Berson, A., Goodman, L.D., Sartoris, A.N., Otte, C.G., Aykit, J.A., Lee, V.M.Y., Trojanowski, J.Q., and Bonini, N.M. (2019). Drosophila Ref1/ALYREF regulates transcription and toxicity associated with ALS/FTD disease etiologies. Acta Neuropathol. Commun. *7*, 65.
54. Ong, H.W., Liang, Y., Richardson, W., Lowry, E.R., Wells, C.I., Chen, X., Silvestre, M., Dempster, K., Silvaroli, J.A., Smith, J.L., et al. (2023). Discovery of a Potent and Selective CDKL5/GSK3 Chemical Probe That Is Neuroprotective. ACS Chem. Neurosci. *14*, 1672–1685.
55. Nomura, E., Ohta, Y., Tadokoro, K., Shang, J., Feng, T., Liu, X., Shi, X., Matsumoto, N., Sasaki, R., Tsunoda, K., et al. (2019). Imaging Hypoxic Stress and the Treatment of Amyotrophic Lateral Sclerosis with Dimethyloxalylglycine in a Mice Model. Neuroscience *415*, 31–43.
56. Coyne, A.N., and Rothstein, J.D. (2021). The ESCRT-III protein VPS4, but not CHMP4B or CHMP2B, is pathologically increased in familial and sporadic ALS neuronal nuclei. Acta Neuropathol. Commun. *9*, 127.
57. Stevens, C.H., Guthrie, N.J., van Roijen, M., Halliday, G.M., and Ooi, L. (2019). Increased Tau Phosphorylation in Motor Neurons From Clinically Pure Sporadic Amyotrophic Lateral Sclerosis Patients. J. Neuropathol. Exp. Neurol. *78*, 605–614.
58. Lei, L., Feng, J., Wu, G., Wei, Z., Wang, J.Z., Zhang, B., Liu, R., Liu, F., Wang, X., and Li, H.L. (2022). HIF-1α Causes LCMT1/PP2A Deficiency and Mediates Tau Hyperphosphorylation and Cognitive Dysfunction during Chronic Hypoxia. Int. J. Mol. Sci. *23*, 16140.
59. Dudman, J., and Qi, X. (2020). Stress Granule Dysregulation in Amyotrophic Lateral Sclerosis. Front. Cell. Neurosci. *14*, 598517.
60. Becker, L.A., Huang, B., Bieri, G., Ma, R., Knowles, D.A., Jafar-Nejad, P., Messing, J., Kim, H.J., Soriano, A., Auburger, G., et al. (2017). Therapeutic reduction of ataxin-2 extends lifespan and reduces pathology in TDP-43 mice. Nature *544*, 367–371.
61. Zhang, T., Periz, G., Lu, Y.-N., and Wang, J. (2020). USP7 regulates ALS-associated proteotoxicity and quality control through the NEDD4L–SMAD pathway. Proc. Natl. Acad. Sci. USA *117*, 28114–28125.
62. Hemerková, P., and Vališ, M. (2021). Role of Oxidative Stress in the Pathogenesis of Amyotrophic Lateral Sclerosis: Antioxidant Metalloenzymes and Therapeutic Strategies. Biomolecules *11*, 437.
63. Zhao, J.-M., and Qi, T.-G. (2021). The role of TXNL1 in disease: treatment strategies for cancer and diseases with oxidative stress. Mol. Biol. Rep. *48*, 2929–2934.
64. Yu, J.-T., Liu, Y., Dong, P., Cheng, R.E., Ke, S.X., Chen, K.Q., Wang, J.J., Shen, Z.S., Tang, Q.Y., and Zhang, Z. (2019). Up-regulation of antioxidative proteins TRX1, TXNL1 and TXNRD1 in the cortex of PTZ kindling seizure model mice. PLoS One *14*, e0210670.
65. Tran, D., Chalhoub, A., Schooley, A., Zhang, W., and Ngsee, J.K. (2012). A mutation in VAPB that causes amyotrophic lateral sclerosis also causes a nuclear envelope defect. J. Cell Sci. *125*, 2831–2836.
66. Mann, J.R., McKenna, E.D., Mawrie, D., Papakis, V., Alessandrini, F., Anderson, E.N., Mayers, R., Ball, H.E., Kaspi, E., Lubinski, K., et al. (2023). Loss of function of the ALS-associated NEK1 kinase disrupts microtubule homeostasis and nuclear import. Sci. Adv. *9*, eadi5548.
67. Zhang, Y., Zhu, M., Zheng, C., Wei, W., Emmer, B.T., and Zhang, B. (2022). LMAN1–MCFD2 complex is a cargo receptor for the ER-Golgi transport of α1-antitrypsin. Biochem. J. *479*, 839–855.
68. Fu, Y.-L., Zhang, B., and Mu, T.-W. (2019). LMAN1 (ERGIC-53) promotes trafficking of neuroreceptors. Biochem. Biophys. Res. Commun. *511*, 356–362.
69. Hamosh, A., Scott, A.F., Amberger, J.S., Bocchini, C.A., and McKusick, V.A. (2005). Online Mendelian Inheritance in Man (OMIM), a knowledgebase of human genes and genetic disorders. Nucleic Acids Res. *33*, D514–D517.
70. Taghdiri, M., Dastsooz, H., Fardaei, M., Mohammadi, S., Farazi Fard, M.A., and Faghihi, M.A. (2017). A Novel Mutation in ERCC8 Gene Causing Cockayne Syndrome. Front. Pediatr. *5*, 169.
71. Weidenheim, K.M., Dickson, D.W., and Rapin, I. (2009). Neuropathology of Cockayne syndrome: Evidence for impaired development, premature aging, and neurodegeneration. Mech. Ageing Dev. *130*, 619–636.
72. Tian, Y., Ma, G., Li, H., Zeng, Y., Zhou, S., Wang, X., Shan, S., Xu, Y., Xiong, J., and Cheng, G. (2023). Shared Genetics and Comorbid Genes of Amyotrophic Lateral Sclerosis and Parkinson's Disease. Mov. Disord. *38*, 1813–1821.

73. Zhou, H.J., Li, L., Li, Y., Li, W., and Li, J.J. (2022). PCA outperforms popular hidden variable inference methods for molecular QTL mapping. Genome Biol. *23*, 210.
74. Townes, F.W., Hicks, S.C., Aryee, M.J., and Irizarry, R.A. (2019). Feature selection and dimension reduction for single-cell RNA-Seq based on a multinomial model. Genome Biol. *20*, 295.
75. Porcu, E., Rüeger, S., Lepik, K., eQTLGen Consortium; and BIOS Consortium, Santoni, F.A., Reymond, A., and Kutalik, Z. (2019). Mendelian randomization integrating GWAS and eQTL data reveals genetic determinants of complex and clinical traits. Nat. Commun. *10*, 3300.
76. Wagner, N., Çelik, M.H., Hölzlwimmer, F.R., Mertes, C., Prokisch, H., Yépez, V.A., and Gagneur, J. (2023). Aberrant splicing prediction across human tissues. Nat. Genet. *55*, 861–870.
77. Li, H., Handsaker, B., Wysoker, A., Fennell, T., Ruan, J., Homer, N., Marth, G., Abecasis, G., Durbin, R.; and 1000 Genome Project Data Processing Subgroup (2009). The Sequence Alignment/Map format and SAMtools. Bioinformatics *25*, 2078–2079.
78. Virshup, I., Rybakov, S., Theis, F.J., Angerer, P., and Wolf, F.A. (2021). anndata: Annotated data. Preprint at bioRxiv. https://doi.org/10.1101/2021.12.16.473007.
79. Cameron, D.L., Baber, J., Shale, C., Valle-Inclan, J.E., Besselink, N., van Hoeck, A., Janssen, R., Cuppen, E., Priestley, P., and Papenfuss, A.T. (2021). GRIDSS2: comprehensive characterisation of somatic structural variation using single breakend variants and structural variant phasing. Genome Biol. *22*, 202.
80. Steinberg, K.M., Yu, B., Koboldt, D.C., Mardis, E.R., and Pamphlett, R. (2015). Exome sequencing of case-unaffected-parents trios reveals recessive and de novo genetic variants in sporadic ALS. Sci. Rep. *5*, 9124.
81. Mishra, P.S., Boutej, H., Soucy, G., Bareil, C., Kumar, S., Picher-Martel, V., Dupré, N., Kriz, J., and Julien, J.P. (2020). Transmission of ALS pathogenesis by the cerebrospinal fluid. Acta Neuropathol. Commun. *8*, 65.
82. Rotem, N., Magen, I., Ionescu, A., Gershoni-Emek, N., Altman, T., Costa, C.J., Gradus, T., Pasmanik-Chor, M., Willis, D.E., Ben-Dov, I.Z., et al. (2017). ALS Along the Axons – Expression of Coding and Noncoding RNA Differs in Axons of ALS models. Sci. Rep. *7*, 44500.
83. Hetz, C., Thielen, P., Fisher, J., Pasinelli, P., Brown, R.H., Korsmeyer, S., and Glimcher, L. (2007). The proapoptotic BCL-2 family member BIM mediates motoneuron loss in a model of amyotrophic lateral sclerosis. Cell Death Differ. *14*, 1386–1389.
84. Vukosavic, S., Dubois-Dauphin, M., Romero, N., and Przedborski, S. (2002). Bax and Bcl-2 Interaction in a Transgenic Mouse Model of Familial Amyotrophic Lateral Sclerosis. J. Neurochem. *73*, 2460–2468.
85. Pasinelli, P., Belford, M.E., Lennon, N., Bacskai, B.J., Hyman, B.T., Trotti, D., and Brown, R.H., Jr. (2004). Amyotrophic Lateral Sclerosis-Associated SOD1 Mutant Proteins Bind and Aggregate with Bcl-2 in Spinal Cord Mitochondria. Neuron *43*, 19–30.
86. Yuan, Y., Liu, Z., Hou, X., Li, W., Ni, J., Huang, L., Hu, Y., Liu, P., Hou, X., Xue, J., et al. (2020). Identification of GGC repeat expansion in the *NOTCH2NLC* gene in amyotrophic lateral sclerosis. Neurology *95*, e3394–e3405.
87. Song, F., Chiang, P., Wang, J., Ravits, J., and Loeb, J.A. (2012). Aberrant Neuregulin 1 Signaling in Amyotrophic Lateral Sclerosis. J. Neuropathol. Exp. Neurol. *71*, 104–115.
88. Schwenk, B.M., Hartmann, H., Serdaroglu, A., Schludi, M.H., Hornburg, D., Meissner, F., Orozco, D., Colombo, A., Tahirovic, S., Michaelsen, M., et al. (2016). TDP-43 loss of function inhibits endosomal trafficking and alters trophic signaling in neurons. EMBO J. *35*, 2350–2370.
89. Nardo, G., Pozzi, S., Pignataro, M., Lauranzano, E., Spano, G., Garbelli, S., Mantovani, S., Marinou, K., Papetti, L., Monteforte, M., et al. (2011). Amyotrophic Lateral Sclerosis Multiprotein Biomarkers in Peripheral Blood Mononuclear Cells. PLoS One *6*, e25545.
90. Köster, J., and Rahmann, S. (2012). Snakemake–a scalable bioinformatics workflow engine. Bioinforma. Oxf. Engl. *28*, 2520–2522.

HGGA, Volume *5*

Supplemental information

Identifying dysregulated regions in amyotrophic lateral sclerosis through chromatin accessibility outliers

Muhammed Hasan Çelik, Julien Gagneur, Ryan G. Lim, Jie Wu, Leslie M. Thompson, and Xiaohui Xie

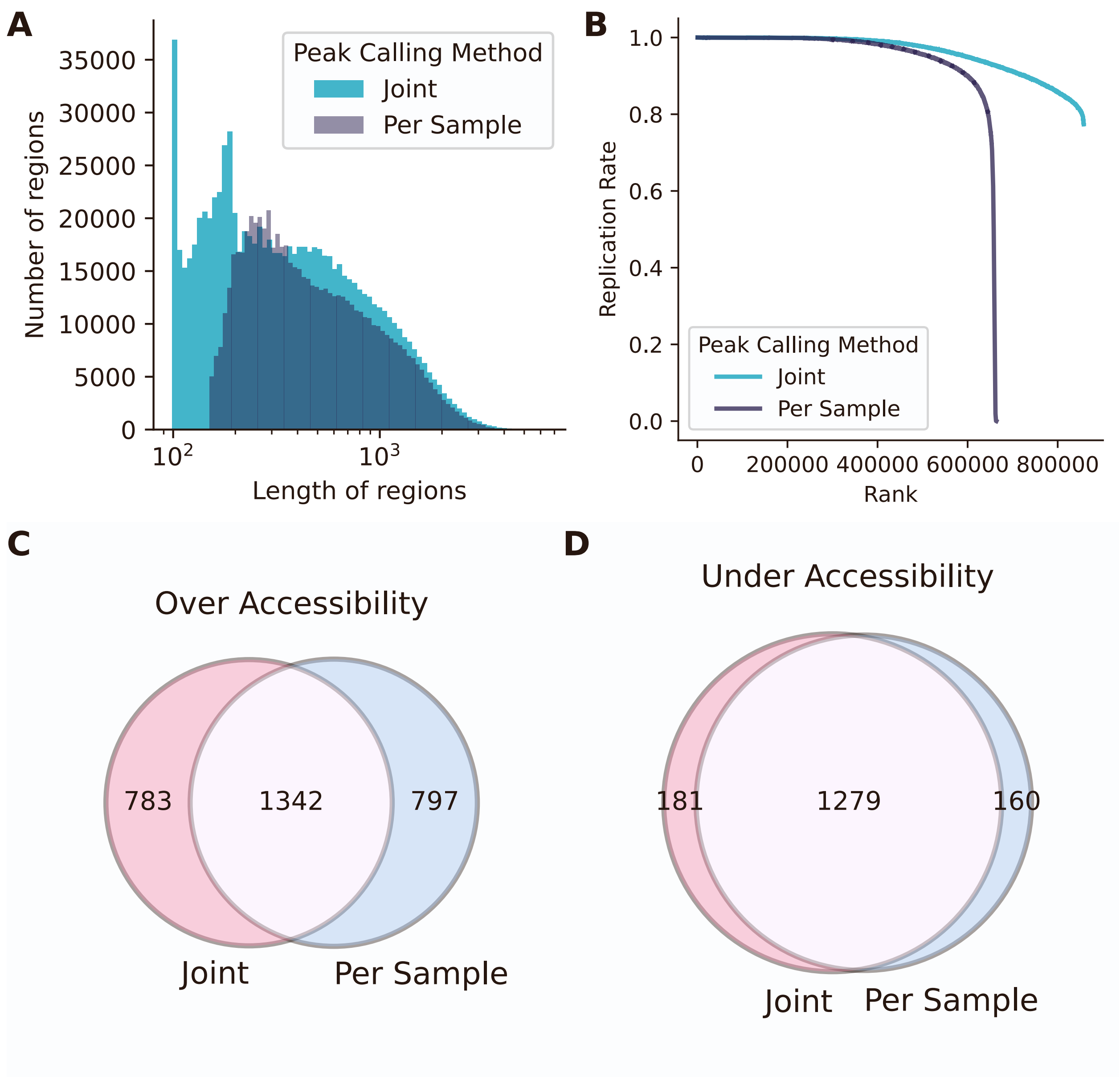

Figure S1. The comparison of joint and per-sample peak calling methods
Peaks are identified using MACS2 in both approaches. For the cohort-wide joint peak calling, all ATAC-seq reads are aggregated into a single BAM file, which is subsequently processed with MACS2 to produce a set of peaks consistent across samples. In the sample-wise peak calling approach, peaks are identified individually for each sample using MACS2, resulting in a separate set of peaks for each sample. Then, overlapping peaks from different samples are collapsed, ensuring a consistent comparison of peaks during the outlier calling. **(A)** The length distribution (in base pairs) of accessible chromatin regions detected with joint and per-sample peak calling. Joint peak calling produces a greater number of peaks that are narrower compared to per-sample peak calling. **(B)** The moving average of the replication rate by the rank of peaks, sorted by total read counts. Joint peak calling exhibits a higher replication rate in regions with lower read depth. **(C)** Overlap of over-accessibility outliers identified through joint and per-sample peak calling methods. Both approaches generate a comparable number of over-accessibility outliers. ~63% of the peaks identified by one method were detected by the other method. **(D)** Overlap

of under-accessibility outliers identified through joint and per-sample peak calling methods. $\sim 88\%$ of the outliers identified by one method were also detected by the other method.

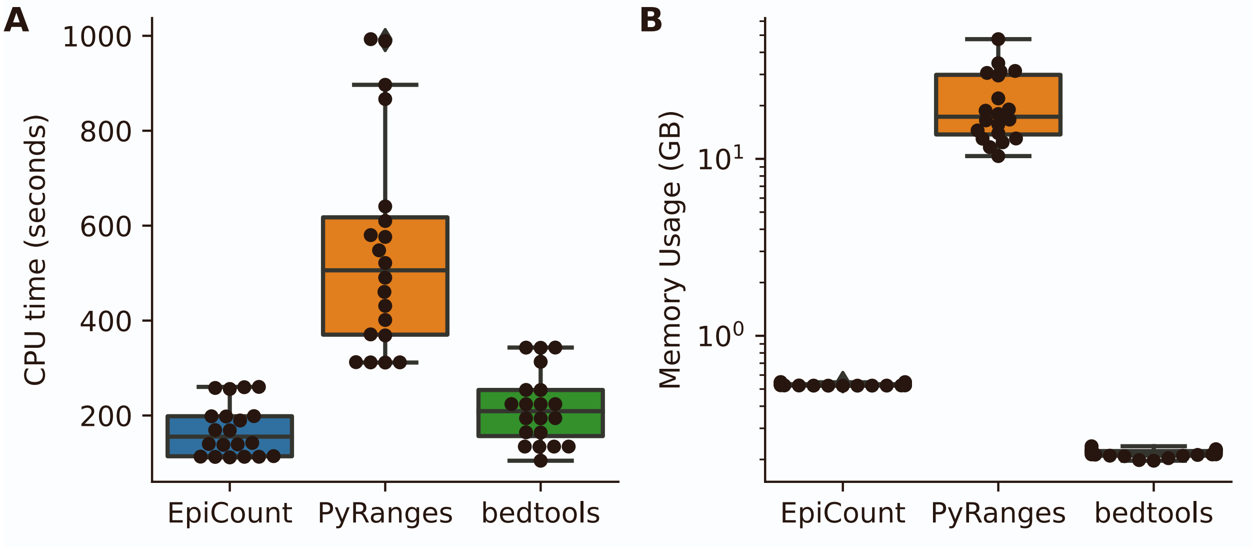

Figure S2. Runtime of counting step.

Bam and bed files for 20 ATAC-seq experiments were downloaded from ENCODE. Each bam file has 142 ± 48 million ATAC-seq reads. Overlapping accessible regions across bed files were collapsed, leading to 365,090 accessible regions. The counting was performed with these accessible regions and bam files using EpiCount, bedtools, and PyRanges. During bedtools counting, bed files are first sorted, then counting is performed using the -*sorted* flag in bedtools to leverage the chrom-sweep algorithm. **(A)** CPU time of counting methods. EpiCount is twice as fast as PyRanges and comparable with bedtools. The mean runtime of EpiOut per bam file is 169 ± 54 seconds; meanwhile, the runtime is 549 ± 225 seconds for PyRanges and 214 ± 74 for bedtools. Epiout counts one million reads per 1.2 ± 0.1 seconds, while PyRanges counts one million reads in 3.8 ± 0.2 seconds and bedtools counts in 1.5 ± 0.1. **(B)** EpiOut and bedtools have a much smaller memory footprint than PyRanges. PyRanges loads the entire bam file to memory while EpiCount and bedtools perform stream counting by only loading a read per iteration; thus, EpiCount (mean memory consumption 0.53 ± 0.009 GB) and bedtools (mean memory consumption 0.21 ± 0.01 GB) consumes ~2% of PyRanges' memory footprint (mean memory consumption 21 ± 10 GB). Extensive memory usage of PyRanges limits the parallelization across samples.

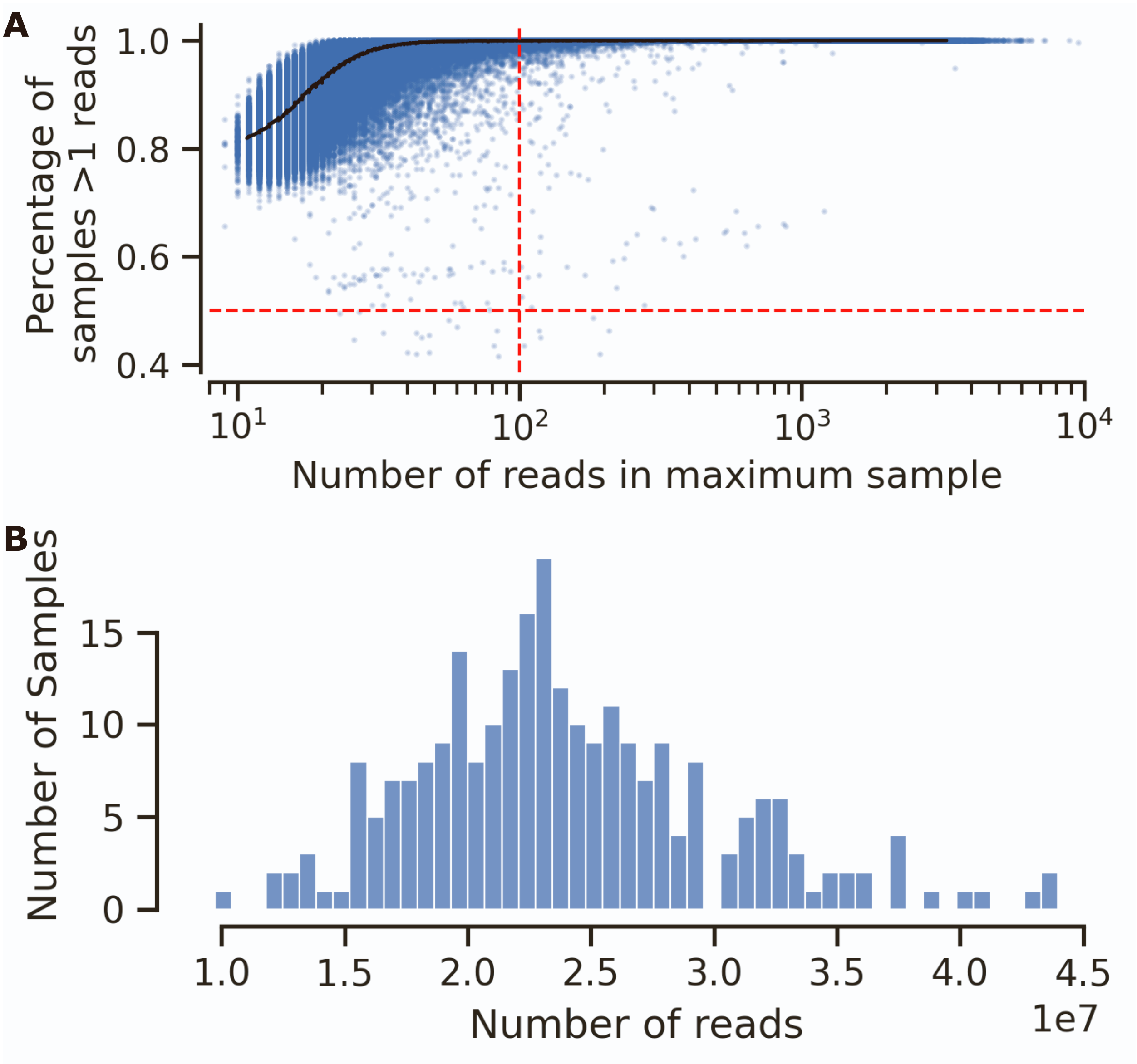

Figure S3. Read coverage and replication rate statistics.

(A) Read coverage distribution across samples. Notable differences in read coverage are observed among the samples, highlighting the importance of size factor normalization to adjust for coverage disparity. **(B)** Replication rate of accessible regions throughout samples. In the scatterplot, each dot signifies an accessible region for a sample. The x-axis denotes the read counts supporting the accessible region in the sample with the highest read count. Filters, represented by the red lines, were implemented to ensure an accessible region is supported by at least 100 reads in one sample and is replicated in at least 50% of the samples by at least 2 reads.

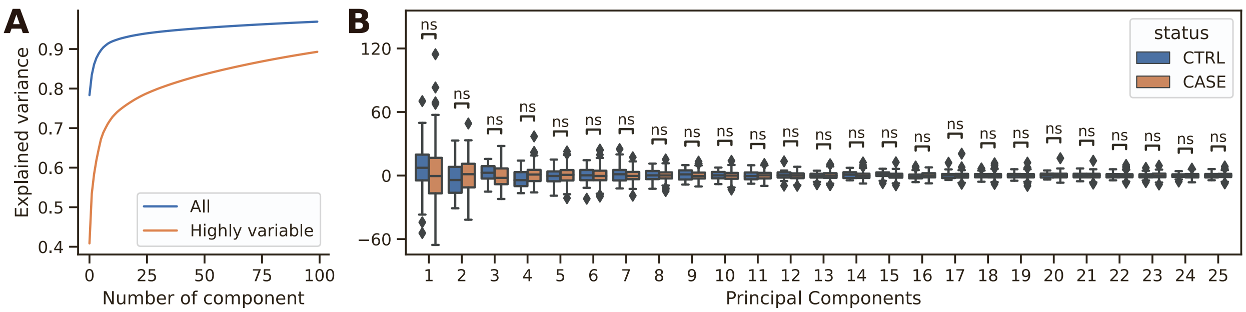

Figure S4. The major covariates in the DNA accessibility data.

(A) Cumulative explained variance by top principal components. The variance of accessibility is calculated based on the raw read counts, and the top 20,000 regions with the highest variance are chosen as highly variable regions. Then, the read counts are normalized for variance stabilizing transformation (VST), and principal component analysis is performed. The top 25 principal components (representing the major latent confounding factors) explain $\sim 79\%$ and $\sim 94\%$ of the accessibility covariation between samples for highly variable and all regions, respectively. **(B)** The distribution of top principal components by the disease status for the highly variable regions. None of the top principal components are associated with the disease status based on the Mann–Whitney U test. P-values are corrected for multiple testing with the Benjamini-Yekutieli false-discovery rate (FDR) method.

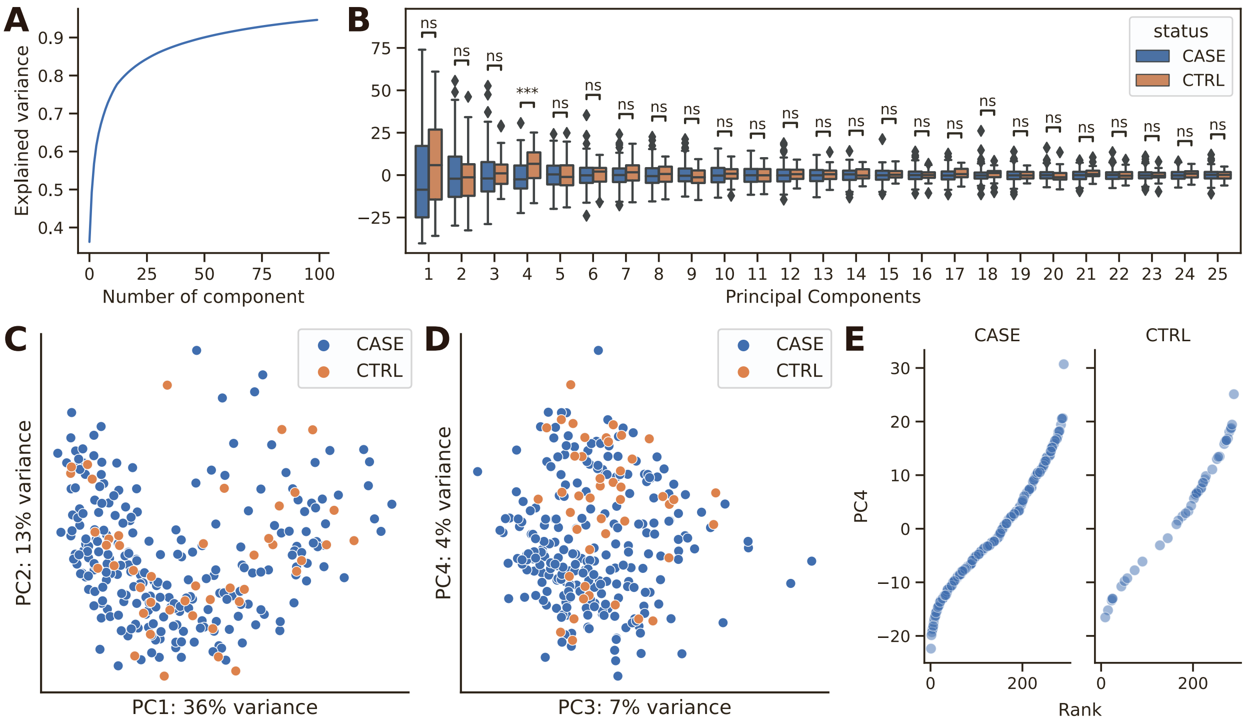

Figure S5. The major covariates in the transcriptomics data.

(A) Cumulative explained variance by top principal components. The variance of gene expression is calculated based on the raw read counts, and the top 10,000 genes with the highest variance are chosen. Then, the read counts are normalized for variance stabilizing transformation (VST), and principal component analysis is performed. The top 25 principal components explain ~79% of the gene expression covariation between samples. **(B)** The distribution of top principal components by the disease status. Only one of the top principal components is associated with the disease status based on the Mann–Whitney U test. P-values are corrected for multiple testing with the Benjamini-Yekutieli false-discovery rate (FDR) method. **(C)** The first, second **(D),** and the third and fourth principal components. **(E)** The distribution of samples by the fourth principal component has slightly higher values for control samples; however, a clear separation between cases and controls is not observed.

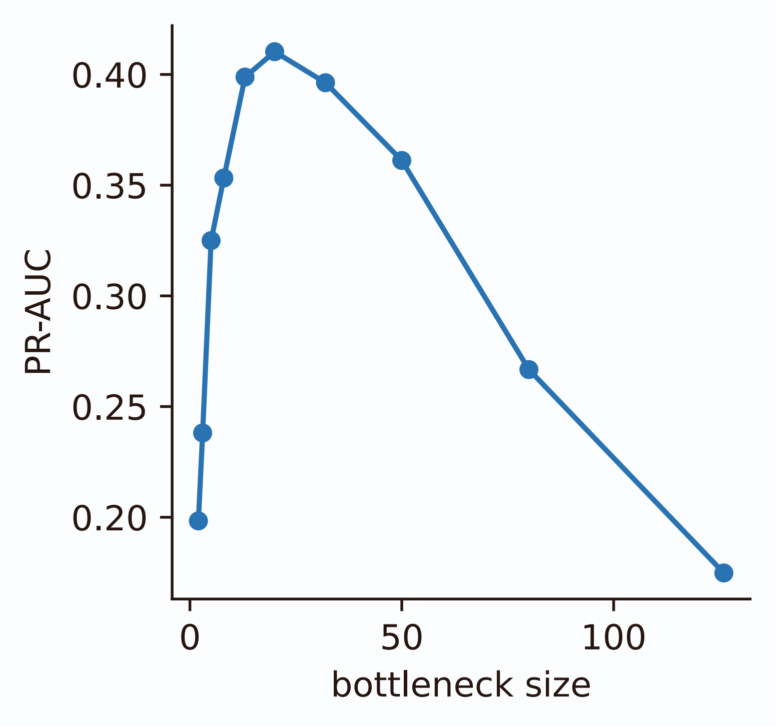

Figure S6. Optimal bottleneck size choice with hyperparameter tuning.
EpiOut detects optimal bottleneck size with artificial outlier injection and hyperparameter tuning. The x-axis indicates the bottleneck size of LR-AE in the EpiOut model, and the y-axis shows the auPRC performance for the artificial outlier prediction.

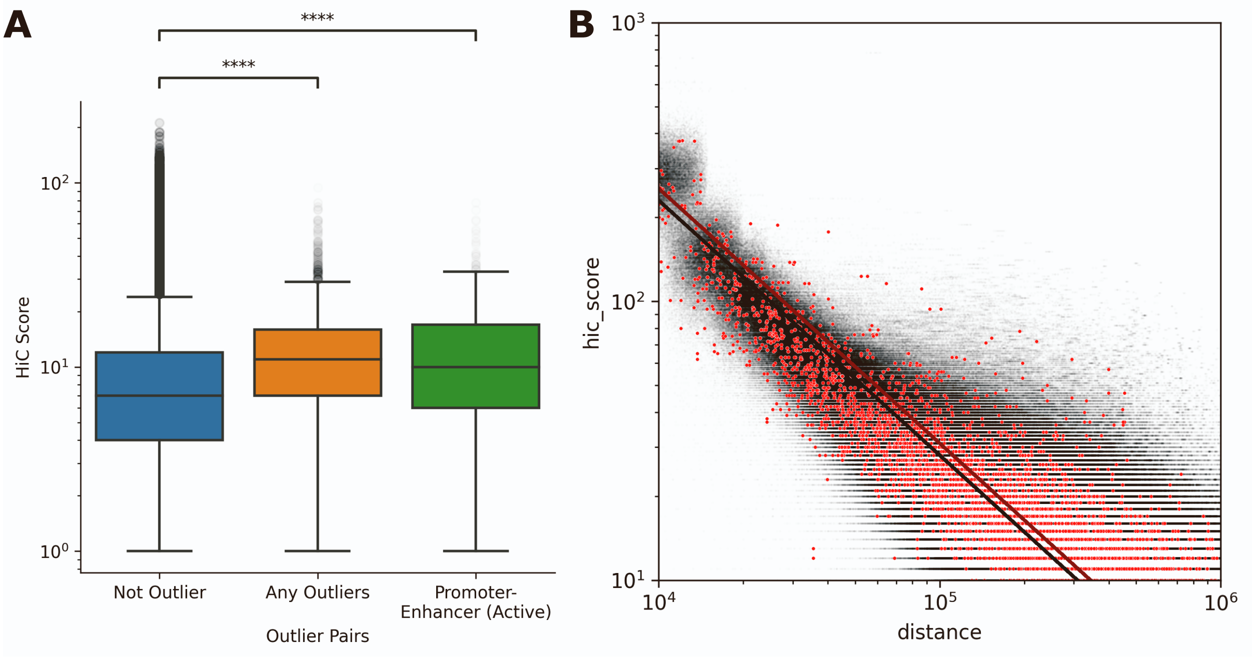

Figure S7. Interaction between outlier pairs.
(A) Hi-C score distribution of accessible regions, which are at least 100 kilo-bp apart, by outlier status and annotation. P-values were calculated with the U-Mann Wily test and corrected for multiple testing using the Bonferroni method. **(B)** Log-log shows the Hi-C scores of non-outlier pairs (in black) and outlier pairs (in red). Hi-C scores decay by power law with increasing distances. We fit a power regression (indicated by black and red lines) on the data to test the interaction between outlier pairs while using distance as a control variable.

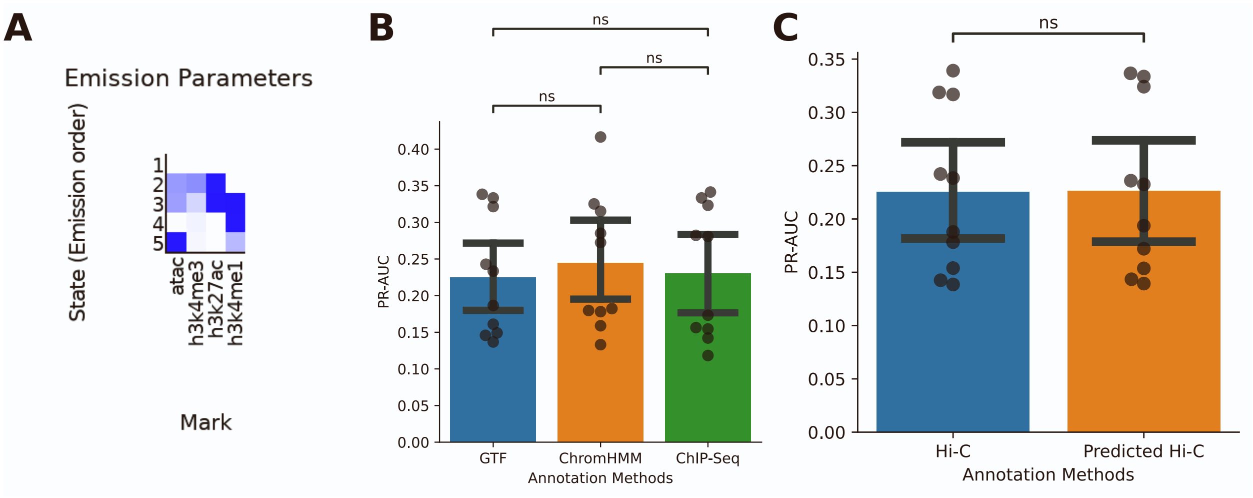

Figure S8. The benchmark of chromatin annotation methods to predict gene expression outliers. **(A)** Emission parameters of ChromHMM. The genome is divided into 200 bp segments and annotated with ChromHMM using 5 hidden states and features of H3K4me3, H3K27ac, H3K4me1, and ATAC-seq. The accessible regions are annotated for one or more states based on their overlap with the segments. **(B)** The performance of alternative annotation methods to predict gene expression outliers. The EBM model was trained and tested with 10-fold cross-validation using the relevant features in each annotation setup**.** The model based on GTF annotation (blue) employs features such as log fold change, p-value, TSS outlier status (as determined by GENCODE annotation), maximum absolute log fold change of proximal chromatin accessibility outliers, and ABC-score weighted absolute log fold change of distal outliers. In contrast, the model utilizing ChromHMM (orange) integrates the same set of features as the blue model, but it further disaggregates the TSS, proximal, and distal features into five distinct features, each corresponding to a specific hidden state identified by ChromHMM. Similarly, the model based on the ChIP-seq marks (green) uses the same features but subdivides the features by promoter, active enhancer, poised enhancer, and region with no chromatin mark. There is no statistically significant difference ($ns \geq 0.05$) between models to predict gene expression outliers based on the Mann–Whitney U test (the PR-AUC for blue $= 22.4 \pm 8.1\%$, orange $= 24.4 \pm 9.1\%$, and green $= 23.1 \pm 8.9\%$). **(C)** The evaluation of gene expression prediction performance, utilizing the ABC-score, was conducted using both observed Hi-C scores (blue) and predicted Hi-C scores (orange). A power regression model (Figure S5B) was employed to compute the Hi-C scores for outlier pairs within a distance of up to 1 million base pairs. There is no statistically significant difference (ns≥0.05) between models to predict gene expression outliers based on the Mann–Whitney U test (PR-AUC for blue $= 22.5 \pm 7.7\%$ and orange $= 22.6 \pm 7.9\%$). The findings suggest that the co-outlier status serves as adequate evidence to infer interactions between accessible regions for predicting gene expression outliers.

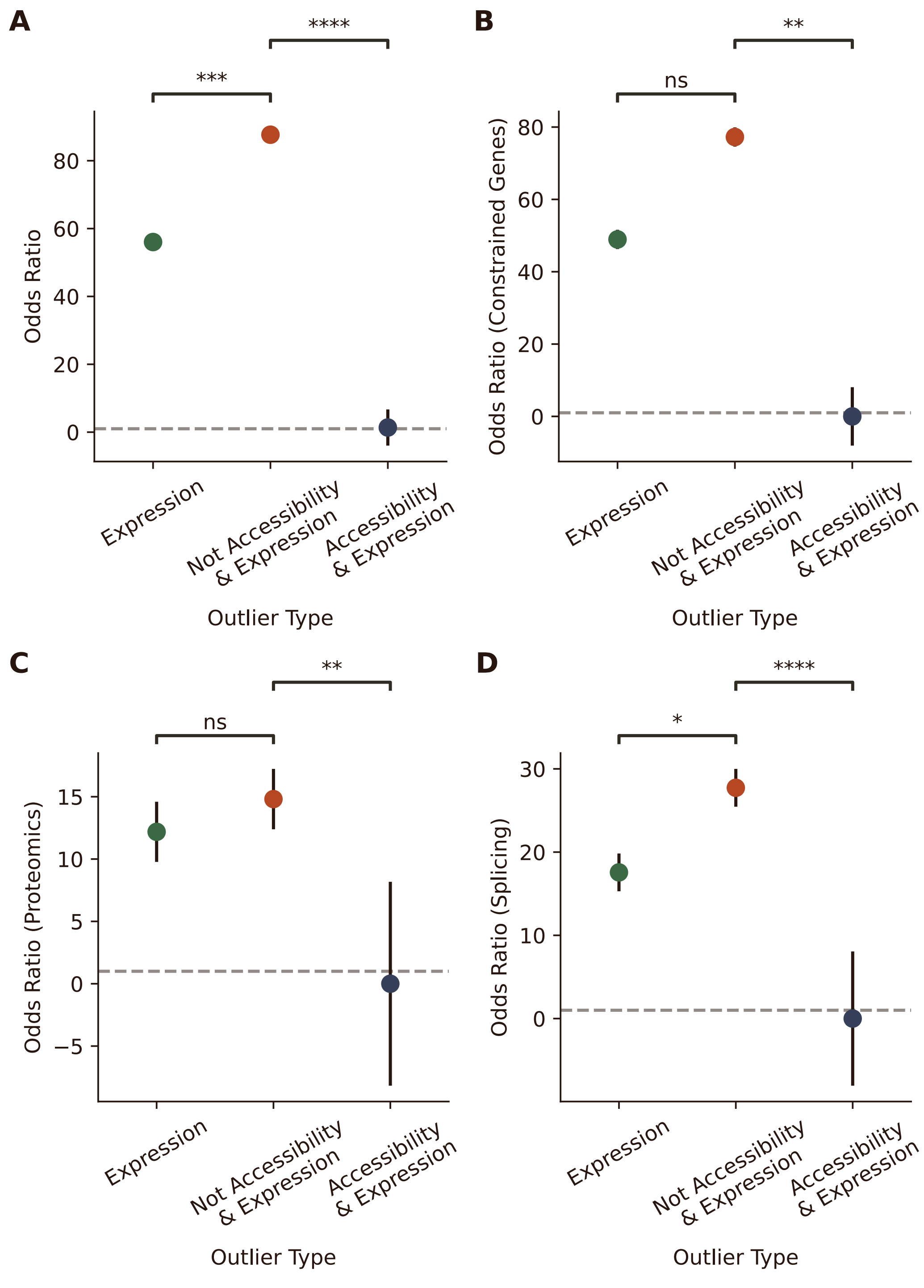

Figure S9: The enrichment and depletion of variants across outlier types by variant effect category. **(A)** Odds of observing potentially NMD-triggering variants in expression outliers (green), expression outliers with non-outlier promoters (red), and expression outliers with outlier promoters are presented. Expression outliers with outlier promoters are strongly depleted of potentially NMD-triggering variants compared to those with non-outlier promoters. Meanwhile, expression outliers with non-outlier promoters are significantly enriched for NMD-triggering variants when compared to the baseline comprising all expression outliers. **(B)** Analysis similar to panel-A but limited to genes under strong

constraint ($\mathrm{LOEUF} < 35\%$) replicates consistent depletion in expression outliers with outlier promoters compared to expression outliers with non-outlier promoters. **(C)** Among genes exhibiting proteomic aberrations (defined by $|Z\text{-score}| > 1$), missense and potentially NMD-triggering rare variants show depletion in genes with outlier promoters relative to gene expression outliers with non-outlier promoters. **(D)** Splicing-disrupting variants predicted by AbSplice are enriched in genes with non-outlier promoters against all gene expression outliers. In contrast, genes with outlier promoters are strongly depleted for the potentially splicing-disrupting variants. ($ns \geq 0.05$,$* < 0.05$,$** < 10^{-2}$,$*** < 10^{-3}$,$**** < 10^{-4}$ based on the hypergeometric test).

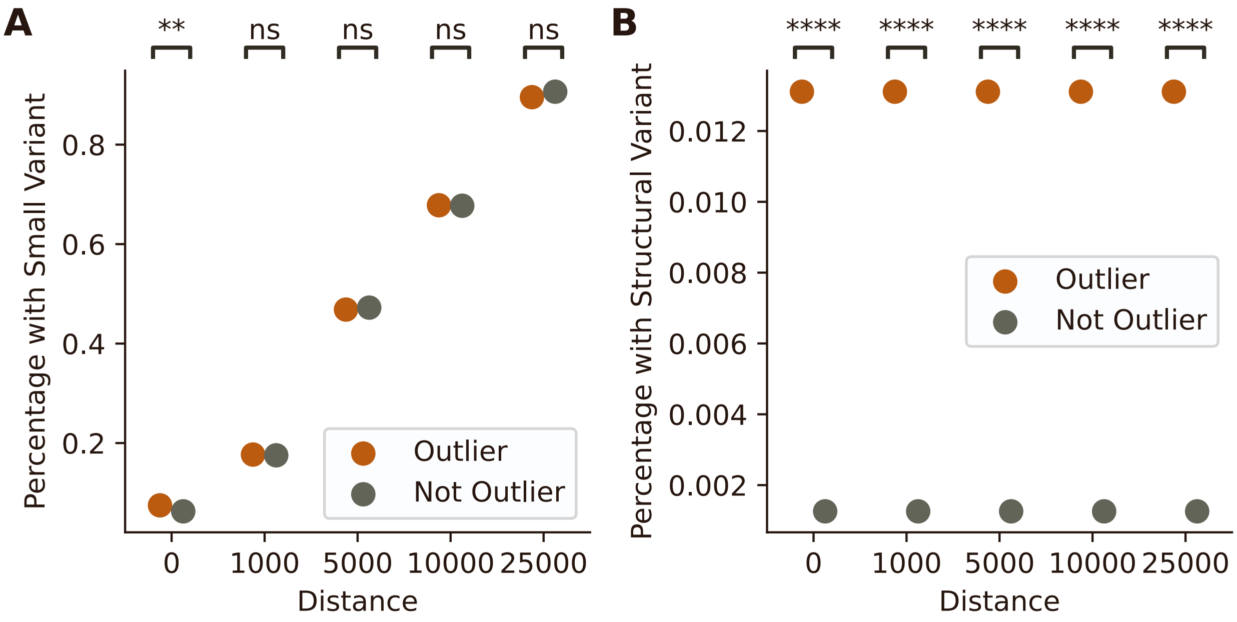

Figure S10: The enrichment of variants in the vicinity of chromatin accessibility outliers (A) Small variants such as SNVs or indels in the accessible chromatin regions are slightly enriched for low frequency and rare variants ($P = 0.002, n = 268, odds\ ratio = 1.2$). However, variants in the vicinity of the chromatin accessibility outliers are not enriched compared to non-outliers. The small effect size can be explained by the fact that the majority of these variants are non-coding and are likely to have weak or neutral regulatory effects. **(B)** Chromatin accessibility outliers are more likely to occur near structural variants ($P < 0.001, n = 47, odds\ ratio = 10.5$). However, only $\sim 1\%$ of chromatin accessibility outliers have at least one structural variant in 1 kbp distance, and $\sim 1.3\%$ have structural variants in proximity.

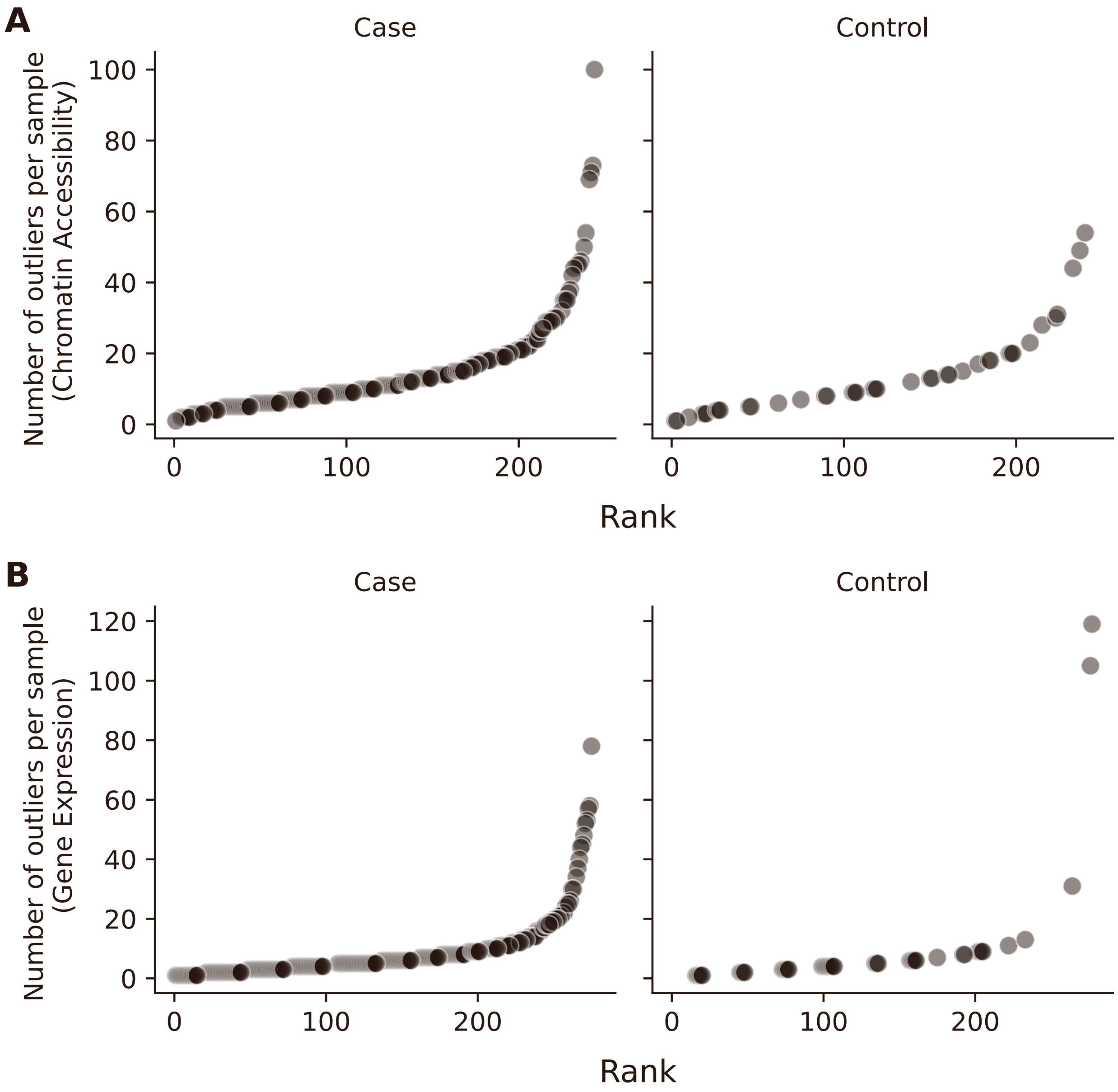

Figure S11: Number of chromatin accessibility and gene expression outliers detected per sample. **(A)** Number of chromatin accessibility outliers per ALS case (11 outliers in median case) **(B)** and clinically healthy controls (10 outliers in median control sample). There is no statistically significant difference in the number of outliers detected between cases and controls ($P = 0.69$, based on the Mann–Whitney U test). **(C)** Number of gene expression outliers per ALS case (6 outliers in median case) **(D)** and controls (4 outliers in median control sample). There is no statistically significant difference in the number of outliers detected between cases and controls ($P = 0.054$, based on the Mann–Whitney U test).

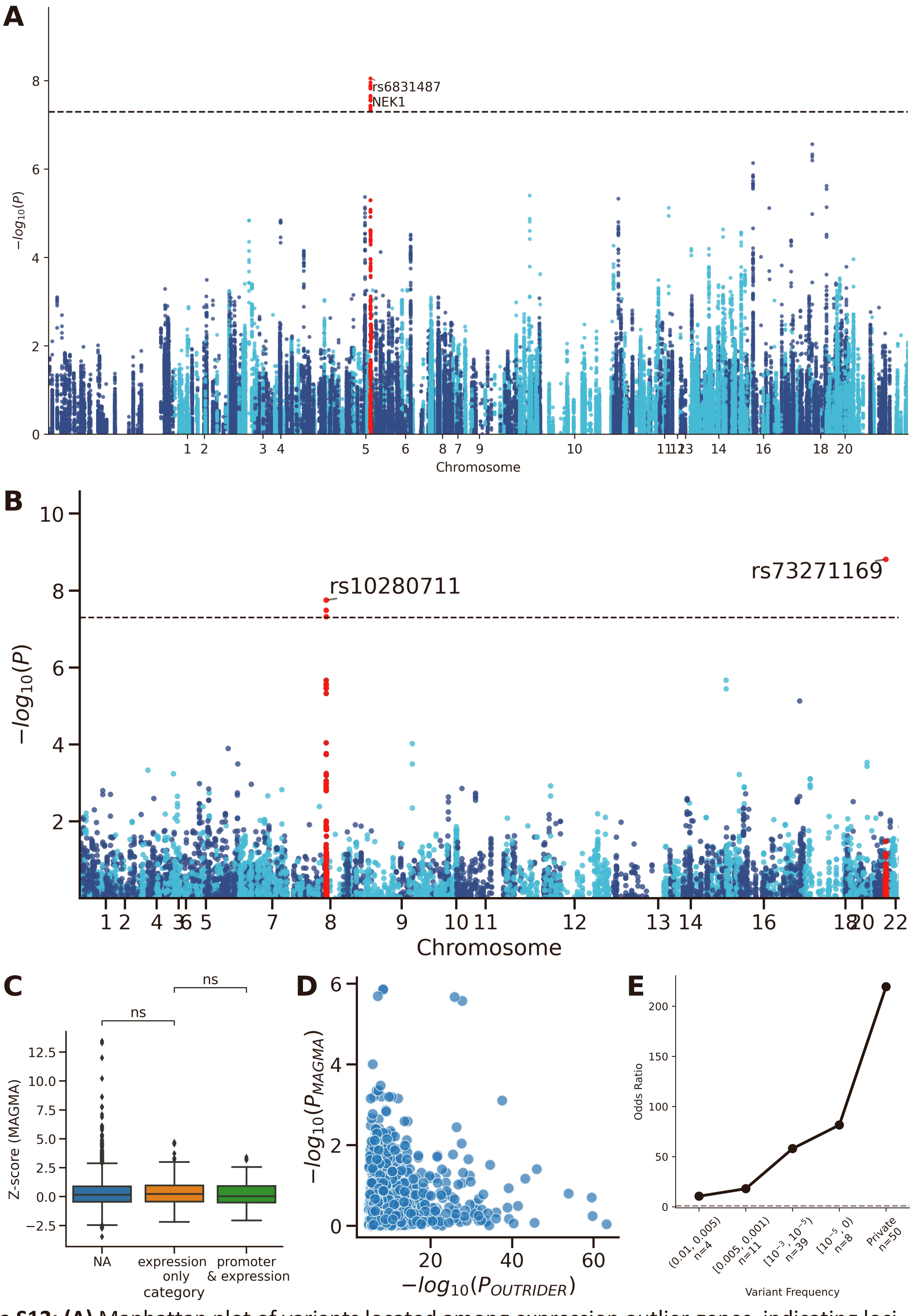

Figure S12: (A) Manhattan plot of variants located among expression outlier genes, indicating loci associated with ALS. The variants are included if observed in the sample with the gene expression outliers. Only one significant locus was found, marked by the most significant variant of rs6831487 near

the NEK1 gene. However, no chromatin accessibility outlier is observed in the vicinity. **(B)** Manhattan plot presenting variants near chromatin accessibility outliers, with two loci identified by variants rs10280711 and rs73271169 in the vicinity of a chromatin accessibility outlier in two ALS cases. However, no gene expression outlier is observed in the vicinity. **(C)** MAGMA Z-score distribution across genes, categorized by outlier status. There is no statistically significant difference between non-outlier genes and the expression of outlier genes with or without outlier promoters. **(D)** OUTRIDER and MAGMA p-values for genes. Genes prioritized based on GWAS and outlier analysis are mostly orthogonal. **(E)** Odds of observing a potentially NMD-triggering rare variant with a specific frequency in a gene expression outlier. The enrichment of rare and private potentially NMD-triggering variants is stronger than the enrichment of low-frequency variants. The results indicated that outlier analysis prioritized the molecular effect of ultra-rare variants, which GWAS may not prioritize.

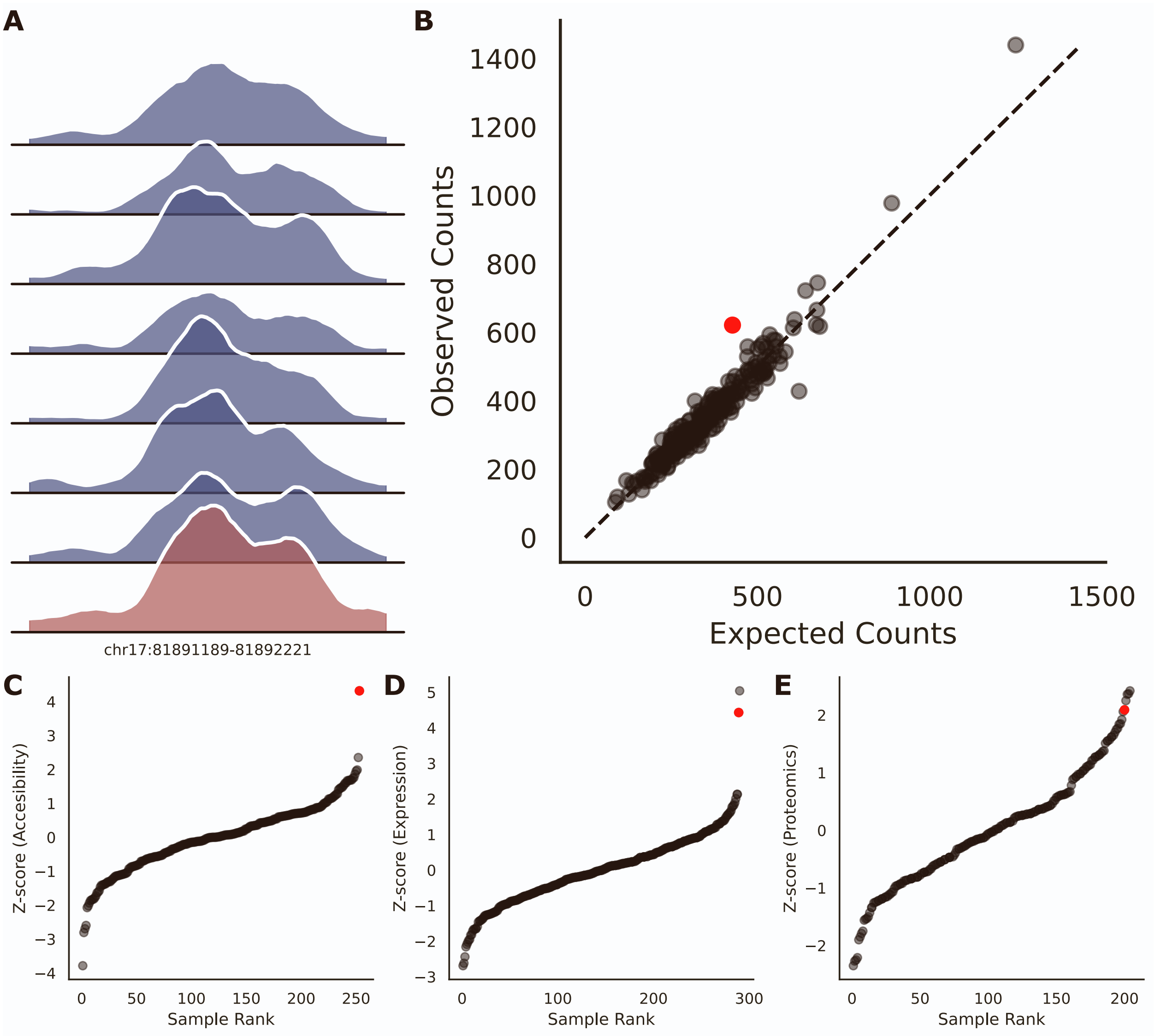

Figure S13. Aberrations observed at multiple omics levels of *ALYREF* gene.

(A) ATAC-seq read coverage at the promoter of the gene. The sample with an outlier promoter is indicated by red across panels. **(B)** Expected and observed accessibility in the promoter of the gene across samples **(C)** Z-score distribution of promoter accessibility **(D)** gene expression **(E)** protein levels across samples.

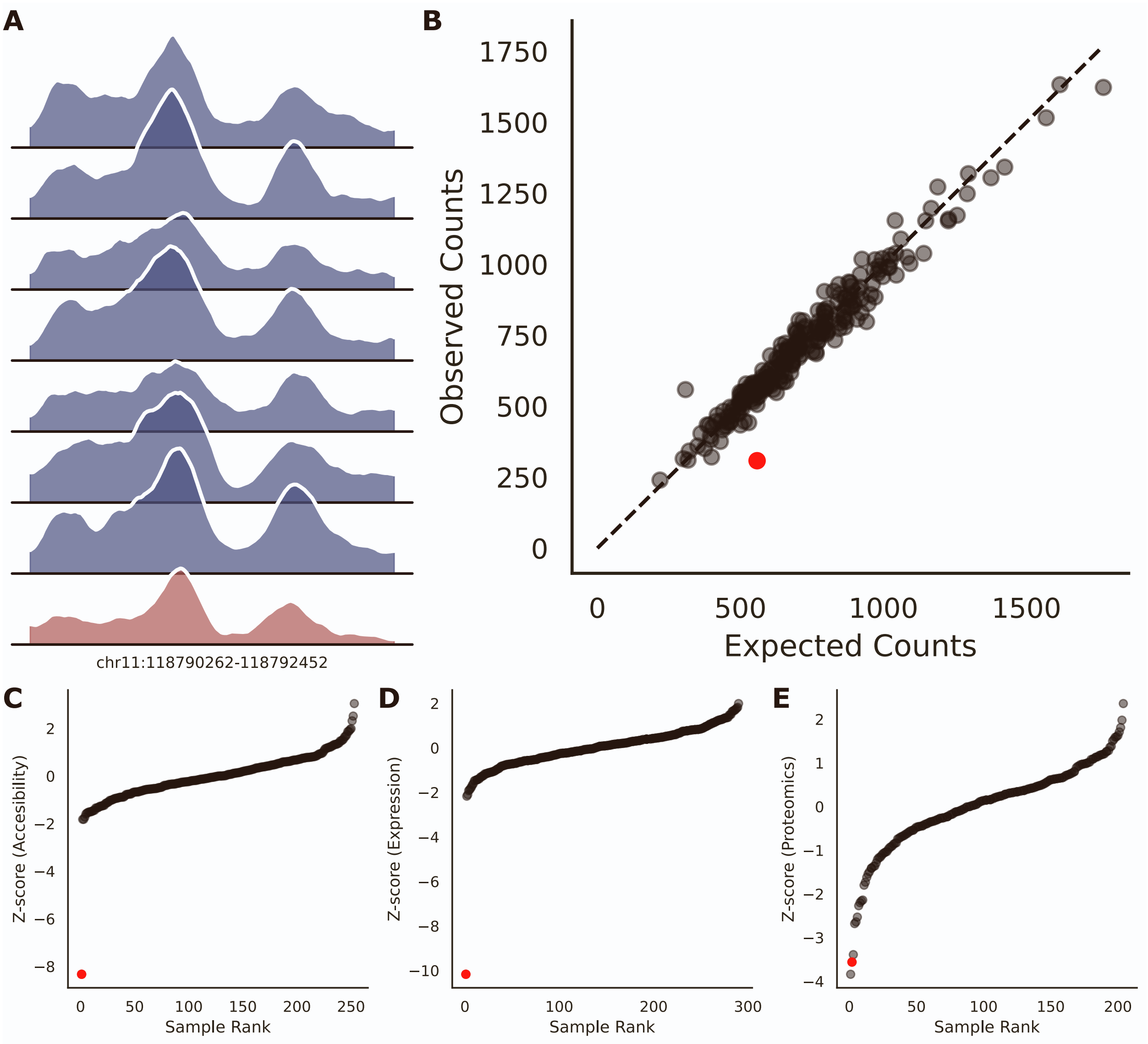

Figure S14. Aberrations observed at multiple omics levels of *DDX6* gene.
(A) ATAC-seq read coverage at the promoter of the gene. The sample with an outlier promoter is indicated by red across panels. **(B)** Expected and observed accessibility in the promoter of the gene across samples **(C)** Z-score distribution of promoter accessibility **(D)** gene expression **(E)** protein levels across samples.

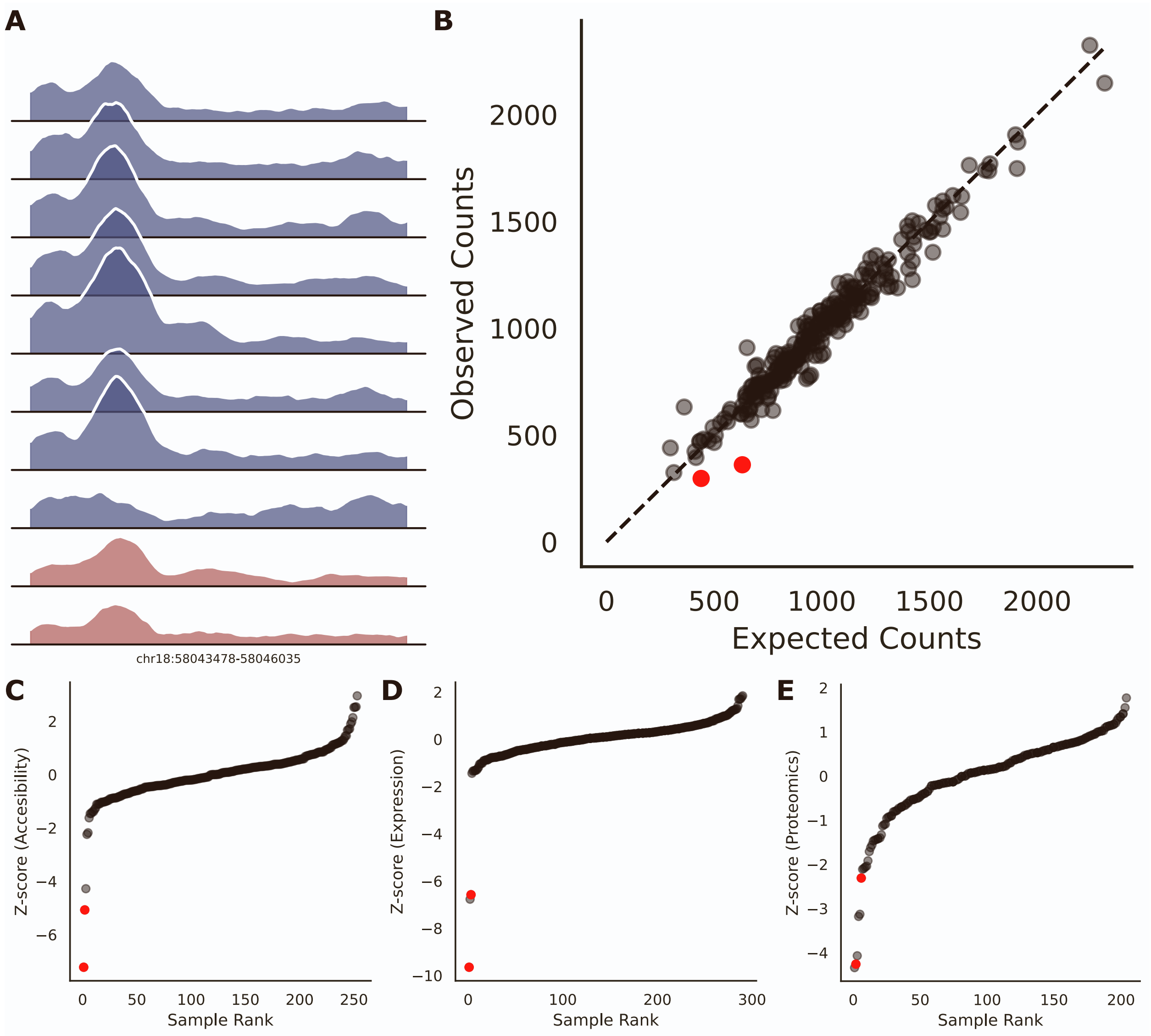

Figure S15. Aberrations observed at multiple omics levels of *NEDD4L* gene.
(A) ATAC-seq read coverage at the promoter of the gene. Samples with an outlier promoter are indicated by red across panels. **(B)** Expected and observed accessibility in the promoter of the gene across samples **(C)** Z-score distribution of promoter accessibility **(D)** gene expression **(E)** protein levels across samples.

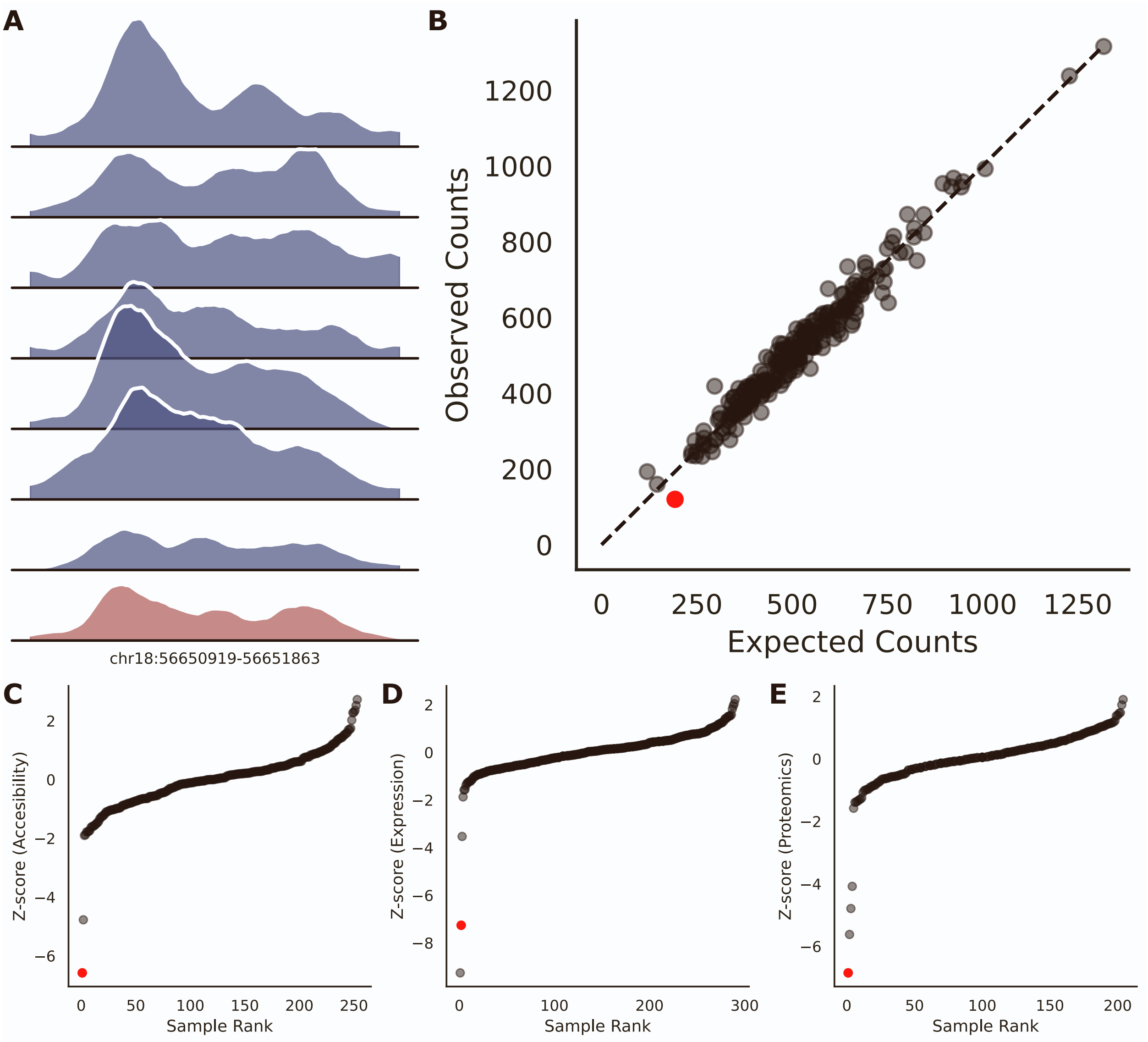

Figure S16. Aberrations observed at multiple omics levels of *TXNL1* gene.
(A) ATAC-seq read coverage at the promoter of the gene. The sample with an outlier promoter is indicated by red across panels. **(B)** Expected and observed accessibility in the promoter of the gene across samples **(C)** Z-score distribution of promoter accessibility **(D)** gene expression **(E)** protein levels across samples.

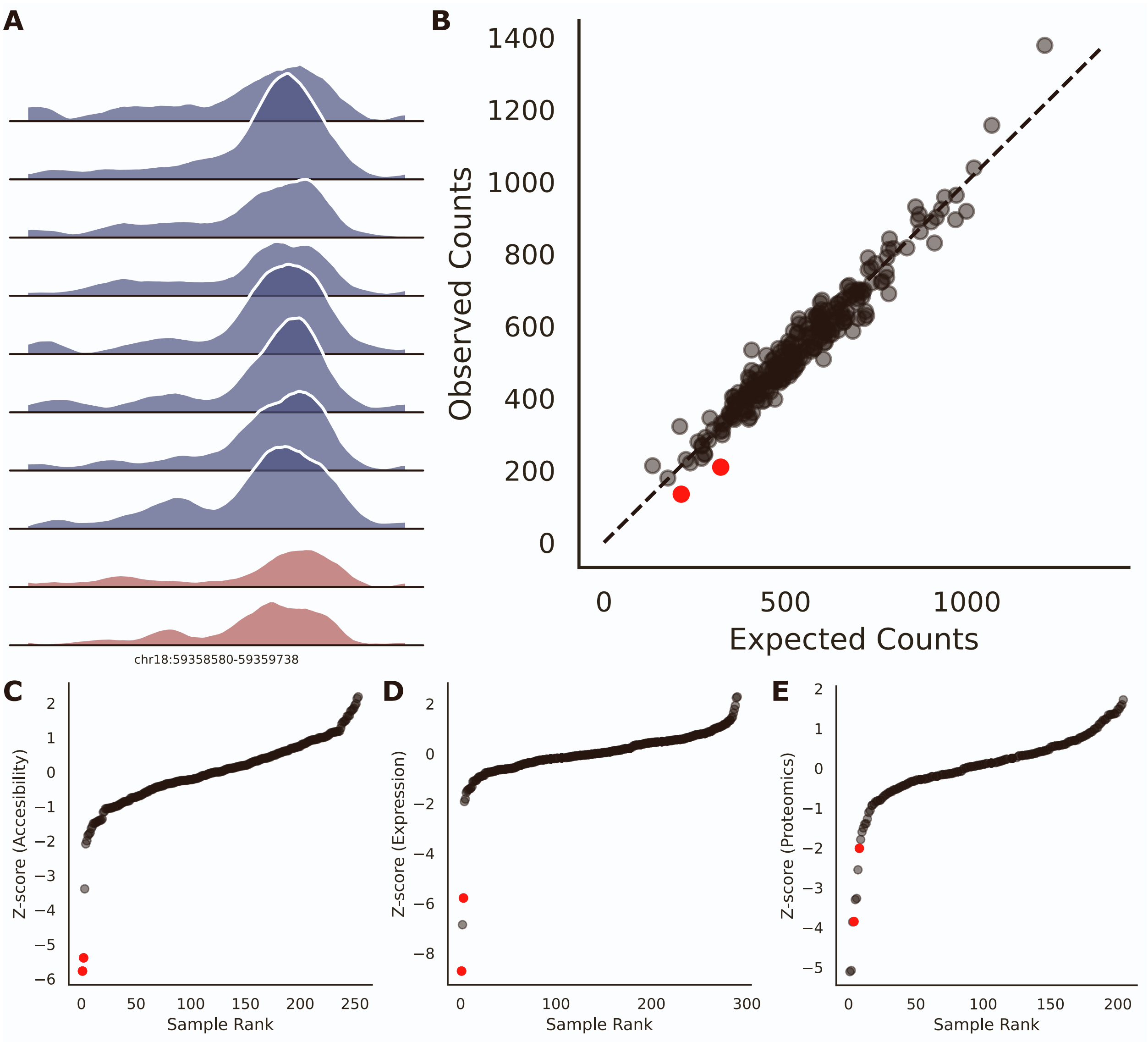

Figure S17. Aberrations observed at multiple omics levels of *LMAN1* gene.
(A) ATAC-seq read coverage at the promoter of the gene. Samples with an outlier promoter are indicated by red across panels. **(B)** Expected and observed accessibility in the promoter of the gene across samples **(C)** Z-score distribution of promoter accessibility **(D)** gene expression **(E)** protein levels across samples.

Table S1. (separate file)

List of chromatin accessibility outliers detected across the AnswerALS samples.

Algorithm S1

Algorithm 1 Read counting algorithm for accessible regions

Require: a bam file and non-overlapping *peaks* sorted by position
Ensure: Read *counts* as a dictionary

for each: $chrom \in chromosomes$
  $peaks \leftarrow$ create a stack by subsetting peaks in $chrom$
  $reads \leftarrow$ create a stack by fetching reads from bam file for $chrom$

  $read \leftarrow reads.dequeue()$
  $peak \leftarrow peaks.dequeue()$

  do
    if $read.end <= peak.start$ **then**
      $read \leftarrow reads.dequeue()$
    else if $read.start >= peak.end$ **then**
      $peak \leftarrow peaks.dequeue()$
      $counts[peak] \leftarrow 0$
    else
      $counts[peak] + +$
      $read \leftarrow reads.dequeue()$
    end if
  while peaks $\neq \emptyset$
